# Fluorinative ring-opening of cyclopropanes by hypervalent iodine reagents. An efficient method for 1,3-oxyfluorination and 1,3-difluorination†
†Electronic supplementary information (ESI) available: Experimental procedures, characterization and NMR spectra of the products. See DOI: 10.1039/c6sc03471c



**DOI:** 10.1039/c6sc03471c

**Published:** 2016-09-16

**Authors:** Nadia O. Ilchenko, Martin Hedberg, Kálmán J. Szabó

**Affiliations:** a Stockholm University , Arrhenius Laboratory , Department of Organic Chemistry , SE-106 91 Stockholm , Sweden . Email: kalman.j.szabo@su.se

## Abstract

A new method is presented for 1,3-difluorination and 1,3-oxyfluorination reactions. The process is based on iodonium mediated opening of 1,1-disubstituted cyclopropanes. The reaction proceeds with high chemo- and regioselectivity under mild reaction conditions typically at room temperature in a couple of hours. The reaction probably occurs *via* electrophilic ring-opening of cyclopropanes.

## Abstract 2

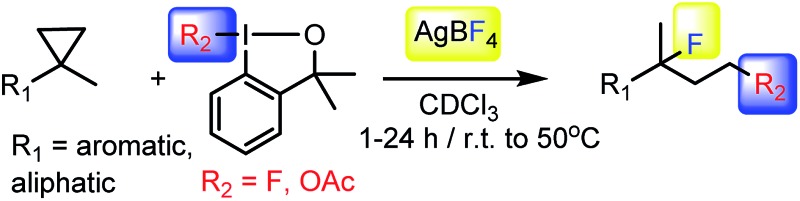
Reaction of 1,1-disubstituted cyclopropanes with hypervalent iodines in the presence of AgBF_4_ leads to 1,3-difluorination and 1,3-oxyfluorination products.

Fluorinated organic compounds have found broad application in the pharmaceutical,^1^ and agrochemical industries^2^ as well as in medical diagnostics.^3^ The impetus for the application of organofluorine compounds in agrochemical and pharmaceutical products is their beneficial pharmacokinetic properties, such as high metabolic stability and lipophilicity.^1a–d^ The useful radionuclear properties of the unnatural isotope ^18^F makes ^18^F labelled organofluoro compounds indispensable for positron emission tomography (PET).^3*a*^ The short half-life of ^18^F requires development of a rapid late stage introduction of the fluorine atom,^3^ which is a challenging task in synthetic organic chemistry.^4^ In the last decade, many new fluorinating reagents have appeared, which in combination of catalysts allowed development of new selective methodologies to access a broad variety of bioactive organofluorines.^4a,5^

The most efficient methods are even suitable for fluorination based difunctionalization reactions.^5b–d^ The most studied approach involves vicinal difunctionalization reactions, such as 1,2-oxyfluorination,^6^ 1,2-aminofluorination,^6a,7^ 1,2-carbofluorination^6a,8^ and related methods.^9^ Recently a number of interesting geminal fluorination methods were also reported, such as 1,1-difluorination,^10^ 1,1-oxyfluorination^11^ and 1,1-aminofluorination.^12^ The 1,2-difunctionalization methods are usually based on alkene substrates, while the 1,1-difunctionalizations are often realized using diazo compounds, as substrates. However, the analogue methodology is much less developed for 1,3-difunctionalization based fluorination methods. Considering the typical synthetic methodologies for 1,3-difunctionalization reactions,^13^ a related fluorination reaction can probably be achieved by ring opening of cyclopropane substrates. Recently, we have shown that hypervalent iodine based^14^ benziodoxol(on) derivatives are excellent reagents for 1,1- and 1,2-difunctionalization for synthesis of organic trifluoromethyl and fluoro compounds.^6a,9a,10a,11,15^ As a part of our concept driven fluorine chemistry program, we sought to employ fluoro-benziodoxol reagent **1a** for a fluorinative ring opening of cyclopropane derivatives. To our delight, **1a** reacted smoothly with cyclopropane derivative **2a** in the presence of AgBF_4_ affording 1,3-difluoro substituted compound **4a** with 71% yield (Scheme 1).

**Scheme 1:**
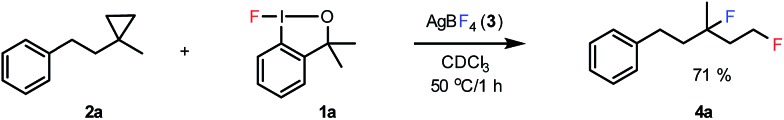
1,3-Difluorination of **2a**.

As we employed **1a** and **2a** in equimolecular ratio in this reaction, one of the fluorine atoms originated from **1a**, while the other one is from the BF_4_^–^ counter ion of the Ag-mediator. We have previously reported^10*a*^ a similar 1,1-difluorination method of styrenes. Although, several chlorination and bromination methods of cyclopropane are reported in the literature,^16^ synthetically useful cyclopropane opening is a very unusual methodology for fluorination reactions. As far as we know the above process is the first 1,3-difluorination reaction. In addition, we have found only a single fluorination based 1,3-difunctionalization reaction in the literature. Very recently, Lectka and co-workers^17^ reported an aminofluorination method based on cyclopropane substrates.

As mentioned above the 1,3-difluorination of cyclopropane **2a** could be carried out selectively and in high yield using **1a** and a stoichiometric amount of AgBF_4_ (Table 1, entry 1) in CDCl_3_. We used CDCl_3_ as the solvent to directly monitor the possible formation of the volatile fluorinated (and other) by-products in the reactions. Replacing AgBF_4_ with AgPF_6_ as a secondary fluorine source led to formation of **4a**, but the yield dropped to 34% (entry 2). Cu(MeCN)_4_BF_4_ can also be used instead of AgBF_4_. The yield was lowered indicating that silver is a better mediator than copper for this transformation (entry 3). However, simple silver sources such as AgF showed to be inactive in 1,3-difluorination reaction (entry 4). Zinc salts have proved to be efficient activators of benziodoxole reagents.^6a,18^ Therefore, we attempted to replace AgBF_4_ with Zn(BF_4_)_2_ but the corresponding reaction did not result **4a** (entry 4). Other silver salts without transferable fluoride in the counter ion, such as AgCN or AgTFA, did not show any activity (entry 5). When a sub-stoichiometric amount (30 mol%) of Ag-salt was used, the yields sharply decreased (entries 6–7). Pd(BF_4_)_2_(MeCN)_4_ (30 mol%) was also inefficient as catalyst (entry 7). Interestingly, Cu(MeCN)_2_BF_4_ showed some catalytic activity but the yield was very low (entry 8). Only traces of product **4a** (<5%) could be obtained with 30 mol% of AgBF_4_ and stoichiometric amount of NaBF_4_ (entry 9). The reaction was completely shut down when KF was employed instead of NaBF_4_ (entry 10). This indicates that the most efficient secondary fluorine source is AgBF_4_. We could not observe any reaction without application of AgBF_4_ or **1a** (entry 11).

**Table 1:** Variation of the reaction conditions for 1,3-difluorination of cyclopropane **2a**

Entry	Deviation from the standard conditions^*a*^	Yield **4a** (%)
1	1 equiv. of **3**	71
2	1 equiv. AgPF_6_ instead of **3**	34
3	1 equiv. Cu(MeCN)_4_BF_4_ instead of **3**	30
4	1 equiv. AgF or Zn(BF_4_)_2_ × H_2_O instead of **3**	<5
5	1 equiv. AgCN or AgTFA instead of **3**	0
6	30 mol% of **3**	9
7	30 mol% AgPF_6_ or Pd(BF_4_)_2_(MeCN)_4_ instead of **3**	<5
8	30 mol% Cu(MeCN)_4_BF_4_ instead of **3**	15
9	30 mol% **3** and 1 equiv. NaBF_4_	<5
10	30 mol% **3** and 1 equiv. KF	0
11	Without **3** or **1a**	0
12	1 equiv. Selectfluor or NFSI instead of **1a**	0
13	Without **3**, 1 equiv. of Tol-IF_2_ instead of **1a**	24
14	DCM instead of CDCl_3_	10
15	MeCN or MeOH instead of CDCl_3_	0

^*a*^Reagent **1a** (0.1 mmol) cyclopropane **2a** (0.1 mmol) and AgBF_4_ (**3**) (0.1 mmol) were mixed in CDCl_3_ (0.5 ml). This mixture was stirred at 50 °C for 1 h.

Neither Selectfluor nor NFSI could replace fluoroiodoxol **1a** as the electrophilic fluorination reagent (entry 12). When benziodoxole based **1a** was replaced by 4-iodotoluene difluoride (Tol-IF_2_), a related hypervalent iodine reagent,^14*a*^ product **4a** did not form at all. Unlike **1a**, Tol-IF_2_ underwent rapid decomposition in the presence of AgBF_4_. When the reaction was performed in the absence of AgBF_4_ (**3**) with Tol-IF_2_ a complex reaction mixture was obtained, from which compound **4a** could be isolated in 24% yield (entry 13). In general, we found Tol-IF_2_ much less bench-stable than **1a** and more prone to providing complex product mixtures.

A brief solvent screen has shown that dichloromethane is a less suitable solvent providing the product in 10% yield (entry 14). However formation of product **4a** was not observed when chloroform was replaced by acetonitrile or methanol (entry 15).

Subsequently, we investigated the synthetic scope of the silver mediated 1,3-difluorination reaction (Table 2). We found that several substrates required longer reaction times for full conversion relative to **2a** (Table 2, entry 1). Under an elongated reaction time **1a** underwent partial decomposition (see below). Therefore, in most reactions we employed two equivalents of **1a** to obtain a full conversion of **2** and, thus optimal yields of **4**. Aliphatic substrate **2b** reacted for 4 h at room temperature affording **4b**. Dialkyl cyclopropanes such as, 1,1-dibutyl cyclopropane **2c** also reacted affording **4c** (entry 3). In this case the yield was lower than for difluorination of **2b** indicating that the reaction is fairly sensitive to the steric factors of the cyclopropane substituents. We have studied the reactivity of aryl substituted cyclopropanes as well. 1,1-Dipenyl cyclopropane **2d** is a particularly challenging substrate. It is sterically hindered and the fluorine expected to enter to a dibenzylic position. We found that **2d** reacted relatively quickly (2 hours) with **1a** resulting in **4d** (entry 4) in 47% yield. As expected **4d** had a limited stability, which could explain the relatively low yield. A possible reason for the poor stability is the easy dissociation of the fluoride from the dibenzylic position. When one of the phenyl groups in **2d** was changed to a methyl group, **2e**, the reaction required a longer reaction time (6 hours), however the yield of the corresponding product **4e** was higher, 59% (entry 5). Product **4e** was also more stable than **4d** probably because of the stronger quaternary C–F bond. In the presence of electron donating group in the *para* position of the aromatic substituent, **2f**, we obtained a fast fluorination reaction (only 1 hour at room temperature) affording **4f** in 55% yield (entry 6). Apparently electron donating groups accelerate the reaction. Naphthyl substituted substrate **2g** also reacted smoothly to give **4g** in 65% yield (entry 7). The rate of the reaction was much slower in the presence of an electron withdrawing group (*e.g.* **2h**) than for electron donating group (*e.g.* **2f**) in the *para* position of the aryl substituent. Thus, *para*-bromo substituted **2h** had to be reacted 24 hours to provide **4h** (entry 8), while the reaction of *para* phenyl substituted substrate **2f** was compete in 1 hour (entry 6). Similarly to the aliphatic substrates (*e.g.* **2c**) the difluorination reaction can be carried out for longer homologues of the methyl substituents. For example **2i–j** reacted with high yields affording difluorinated products **4i–j** (entries 9–10). The presented 1,3-difluorination method can be easily scaled up by five times without significant change in yield (entry 1).

**Table 2:** Silver mediated 1,3-difluorination with **1** ^*a*^

Entry	Substrate	*t* (h)	Product	Yield^*b*^ (%)
1	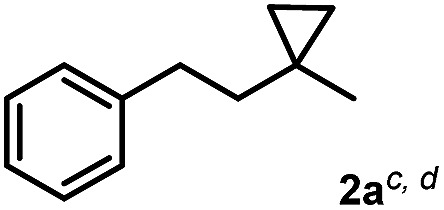	1	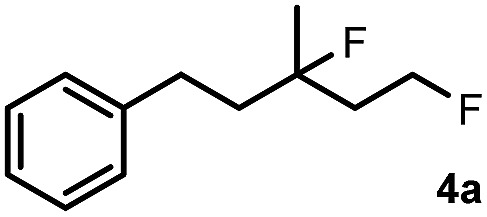	71 (67%)^*e*^
2	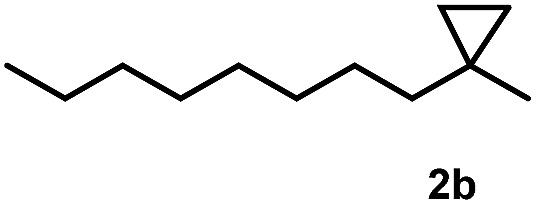	4	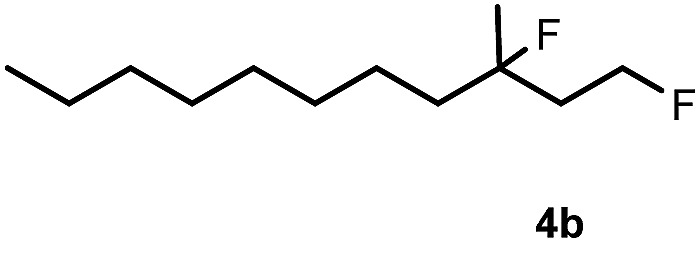	70
3	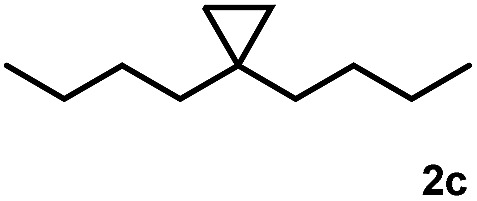	4	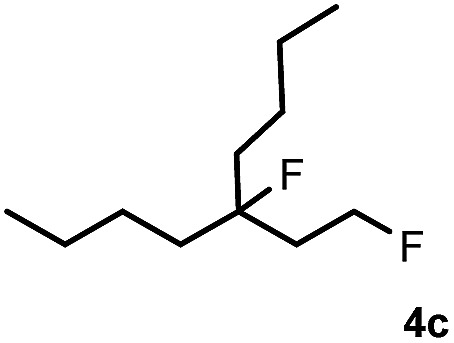	51
4	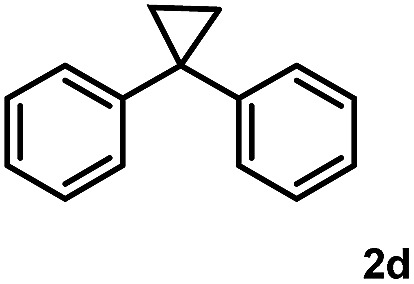	2	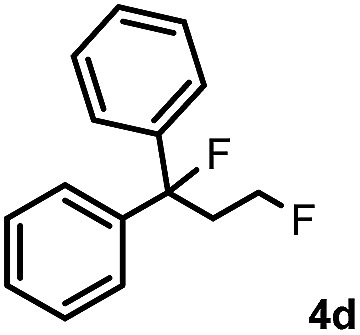	47
5	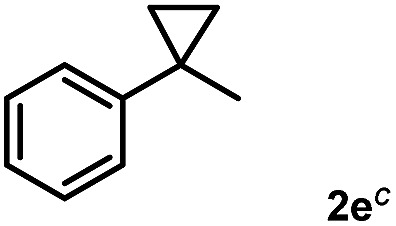	6	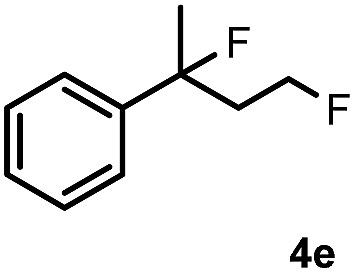	59
6	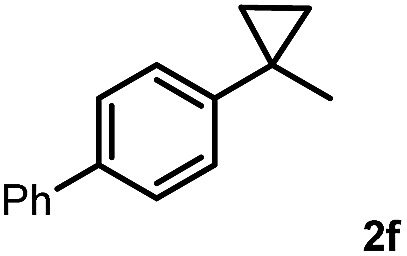	1	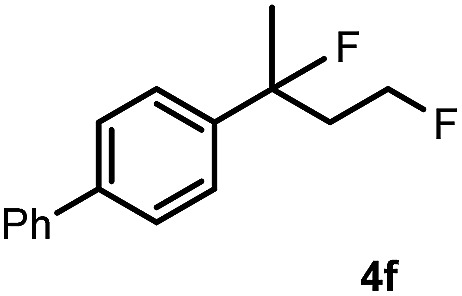	55
7	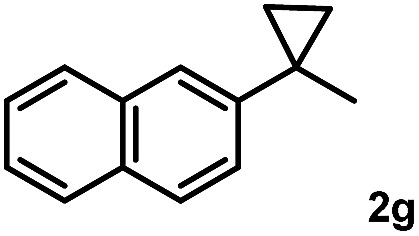	3	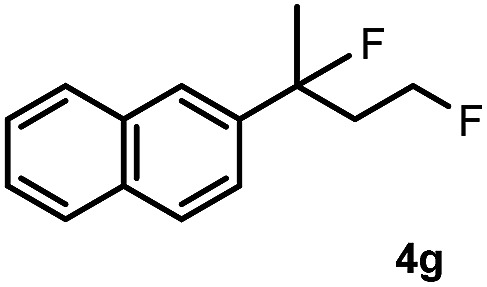	65
8	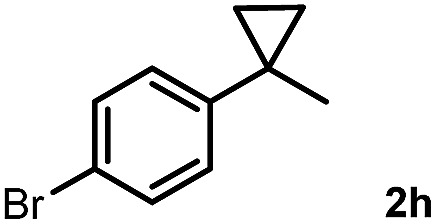	24	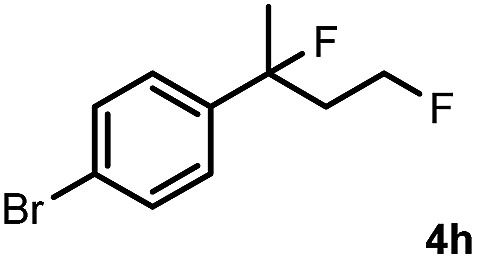	57
9	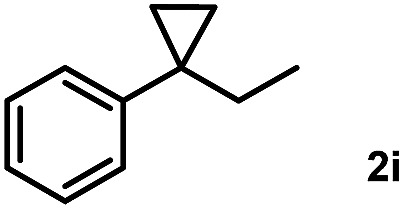	24	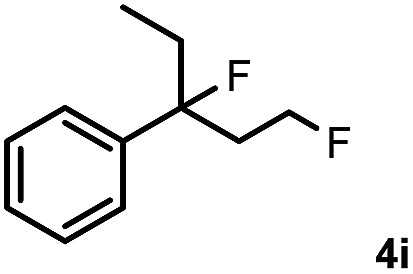	70
10	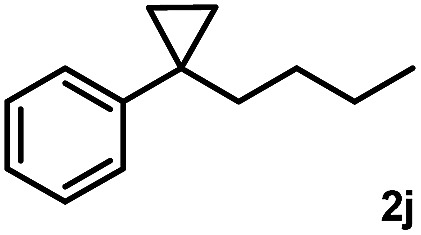	24	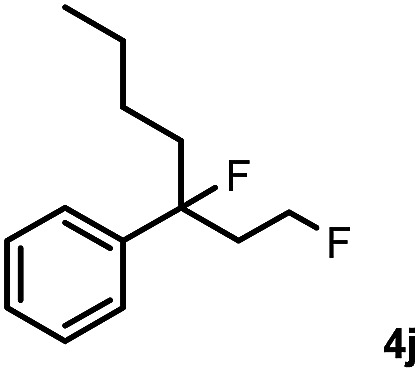	66

^*a*^Unless otherwise stated, substrate **2** (0.1 mmol), **1a** (0.2 mmol) and AgBF_4_ (**3**) (0.1 mmol) in CDCl_3_ (0.5 ml) were stirred at room temperature.

^*b*^Isolated yields.

^*c*^(0.1 mmol) of **1a** was used.

^*d*^The reaction was performed at 50 °C.

^*e*^The reaction was performed in 0.5 mmol scale.

Table 2 shows that the above reaction is suitable for the synthesis of quaternary 1,3-difluoro compounds **4a–j** from 1,1-disubstituted cyclopropanes **2a–j**. However, when we attempted to react 1,2-disubstituted cyclopropanes, we obtained very complex, inseparable mixtures with several fluorinated products. The observation that this reaction proceeds faster in the presence of electron donating and/or aryl substituents on the cyclopropane moiety suggests an electrophilic fluorinative cyclopropane opening mechanism. As mentioned above (Scheme 1, Table 1) the overall reaction can be regarded as a formal introduction of an F_2_ molecule into the cyclopropane substrates. The electrophilic fluorine atom (formally F^+^) supposedly comes from reagent **1a**, while the nucleophilic fluorine atom (formally F^–^) from the BF_4_^–^ counter ion.^19^ Considering this hypothesis, we attempted to introduce fluorine and a different functionality to cyclopropanes applying this concept.

When we replaced fluoroiodoxole **1a** with acetoxyiodoxole **1b**, the reaction with **2a** resulted in 1,3-oxyfluorinated product **5a** (Table 3, entry 1) in 84% yield. In this reaction, we did not observe formation of difluorinated product **4a**. In addition, the regioselectivity was also very high as we could not detect formation of the regioisomer of **5a**. Aliphatic and aryl substrates **2b** and **2f** also reacted with the same chemo- and regioselectivity as **2a** (entries 2 and 3). Products **5b–c** had a limited stability, and decomposed within a couple of hours at room temperature. Instead of **1b**, **1c** (PIDA) could also be employed as acetoxy source. In this reaction, we also obtained **5a** in good yield (entry 4) without formation of diacetoxy or difluoro (**4a**) analogues. Interestingly, **1c** reacted much faster (20 min) than the iodoxole analogue **1b** (4 hours). When benzoyl analogue **1d** was used benzoyl product **5d** formed instead of **5a** (entry 5).

**Table 3:** Silver mediated 1,3-oxyfluorination^*a*^

Entry	Substrate	Hypervalent iodine	*t* (h)	Product	Yield^*b*^ (%)
1	**2a**	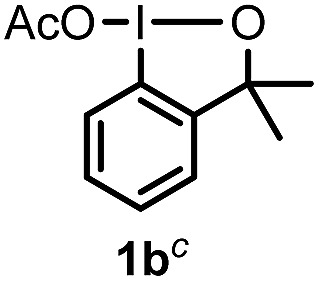	4	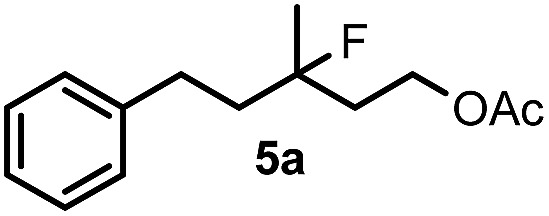	84
2	**2b**	**1b**	2	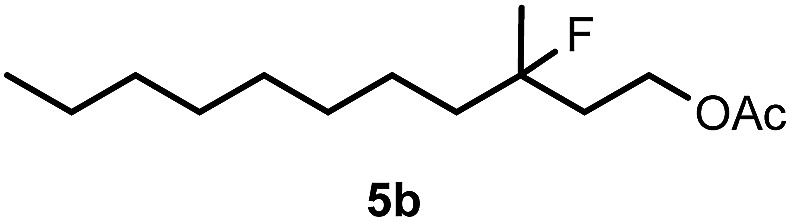	50
3	**2f**	**1b**	2	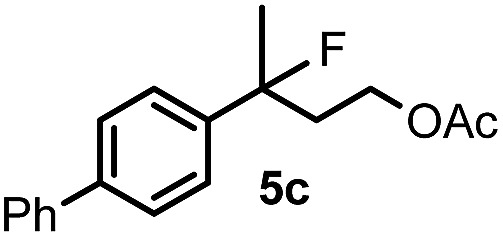	51
4	**2a**	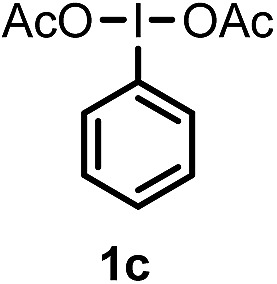	20 min	**5a**	84
5	**2a**	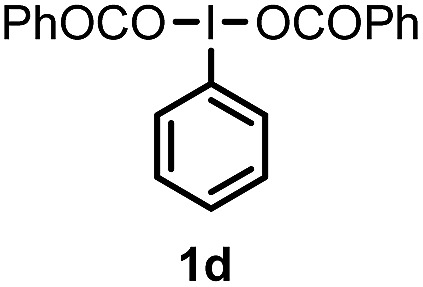	20 min	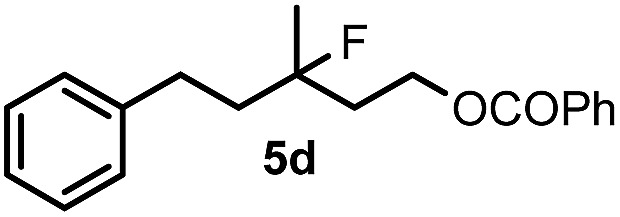	80
6	**2a**	**1a**/**6** ^*d*^	18	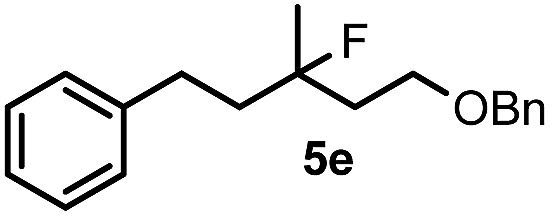	80
7	**2a**	**1a** ^*e*^/**6** ^*d*^	18	**5e**	17
8	**2b**	**1a**/**6** ^*d*^	18	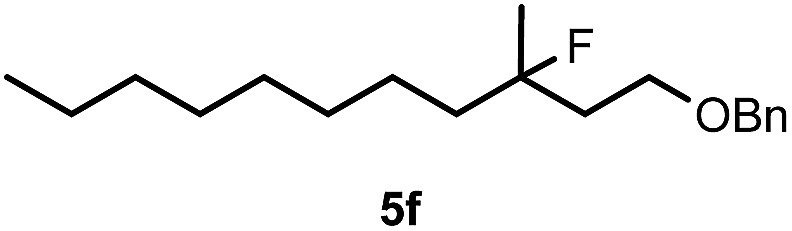	91

^*a*^Unless otherwise stated, substrate **2** (0.1 mmol), **1** (0.1 mmol) and AgBF_4_ (**3**) (0.1 mmol) in CDCl_3_ (0.5 ml) were stirred at room temperature.

^*b*^Isolated yields.

^*c*^(0.2 mmol) of **1b** was used.

^*d*^Substrate **2a–b** (0.1 mmol), **1a** (0.1 mmol), AgBF_4_ (**3**) (0.1 mmol) and BnOH (**6**) (0.3 mmol) in CDCl_3_ (0.5 ml) were stirred at room temperature.

^*e*^AgBF_4_ (**3**) (30 mol%).

Cyclopropane derivatives **2a** and **2b** were also reacted with fluoroiodoxole **1a** in the presence of benzyl alcohol (**6**) and AgBF_4_. In these reactions the final products were 1,3-oxyfluorinated species **5e–f** (Table 3, entries 6–8) instead of **4a–b** (Table 2, entries 1–2), which were formed in the absence of benzyl alcohol. Since in oxyfluorination only a single fluorine is introduced, we attempted to react **2a** and **1a** in the presence of benzyl alcohol and sub-stoichiometric amount of AgBF_4_ **3** (entry 7). However, the yield of the oxyfluorinated product **5e** substantially decreased (*c.f.* entries 6 and 7). Apparently, application of stoichiometric amount of AgBF_4_ is required, for both as a source for the secondary fluorine atom in the difluorination reaction (such as for formation of **4a**) and also in the oxyfluorination reaction for efficient activation of **1a**. In the oxyfluorination reactions the activated hypervalent iodine reagents proved to be more stable than in the difluorination reactions. Therefore, in most processes (entries 2–7) one equivalent of the iodine reagent was sufficient to obtain the reported isolated yields.

In order to obtain more insight into the electronic effects of the reactions and the role of the applied hypervalent iodine, we performed a couple of control experiments. When an equimolar ratio of **2e**, **2h** and **1a** reacted in the presence of AgBF_4_, we obtained only **4e**, while formation of **4h** was not observed (Scheme 2). This competitive reaction indicates that cyclopropane substrates bearing an electron withdrawing group, such as **2h**, react much slower than the parent compound **2e**.

**Scheme 2:**
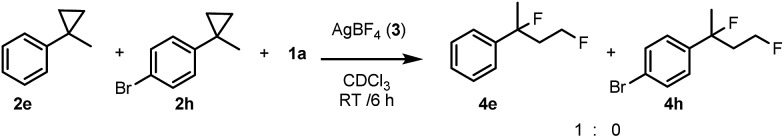
Competitive fluorination using equimolar ratio of **2e**, **2h** and **1a**.

This confirms the suggestion of the electrophilic mechanism for the opening of the cyclopropane ring. When **2a** was reacted with equimolar amounts of fluoro- (**1a**) and acetoxyiodoxoles (**1b**) products **4a** and **5a** were formed in 1 : 2 ratio (Scheme 3) indicating that the oxidation power or the electrophilicity of the hypervalent iodine is an important factor for the reaction rate.

**Scheme 3:**
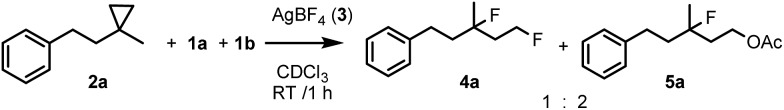
Competitive 1,3-difluorination *vs.* 1,3-oxyfluorination using equimolar amounts of **2a**, **1a** and **1b**.

Considering the above and the literature data for related reactions,^6a,9a,10a^ we propose a plausible mechanism for the fluorinative opening of cyclopropanes with hypervalent iodines (Scheme 4). Benziodoxole reagents **1a–b** are stable^6*b*^ under ambient conditions, and usually require activation in the substitution and addition reactions.^6a,9a,10a^ We suggest that AgBF_4_ activates **1a–b** by coordination of the oxygen atom of the benziodoxole ring to the silver cation affording intermediate **7**. Similar, Lewis-acid type of activation of benziodoxoles was reported by Togni and co-workers.^18^ Unlike, **1a–b**, activated benziodoxole **7** is very reactive, and besides the desired fluorination reaction it may undergo decomposition (or other side-reactions). This is the reason for application of two equivalents of **1a** in some difunctionalization reactions where the substrate has a low reactivity or the rate of decomposition of intermediate is high. We suggest that **7** undergoes side-attack of the cyclopropane ring (**8**) to give carbocationic intermediate **9** and iodobenzene derivative **10**. This mechanism is reminiscent of our proposal for the difluorination of styrenes with **1a**.^10*a*^ The high regioselectivity of the attack is an interesting feature of the process (Table 3). A possible explanation is that the regioselectivity is controlled by electronic effects, *i.e.* hyperconjugative stabilization of the tertiary carbocation center. The final step of the process could be a nucleophilic attack by fluorine from the BF_4_^–^ counterion^19^ to obtain the final product (**4** or **5**).

**Scheme 4:**
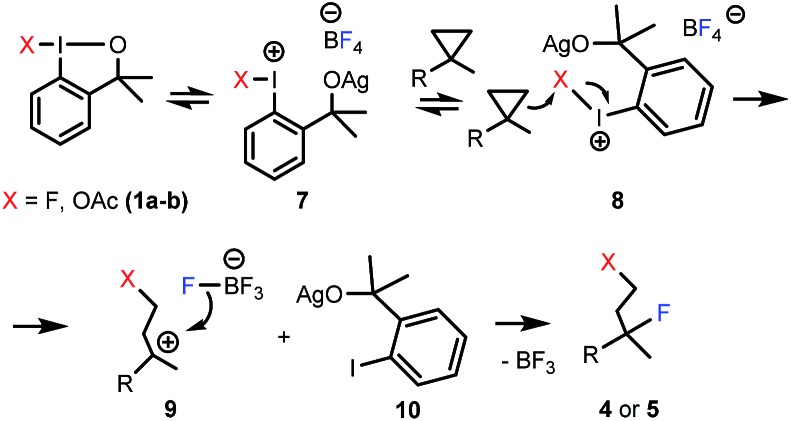
Plausible mechanism for 1,3-difluorination and 1,3-oxyfluorination reactions.

In case of oxyfluorination with benzyl alcohol (Table 3, entries 6–8) **1a** was probably reacted with **6** prior to the ring opening providing benzyloxy-benziodoxole (analogue to **1b**). In this case intermediate **9** is a benzyl ether (X = OBn). This idea is supported by the control experiment (Scheme 5), in which, we first performed a difluorination affording **4a**, then **6** was added. In this reaction we obtained **5g**, which is the regioisomer of **5e** (see Table 3, entry 6).

**Scheme 5:**
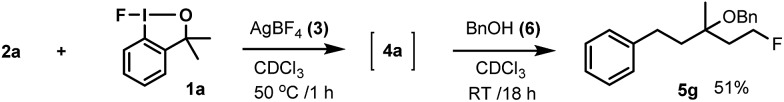
Sequential oxyfluorination reaction with **2a**.

Accordingly, when **2a**, **1a**, **3** and **6** were mixed at the onset of the reaction (Table 3, entry 6) difluorination product **4a** did not form. This reaction lead to the formation of **5e** directly (according to the mechanism outlined in Scheme 4). If **4a** formed first in the process, benzyl alcohol (**6**) would have displaced the tertiary fluorine affording **5g** (Scheme 5).

Modelling and experimental studies are underway to explore the mechanistic details of the above and related^6a,9a,10a^ metal mediated reactions of fluoro-benziodoxol reagent **1a**.

In conclusion, we have shown that the air- and moisture stable fluoroiodine reagent **1a** is suitable for the silver mediated 1,3-difluorination reaction of 1,1-disubstituted cyclopropanes. The reaction can be extended to 1,3-oxydifluorination by using hypervalent acetoxy and benzoyloxy iodines. The reaction probably proceeds *via* electrophilic ring opening of cyclopropanes. As the above process is the first 1,3-difluorination and 1,3-oxydifluorination reaction, it broaden the synthetic scope of the fluorination reactions, and the application area of hypervalent fluoroiodines.

## Conflict of interest

The authors declare no competing financial interests.
