# Supplementary material for: Fluorinative ring-opening of cyclopropanes by hypervalent iodine reagents. An efficient method for 1,3-oxyfluorination and 1,3-difluorination
Source: Chem Sci. 2016 Sep 16;8(2):1056–61. doi: 10.1039/c6sc03471c (PMC5356504; doi:10.1039/c6sc03471c)
Supplement: Supplementary file 1 [file SC-008-C6SC03471C-s001.pdf]

Electronic Supplementary Material (ESI) for Chemical Science.
This journal is © The Royal Society of Chemistry 2016

Supporting Information

Fluorinative Ring-opening of Cyclopropanes by Hypervalent Iodine Reagents. An Efficient Method for 1,3-Oxyfluorination and 1,3-Difluorination

Nadia O. Ilchenko, Martin Hedberg and Kálmán J. Szabó*

Stockholm University, Arrhenius Laboratory, Department of Organic Chemistry SE-106 91 Stockholm, Sweden. E-mail: kalman@organ.su.se. Fax: +46-8-15 49 08

Contents:

General information 1

General procedure for preparation of cyclopropanes 1

General procedure for 1,3-difluorination of cyclopropanes 3

References 10

^{1}H, ^{13}C and ^{19}F NMR spectra 11

General information

Hypervalent iodine reagents **1a,**[1] **1b,**[2] **1d**[3] and alkenes **11a-j**[4] were prepared according to literature procedures. All other chemicals were obtained from commercial sources and used as received. ^{1}H NMR, ^{13}C NMR and ^{19}F NMR spectra were recorded in $CDCl_3$ (internal standard 7.26 ppm, ^{1}H; 77.2 ppm, ^{13}C) using 400 MHz spectrometers. For column chromatography, silica gel (35-70 microns) was used. Unless otherwise stated, all the reactions were performed under Argon atmosphere.

General procedures for the preparation of cyclopropanes

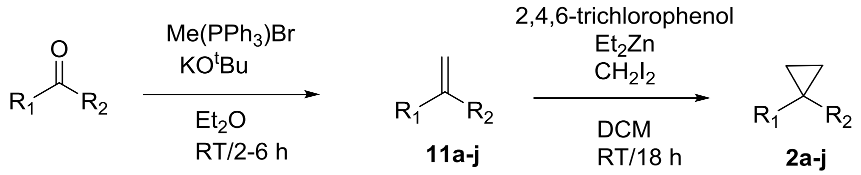

General procedure for the preparation of cyclopropanes

According to a modified procedure by Charette and co-workers,[5] diethylzinc (10 mmol) was added to the solution of 2,4,6-trichlorophenol (10 mmol) in CH_2Cl_2 (60 ml) at -40°C. Then, the solution was stirred for 15 minutes and diiodomethane (10 mmol) was added. After stirring for another 15 minutes, the corresponding alkene **11a-j** (5 mmol) was added and the reaction mixture was stirred in room temperature for 12 h. Then, the organic phase was washed with 10% aq. HCl (2 x 25 ml), saturated aq. $NaHCO_3$ (2 x 25 ml) and brine (25 ml). The organic phase was dried over $MgSO_4$ and concentrated. Cyclopropanes were isolated by silica gel column chromatography in pentane. Compounds **2g**,[6a] **2h**,[6b] and **2i**[6c] were confirmed by NMR comparison to reported data.

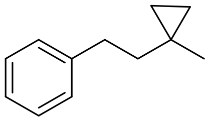

(2-(1-Methylcyclopropyl)ethyl)benzene (2a)

This product was prepared according to the above general procedure. Compound **2a** was obtained as transparent oil (1.2 g, 78%). ^{1}H-NMR (400 MHz, $CDCl_3$) δ 7.30-7.26 (m, 2H), 7.20-7.16 (m, 3H), 2.62-2.81 (m, 2H), 1.56-1.52 (m, 2H), 1.12 (s, 3H), 0.31-0.25 (m, 4H). ^{13}C-NMR (100 MHz, $CDCl_3$) δ 143.1, 128.4, 128.3, 125.6,

41.8, 33.5, 22.8, 15.5, 13.1. (EI) *m/z* (rel intens) 160 (M+, 98), 159 (46), 145 (89), 131 (45), 91 (100).

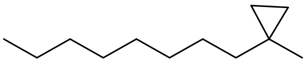

1-Methyl-1-octylcyclopropane (2b)

This product was prepared according to the above general procedure. Compound **2b** was obtained as transparent oil (0.9 g, 58%). ^{1}H-NMR (400 MHz, $CDCl_3$) δ 1.39-1.28 (m, 12H), 1.23-1.20 (m, 2H), 1.03 (s, 3H), 0.91 (t, J = 6.7 Hz, 3H), 0.26-0.20 (m, 4H). ^{3}C-NMR (100 MHz, $CDCl_3$) δ 39.4, 31.9, 30.0, 29.7, 29.4, 27.0, 22.8, 22.7, 15.3, 14.1, 12.9. (EI) *m/z* (rel intens) 168 (M+, 5), 138 (11), 111 (32), 97 (48), 83 (100).

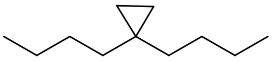

1,1-Dibutylcyclopropane (2c)

This product was prepared according to the above general procedure. Compound **2c** was obtained as transparent oil (0.4 g, 53%). ^{1}H-NMR (400 MHz, $CDCl_3$) δ 1.36-1.28 (m, 8H), 1.26-1.22 (m, 4H), 0.93 (t, J = 6.8 Hz, 6H), 0.22 (s, 4H). ^{13}C-NMR (100 MHz, $CDCl_3$) δ 36.0, 29.1, 23.3, 19.4, 14.4, 12.2. (EI) *m/z* (rel intens) 154 (M+, 55), 97 (100), 96 (54), 84 (44), 69 (99).

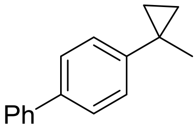

4-(1-Methylcyclopropyl)-1,1'-biphenyl (2f)

This product was prepared according to the above general procedure. Compound **2f** was obtained as white solid (1.2 g, 86%). ^{1}H-NMR (400 MHz, $CDCl_3$) δ 7.63-7.60 (m, 2H), 7.57-7.54 (m, 2H), 7.48-7.44 (m, 2H), 7.38-7.33 (m, 3H), 1.49 (s, 3H), 0.98-0.94 (m, 2H), 0.83-0.80 (m, 2H). ^{13}C-NMR (100 MHz, $CDCl_3$) δ 146.4, 141.3, 138.5, 128.9, 127.2, 127.2, 127.1, 25.8, 19.6, 16.1. (EI) *m/z* (rel intens) 208 (M+, 43), 193 (100), 179 (27), 178 (46), 165 (19).

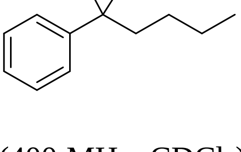

(1-Butylcyclopropyl)benzene (2j)

This product was prepared according to the above general procedure. Compound **2j** was obtained as transparent oil (0.9 g, 77%). ^{1}H-NMR (400 MHz, $CDCl_3$) δ 7.32-7.25 (m, 4H), 7.19-7.14 (m, 1H), 1.58-1.51 (m, 2H), 1.29-1.20 (m, 4H), 0.86-0.81 (m, 3H), 0.80-0.76 (m, 2H), 0.67-0.63 (m, 2H). ^{13}C-NMR (100 MHz, $CDCl_3$) δ 145.8, 129.2, 128.2, 125.9, 40.4, 29.6, 25.9, 23.1, 14.3, 13.2. (EI) *m/z* (rel intens) 174 (M+, 13), 117 (100), 116 (16), 115 (20), 91 (12).

General procedure for 1,3-difluorination and 1,3-oxyfluorination of cyclopropanes

Fluoroiodane reagent **1a** (56.0 mg, 0.2 mmol), the corresponding cyclopropane **2a-j** (0.1 mmol) and $AgBF_4$ (**3**) (19 mg, 0.1 mmol) were mixed in $CDCl_3$ (0.5 ml). This mixture was stirred at room temperature for 20 min – 24 h, unless otherwise stated. Products **4a-5f** were isolated by silica gel column chromatography.

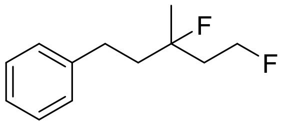

(3,5-Difluoro-3-methylpentyl)benzene (4a)

This product was prepared according to the above general procedure using fluoroiodine reagent **1a** (0.1 mmol). The reaction mixture was stirred at 50°C for 1h. Compound **4a** was isolated as a colorless oil using pentane:ether 50:1 as eluent system (14 mg, 71%). ^{1}H-NMR (400 MHz, $CDCl_3$) δ 7.33-7.29 (m, 2H), 7.23-7.20 (m, 3H), 4.74-4.70 (m, 1H), 4.62-4.58 (m, 1H), 2.75 (t, J = 8.8 Hz, 2H), 2.20-1.94 (m, 4H), 1.48 (d, J_{HF} = 22.1 Hz, 3H). ^{13}C-NMR (100 MHz, $CDCl_3$) δ 141.9, 128.7, 128.5, 126.2, 96.1 (dd, J_{CF} = 169.0, 3.3 Hz), 80.2 (dd, J_{CF} = 164.7, 6.8 Hz), 42.3 (dd, J_{CF} = 22.8, 0.8 Hz), 40.2 (dd, J_{CF} = 25.6, 19.4 Hz), 30.1 (d, J_{CF} = 5.7 Hz), 24.8 (dd, J_{CF} = 24.7, 1.3 Hz). ^{19}F-NMR (377 MHz, $CDCl_3$) δ -145.3 – -145.7 (m, 1F), -218.4 (tt, J_{HF} = 47.2, 26.3 Hz, 1F). HRMS (ESI): *m/z* calcd. for $[C_{12}H_{16}F_2+Na]^+$ 221.1112, found: 221.1121.

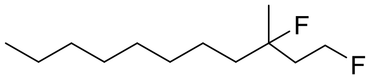

1,3-Difluoro-3-methylundecane (4b)

This product was prepared according to the above general procedure. The reaction mixture was stirred at room temperature for 4h. Compound **4b** was isolated as a colorless oil using pentane:ether 50:1 as eluent system (15 mg, 70%). ^{1}H-NMR (400 MHz, $CDCl_3$) δ 4.71-4.66 (m, 1H), 4.59-4.54 (m, 1H), 2.12-1.94 (m, 2H), 1.70-1.61 (m, 2H), 1.37 (d, J_{HF} = 22.1 Hz, 3H), 1.29-1.27 (m, 10H), 0.92-0.87 (m, 5H). ^{13}C-NMR (100 MHz, $CDCl_3$) δ 96.1 (dd, J_{CF} = 167.3, 3.7 Hz), 80.4 (dd, J_{CF} = 163.5, 6.8 Hz), 40.2 (dd, J_{CF} = 22.7, 0.5 Hz), 39.8 (dd, J_{CF} = 23.5, 19.5 Hz), 31.9, 29.9, 29.5, 29.2, 24.6 (dd, J_{CF} = 24.8, 1.1 Hz), 23.6 (d, J_{CF} = 5.6 Hz), 22.7, 14.1. ^{19}F-NMR (377 MHz, $CDCl_3$) δ -144.3 – -144.6 (m, 1F), -218.7 (tt, J_{HF} = 47.8, 25.3 Hz, 1F). HRMS (ESI): *m/z* calcd. for $[C_{12}H_{24}F_2+Na]^+$ 229.1738, found: 229.1731.

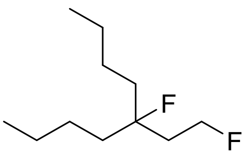

5-Fluoro-5-(2-fluoroethyl)nonane (4c)

This product was prepared according to the above general procedure. The reaction mixture was stirred at room temperature for 4h. Compound **4c** was isolated as a colorless oil using pentane:ether 50:1 as eluent system (10 mg, 51%). ^{1}H-NMR (400 MHz, $CDCl_3$) δ 4.60 (dt, J_{HF} = 47.3, 6.1 Hz, 2H), 2.09-1.95 (m, 2H), 1.67-1.58 (m, 4H), 1.33-1.25 (m, 8H), 0.91 (t, J = 6.8 Hz, 6H). ^{13}C-NMR (100 MHz, $CDCl_3$) δ 98.0 (dd, J_{CF} = 169.3, 4.5 Hz), 80.4 (dd, J_{CF} = 164.1, 6.7 Hz), 37.7 (dd, J_{CF} = 26.0, 19.1 Hz), 37.2 (dd, J_{CF} = 22.7, 0.5 Hz), 25.7 (d, J_{CF} = 6.0 Hz), 23.2, 14.2. ^{19}F-NMR (377 MHz, $CDCl_3$) δ -150.2– -150.5 (m, 1F), -219.1 (tt, J_{HF} = 47.3, 24.3 Hz, 1F). HRMS (ESI): *m/z* calcd. for $[C_{11}H_{22}F_2+Na]^+$ 215.1582, found: 215.1581.

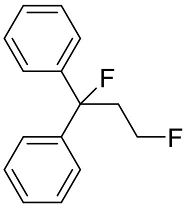

(1,3-Difluoropropane-1,1-diyl)dibenzene (4d)

This product was prepared according to the above general procedure. The reaction mixture was stirred at room temperature for 2h. Compound **4d** was isolated as a colorless oil using pentane:ether 50:1 as eluent system (11 mg, 47%). ^{1}H-NMR (400 MHz, $CDCl_3$) δ 7.39-7.27 (m, 10H), 4.55 (dt, J_{HF} = 46.7, 7.1 Hz, 2H), 2.93-2.80 (m, 2H). ^{13}C-NMR (100 MHz, $CDCl_3$) δ 142.7 (d, J_{CF} = 23.3 Hz), 128.4 (d, J_{CF} = 0.6 Hz), 127.9 (d, J_{CF} = 1.6 Hz), 125.1 (d, J_{CF} = 8.2 Hz), 97.8 (dd, J_{CF} = 176.5, 9.7 Hz), 80.2 (dd, J_{CF} = 164.5, 4.6 Hz), 40.3 (dd, J_{CF} = 24.4, 20.5 Hz). ^{19}F-NMR (377 MHz, $CDCl_3$) δ -148.7 (t, J_{HF} = 24.2 Hz, 1F), -221.5 (ttd, J_{HF} = 46.6, 16.5 Hz, J_{FF} = 1.6 Hz, 1F). HRMS (ESI): *m/z* calcd. for $[C_{15}H_{14}F_2+Na]^+$ 255.0956, found: 255.0964.

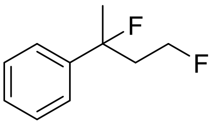

(2,4-Difluorobutan-2-yl)benzene (4e)

This product was prepared according to the above general procedure using fluoroiodine reagent **1a** (0.1 mmol). The reaction mixture was stirred at room temperature for 6h. Compound **4e** was isolated as a colorless oil using pentane:ether 50:1 as eluent system (10 mg, 59%). ^{1}H-NMR (400 MHz, $CDCl_3$) δ 7.42-7.30 (m, 5H), 4.60 (ddt, J_{HF} = 47.3, J = 9.5, 6.5 Hz, 1H), 4.41 (ddt, J_{HF} = 47.0, J = 9.5, 6.3 Hz, 1H), 2.36-2.30 (m, 2H), 1.75 (dd, J_{HF}= 22.7, 0.6 Hz, 3H). ^{13}C-NMR (100 MHz, $CDCl_3$) δ 143.9 (d, J_{CF} = 21.8 Hz), 128.6 (d, J_{CF} = 1.5 Hz), 127.6 (d, J_{CF} = 1.1 Hz), 123.9 (d, J_{CF} = 9.7 Hz), 96.3 (dd, J_{CF} = 172.6, 6.2 Hz), 80.0 (dd, J_{CF} = 164.5, 5.3 Hz), 42.4 (dd, J_{CF} = 22.4, 20.0 Hz), 27.8 (dd, J_{CF} = 25.1, 1.5 Hz). ^{19}F-NMR

(377 MHz, $CDCl_3$) δ -147.9 – -148.2 (m, 1F), -220.1 (tt, J_{HF} = 46.2, 22.2 Hz, 1F). HRMS (ESI): *m/z* calcd. for $[C_{10}H_{12}F_2+Na]^+$ 193.0799, found: 193.0798.

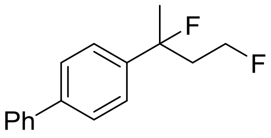

4-(2,4-Difluorobutan-2-yl)-1,1'-biphenyl (4f)

This product was prepared according to the above general procedure. The reaction mixture was stirred at room temperature for 1h. Compound **4f** was isolated as a colorless oil using pentane:ether 50:1 as eluent system (13 mg, 55%). ^{1}H-NMR (400 MHz, $CDCl_3$) δ 7.63-7.57 (m, 4H), 7.47-7.31 (m, 5H), 4.63 (ddt, J_{HF} = 47.5, J = 9.0, 6.5 Hz, 1H), 4.44 (ddt, J_{HF} = 46.7, J = 9.6, 6.3 Hz, 1H), 2.50-2.34 (m, 2H), 1.77 (dd, J_{HF}= 22.7, 0.8 Hz, 3H). ^{13}C-NMR (100 MHz, $CDCl_3$) δ 143.0 (d, J_{CF} = 22.1 Hz), 140.7, 140.5 (d, J_{CF} = 1.3 Hz), 128.8, 127.5 (d, J_{CF} = 1.2 Hz), 127.2, 127.1, 124.4 (d, J_{CF} = 9.4 Hz), 96.2 (dd, J_{CF} = 172.1, 5.7 Hz), 80.0 (dd, J_{CF} = 164.4, 5.3 Hz), 42.2 (dd, J_{CF} = 23.8, 19.2 Hz), 27.9 (dd, J_{CF} = 25.0, 1.2 Hz). ^{19}F-NMR (377 MHz, $CDCl_3$) δ -147.4 – -147.7 (m, 1F), -220.0 (tt, J_{HF} = 46.3, 22.5 Hz, 1F). HRMS (ESI): *m/z* calcd. for $[C_{16}H_{16}F_2+Na]^+$ 269.1112, found: 269.1118.

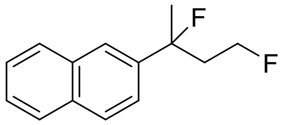

2-(2,4-Difluorobutan-2-yl)naphthalene (4g)

This product was prepared according to the above general procedure. The reaction mixture was stirred at room temperature for 3h. Compound **4g** was isolated as a colorless oil using pentane:ether 100:1 as eluent system (16 mg, 65%). ^{1}H-NMR (400 MHz, $CDCl_3$) δ 7.88-7.81 (m, 4H), 7.54-7.39 (m, 3H), 4.65 (ddt, J_{HF} = 47.1, J = 9.2, 6.2 Hz, 1H), 4.43 (ddt, J_{HF} = 46.8, J = 9.2, 6.2 Hz, 1H), 2.56-2-38 (m, 2H), 1.82 (dd, J_{HF}= 22.6, 0.8 Hz, 3H). ^{13}C-NMR (100 MHz, $CDCl_3$) δ 141.2 (d, J_{CF} = 21.7 Hz), 133.0 (d, J_{CF} = 1.4 Hz), 132.6 (d, J_{CF} = 0.9 Hz), 128.3 (d, J_{CF} = 1.5 Hz), 128.2, 127.6, 126.4, 126.2, 122.7 (d, J_{CF} = 10.8 Hz), 122.2 (d, J_{CF} = 8.4 Hz), 96.4 (dd, J_{CF} = 172.9, 6.1 Hz), 80.2 (dd, J_{CF} = 164.5, 5.2 Hz), 42.3 (dd, J_{CF} = 23.5, 19.5 Hz), 27.9 (dd, J_{CF} = 24.8, 1.4 Hz). ^{19}F-NMR (377 MHz, $CDCl_3$) δ -147.5 – -147.6 (m, 1F), -220.0 (tt, J_{HF} = 45.0, 21.8 Hz, 1F) HRMS (ESI): *m/z* calcd. for $[C_{14}H_{14}F_2+Na]^+$ 243.0956, found: 243.0946.

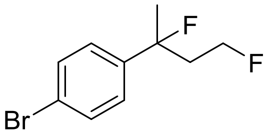

1-Bromo-4-(2,4-difluorobutan-2-yl)benzene (4h)

This product was prepared according to the above general procedure. The reaction mixture was stirred at room temperature for 24h. Compound **4h** was isolated as a colorless oil using pentane:ether 50:1 as eluent system (12 mg, 57%). ^{1}H-NMR (400 MHz, $CDCl_3$) δ 7.54-7.51 (m, 2H), 7.23-7.22 (m, 2H), 4.57 (ddt,

J_{HF} = 47.1, J = 9.7, 6.2 Hz, 1H), 4.39 (ddt, J_{HF} = 47.1, J = 9.7, 5.8 Hz, 1H), 2.46-2.23 (m, 2H), 1.70 (dd, J_{HF}= 22.7, 0.7 Hz, 3H). ^{13}C-NMR (100 MHz, $CDCl_3$) δ 143.1 (d, J_{CF} = 22.4 Hz), 131.6 (d, J_{CF} = 1.3 Hz), 125.7 (d, J_{CF} = 9.7 Hz), 121.6 (d, J_{CF} = 1.7 Hz), 96.0 (dd, J_{CF} = 174.1, 5.8 Hz), 79.9 (dd, J_{CF} = 165.1, 5.3 Hz), 42.2 (dd, J_{CF} = 21.8, 20.0 Hz), 27.7 (dd, J_{CF} = 25.1, 1.4 Hz). ^{19}F-NMR (377 MHz, $CDCl_3$) δ -148.1 – -148.3 (m, 1F), -219.9 (tt, J_{HF} = 46.2, 22.7 Hz, 1F). HRMS (ESI): *m*/*z* calcd. for $[C_{10}H_{11}BrF_2+Na]^+$ 270.9904, found: 270.9917.

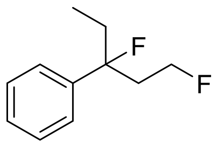

(1,3-Difluoropentan-3-yl)benzene (4i)

This product was prepared according to the above general procedure. The reaction mixture was stirred at room temperature for 24h. Compound **4i** was isolated as a colorless oil using pentane:ether 50:1 as eluent system (15 mg, 70%). ^{1}H-NMR (400 MHz, $CDCl_3$) δ 7.39-7.35 (m, 2H), 7.30-7.27 (m, 3H), 4.62-4.45 (m, 1H), 4.42-4.24 (m, 1H), 2.43-2.22 (m, 2H), 2.10-1.88 (m, 2H), 0.78 (t, J = 7.4 Hz, 3H). ^{13}C-NMR (100 MHz, $CDCl_3$) δ 141.9 (d, J_{CF} = 21.8 Hz), 128.3 (d, J_{CF} = 1.8 Hz), 127.3 (d, J_{CF} = 1.0 Hz), 124.3 (d, J_{CF} = 10.3 Hz), 98.5 (dd, J_{CF} = 176.5, 7.2 Hz), 80.1 (dd, J_{CF} = 163.8, 5.3 Hz), 40.8 (dd, J_{CF} = 24.8, 19.8 Hz), 33.7 (dd, J_{CF} = 23.8, 0.8 Hz), 7.4 (d, J_{CF} = 5.0 Hz). ^{19}F-NMR (377 MHz, $CDCl_3$) δ -162.3 – -162.5 (m, 1F), -220.4 (tt, J_{HF} = 46.5, 21.4 Hz, 1F). HRMS (ESI): *m*/*z* calcd. for $[C_{11}H_{14}F_2+Na]^+$ 207.0956, found: 207.0955.

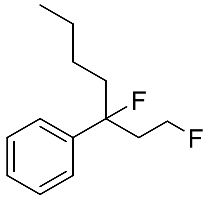

(1,3-Difluoroheptan-3-yl)benzene (4j)

This product was prepared according to the above general procedure. The reaction mixture was stirred at room temperature for 24h. Compound **4j** was isolated as a colorless oil using pentane:ether 50:1 as eluent system (14 mg, 66%). ^{1}H-NMR (400 MHz, $CDCl_3$) δ 7.41-7.37 (m, 2H), 7.37-7.28 (m, 3H), 4.64-4.47 (m, 1H), 4.42-4.26 (m, 1H), 2.47-2.26 (m, 2H), 2.04-1.86 (m, 2H), 1.34-1.24 (m, 4H), 0.85 (t, J = 7.3 Hz, 3H). ^{13}C-NMR (100 MHz, $CDCl_3$) δ 142.2 (d, J_{CF} = 22.1 Hz), 128.3 (d, J_{CF} = 1.7 Hz), 127.3 (d, J_{CF} = 1.1 Hz), 124.3 (d, J_{CF} = 10.6 Hz), 98.3 (dd, J_{CF} = 174.8, 7.3 Hz), 80.2 (dd, J_{CF} = 164.8, 5.2 Hz), 41.3 (dd, J_{CF} = 20.0, 19.6 Hz), 40.7 (dd, J_{CF} = 23.2, 0.7 Hz), 25.2 (d, J_{CF} = 3.7 Hz), 22.8, 13.9. ^{19}F-NMR (377 MHz, $CDCl_3$) δ -160.2 – -160.4 (m, 1F), -220.4 (tt, J_{HF} = 47.2, 21.5 Hz, 1F). HRMS (ESI): *m*/*z* calcd. for $[C_{13}H_{18}F_2+Na]^+$ 235.1269, found: 235.1277.

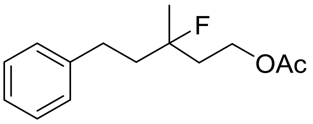

3-Fluoro-3-methyl-5-phenylpentyl acetate (5a)

This product was prepared according to the above general procedure using hypervalent iodine reagents **1b** (64 mg, 0.2 mmol) or **1c** (32 mg, 0.1 mmol) instead of **1a**. The reaction mixtures were stirred at room temperature for 4h with **1b** and for 20 minutes with **1c**. Compound **5a** was isolated as a colorless oil using pentane:ether 20:1 as eluent system (20 mg, 84%). ^{1}H-NMR (400 MHz, $CDCl_3$) δ 7.32-7.27 (m, 2H), 7.24-7.21 (m, 3H), 4.24 (t, J = 7.3 Hz, 2H), 2.72 (t, J = 8.8 Hz, 2H), 2.05 (s, 3H), 2.04-1.91 (m, 4H), 1.43 (d, J_{HF} = 21.8 Hz, 3H). ^{13}C-NMR (100 MHz, $CDCl_3$) δ 171.0, 141.6, 128.5, 128.3, 126.0, 95.9 (d, J_{CF} = 169.3 Hz), 60.3 (d, J_{CF} = 7.1 Hz), 41.9 (d, J_{CF} = 22.8 Hz), 38.0 (d, J_{CF} = 23.1 Hz), 29.9 (d, J_{CF} = 5.9 Hz), 24.6 (d, J_{CF} = 24.9 Hz), 21.0. ^{19}F-NMR (377 MHz, $CDCl_3$) δ -145.4– -145.8 (m). HRMS (ESI): *m/z* calcd. for $[C_{14}H_{19}O_2F+Na]^+$ 261.1261, found: 261.1255.

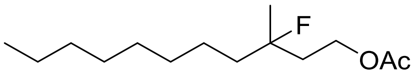

3-Fluoro-3-methylundecyl acetate (5b)

This product was prepared according to the above general procedure using reagent **1b** (32 mg, 0.1 mmol) instead of **1a**. The reaction mixture was stirred at room temperature for 2h. Compound **5b** was isolated as a colorless oil using pentane:ether 20:1 as eluent system (12 mg, 50%). ^{1}H-NMR (400 MHz, $CDCl_3$) δ 4.19 (dt, J = 7.2, J_{HF} = 1.9 Hz, 2H), 2.05 (s, 3H), 2.05-1.84 (m, 2H), 1.61-1.55 (m, 2H), 1.33 (d, J_{HF} = 22.1 Hz, 3H), 1.30-1.20 (m, 12H), 0.87 (t, J = 6.6 Hz, 3H). ^{13}C-NMR (100 MHz, $CDCl_3$) δ 171.4, 96.5 (d, J_{CF} = 167.3 Hz), 60.6 (d, J_{CF} = 6.2 Hz), 40.1 (d, J_{CF} = 22.5 Hz), 37.8 (d, J_{CF} = 23.3 Hz), 32.0, 30.1, 29.7, 29.5, 24.7 (d, J_{CF} = 24.8 Hz), 23.8 (d, J_{CF} = 5.8 Hz), 22.9, 21.2, 14.3. ^{19}F-NMR (377 MHz, $CDCl_3$) δ -143.9– -144.4 (m). HRMS (ESI): *m/z* calcd. for $[C_{14}H_{27}O_2F+Na]^+$ 269.1887, found: 269.1890.

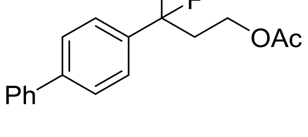

3-([1,1'-Biphenyl]-4-yl)-3-fluorobutyl acetate (5c)

This product was prepared according to the above general procedure using **1b** (32 mg, 0.1 mmol) instead of **1a**. The reaction mixture was stirred at room temperature for 2h. Compound **5c** was isolated as a colorless oil using pentane:ether 20:1 as eluent system (15 mg, 51 %). ^{1}H-NMR (400 MHz, $CDCl_3$) δ 7.64-7.54 (m, 4H), 7.49-7.33 (m, 5H), 4.20-4.00 (m, 2H), 2.42-2.25 (m, 2H), 1.93 (s, 3H), 1.73 (d, J_{HF} = 22.5 Hz, 3H). ^{13}C-NMR (100 MHz, $CDCl_3$) δ 171.3, 142.8 (d, J_{CF} = 22.0 Hz), 140.7, 140.4 (d, J_{CF} = 1.1 Hz), 129.0, 127.6, 127.3 (d, J_{CF} = 1.6 Hz), 127.2, 124.5 (d, J_{CF} = 9.7

Hz), 97.0 (d, J_{CF} = 173.9 Hz), 60.4 (d, J_{CF} = 4.9 Hz), 40.4 (d, J_{CF} = 23.8 Hz), 28.5 (d, J_{CF} = 25.7 Hz), 21.1. ^{19}F-NMR (377 MHz, $CDCl_3$) δ -149.6– -149.9 (m). HRMS (ESI): *m/z* calcd. for $[C_{18}H_{19}O_2F+Na]^+$ 309.1261, found: 309.1275.

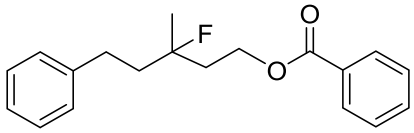

3-Fluoro-3-methyl-5-phenylpentyl benzoate (5d)

This product was prepared according to the above general procedure using hypervalent iodine reagent **1d** (45 mg, 0.1 mmol) instead of **1a**. The reaction mixture was stirred at room temperature for 20 minutes. Compound **5d** was isolated as a colorless oil using pentane:ether 50:1 as eluent system (27 mg, 80%). ^{1}H-NMR (400 MHz, $CDCl_3$) δ 8.04-8.01 (m, 2H), 7.58-7.54 (m, 1H), 7.45-7.41 (m, 2H), 7.30-7.27 (m, 2H), 7.21-7.18 (m, 3H), 4.51 (dt, *J* = 6.6, J_{HF} = 0.7 Hz, 2H), 2.76 (t, *J* = 8.9 Hz, 2H), 2.28-1.92 (m, 4H), 1.49 (d, J_{HF} = 21.8 Hz, 3H). ^{13}C-NMR (100 MHz, $CDCl_3$) δ 166.7, 141.8, 133.2, 130.3, 129.7, 128.7, 128.6, 128.5, 126.2, 95.8 (d, J_{CF} = 168.9 Hz), 61.0 (d, J_{CF} = 7.0 Hz), 42.2 (d, J_{CF} = 22.7 Hz), 38.3 (d, J_{CF} = 23.4 Hz), 30.1 (d, J_{CF} = 5.9 Hz), 24.7 (d, J_{CF} = 24.8 Hz). ^{19}F-NMR (377 MHz, $CDCl_3$) δ -145.3– -145.6 (m). HRMS (ESI): *m/z* calcd. for $[C_{19}H_{21}O_2F+Na]^+$ 323.1418, found: 323.1418.

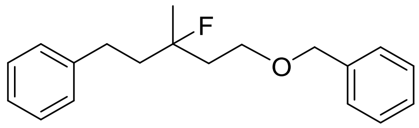

(5-(Benzyloxy)-3-fluoro-3-methylpentyl)benzene (5e)

This product was prepared according to the above general procedure using fluoroiodine reagent **1a** (0.1 mmol) and benzyl alcohol **6** (32 mg, 0.3 mmol). The reaction mixture was stirred at room temperature for 18h. Compound **5e** was isolated as a colorless oil using pentane:ether 20:1 as eluent system (23 mg, 80 %). ^{1}H-NMR (400 MHz, $CDCl_3$) δ 7.34-7.25 (m, 7H), 7.20-7.15 (m, 3H), 4.51 (s, 2H), 3.64 (t, *J* = 6.7 Hz, 2H), 2.71 (t, *J* = 8.3 Hz, 2H), 2.09-1.89 (m, 4H), 1.42 (d, J_{HF} = 22.0 Hz, 3H). ^{13}C-NMR (100 MHz, $CDCl_3$) δ 142.0, 138.3, 128.5, 128.4, 128.3, 127.7, 127.6, 125.9, 96.2 (d, J_{CF} = 168.2 Hz), 73.2, 66.1 (d, J_{CF} = 7.1 Hz), 42.2 (d, J_{CF} = 24.0 Hz), 39.3 (d, J_{CF} = 22.9 Hz), 29.9 (d, J_{CF} = 5.7 Hz), 24.6 (d, J_{CF} = 24.7 Hz). ^{19}F-NMR (377 MHz, $CDCl_3$) δ -143.7– -144.1 (m). HRMS (ESI): *m/z* calcd. for $[C_{19}H_{23}OF+Na]^+$ 309.1625, found: 309.1612.

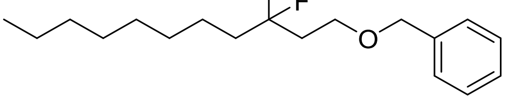

(((3-Fluoro-3-methylundecyl)oxy)methyl)benzene (5f)

This product was prepared according to the above general procedure using fluoroiodine reagent **1a** (0.1 mmol) and benzyl alcohol **6** (32 mg, 0.3 mmol). The reaction mixture was

stirred at room temperature for 18h. Compound **5f** was isolated as a colorless oil using pentane:ether 20:1 as eluent system (27 mg, 91 %). ^{1}H-NMR (400 MHz, $CDCl_3$) δ 7.37-7.27 (m, 5H), 4.53 (s, 2H), 3.63-3.59 (m, 2H), 2.02-1.91 (m, 2H), 1.65-1.56 (m, 2H), 1.33 (d, J_{HF} = 21.9 Hz, 3H), 1.30-1.20 (m, 12H), 0.89 (t, J = 7.0 Hz, 3H). ^{13}C-NMR (100 MHz, $CDCl_3$) δ 138.4, 128.4, 127.8, 127.6, 96.7 (d, J_{CF} = 166.9 Hz), 73.1, 66.1 (d, J_{CF} = 6.6 Hz), 40.2 (d, J_{CF} = 22.8 Hz), 39.2 (d, J_{CF} = 23.1 Hz), 31.9, 30.0, 29.5, 29.3, 24.7 (d, J_{CF} = 25.0 Hz), 23.7 (d, J_{CF} = 5.7 Hz), 22.7, 14.1. ^{19}F-NMR (377 MHz, $CDCl_3$) δ -142.8– -143.2 (m). HRMS (ESI): *m/z* calcd. for $[C_{19}H_{31}OF+Na]^+$ 317.2251, found: 317.2242.

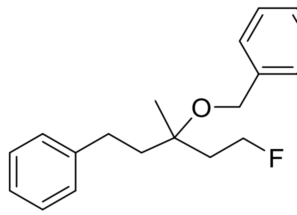

(3-(benzyloxy)-5-fluoro-3-methylpentyl)benzene (5g)

This product was prepared according to the above general procedure using fluoroiodine reagent **1a** (0.1 mmol). The reaction mixture was stirred at 50°C for 1h. Benzyl alcohol **6** (32 mg, 0.3 mmol) was added to the reaction mixture after formation of **4a** was confirmed according to the crude NMR. Then, this reaction mixture was stirred at room temperature for additional 18h. Compound **5g** was isolated as a colorless oil using pentane:ether 50:1 as eluent system (11 mg, 51 %). ^{1}H-NMR (400 MHz, $CDCl_3$) δ 7.37-7.32 (m, 4H), 7.31-7.26 (m, 3H), 7.22-7.16 (m, 3H), 4.75-4.69 (m, 1H), 4.64-4.58 (m, 1H), 4.46 (s, 2H), 2.72-2.68 (m, 2H), 2.19-2.00 (m, 2H), 1.94-1.90 (m, 2H), 1.36 (s, 3H). ^{13}C-NMR (100 MHz, $CDCl_3$) δ 142.6, 139.4, 128.6, 128.6, 128.5, 127.5, 127.4, 126.0, 81.1 (d, J_{CF} = 162.6 Hz), 76.0 (d, J_{CF} = 5.1 Hz), 63.6, 40.9, 38.9 (d, J_{CF} = 18.6 Hz), 30.3, 23.8.^{19}F-NMR (377 MHz, $CDCl_3$) δ -218.4 (tdd, J_{HF} = 47.3, 25.0, 23.0 Hz, 1F). HRMS (ESI): *m/z* calcd. for $[C_{19}H_{23}OF+Na]^+$ 309.1625, found: 309.1637.

References

1. (a) G. C. Geary, E. G. Hope, K. Singh and A. M. Stuart, Chem. Commun., 2013, **49**, 9263; (b) V. Matoušek, E. Pietrasiak, R. Schwenk and A. Togni, J. Org. Chem., 2013, **78**, 6763.
2. M. V. Vita, P. Caramenti and Waser, J. Org. Lett., 2015, **17,** 5832.
3. S. A. Moteki, A. Usui, S. Selvakumar, T. Zhang and K. Maruoka, Angew. Chem. Int. Ed., 2014, **53**, 11060.
4. D. H. T. Phan, K. G. M. Kou and V. M. Dong, J. Am. Chem. Soc. 2010, **132**, 16354.
5. A. B. Charette, S. Francoeur, J. Martel and N. Wilb, Angew. Chem. Int. Ed. 2000, **39**, 4539.
6. a) C.-Y. Huang and A. G. Doyle, J. Am. Chem. Soc. 2015, **137**, 5638. b) G. Pratsch and L. E. Overman , J. Org. Chem. 2015, **80**, 11388. c) E. Emer, L. Pfeifer, J. M. Brown and V. Gouverneur, Angew. Chem. Int. Ed. 2014, **53**, 4181.

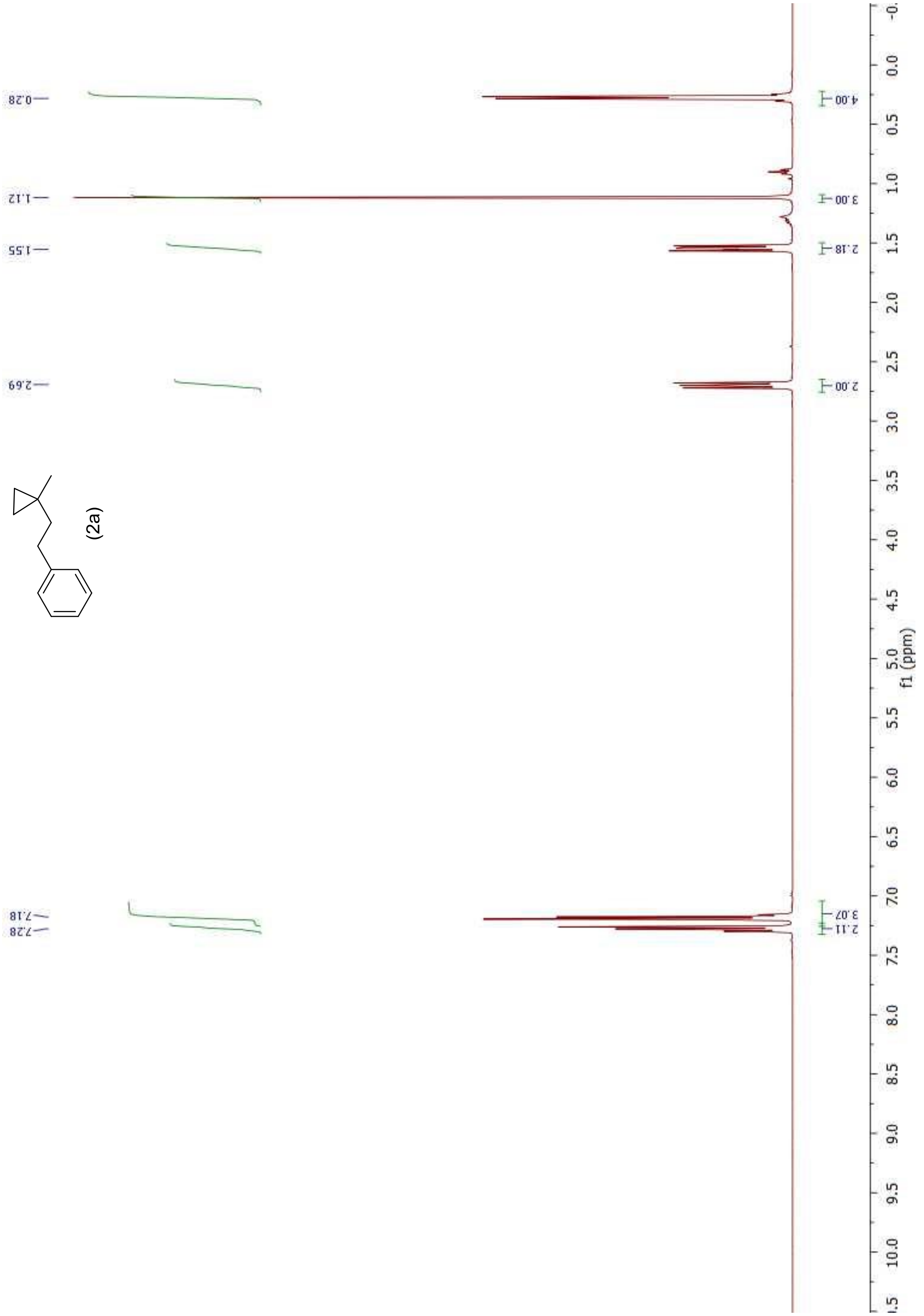
(2a)
0.28
1.12
1.55
2.69
7.18
7.28
4.00
3.00
2.18
2.00
3.07
2.11
f1 (ppm)

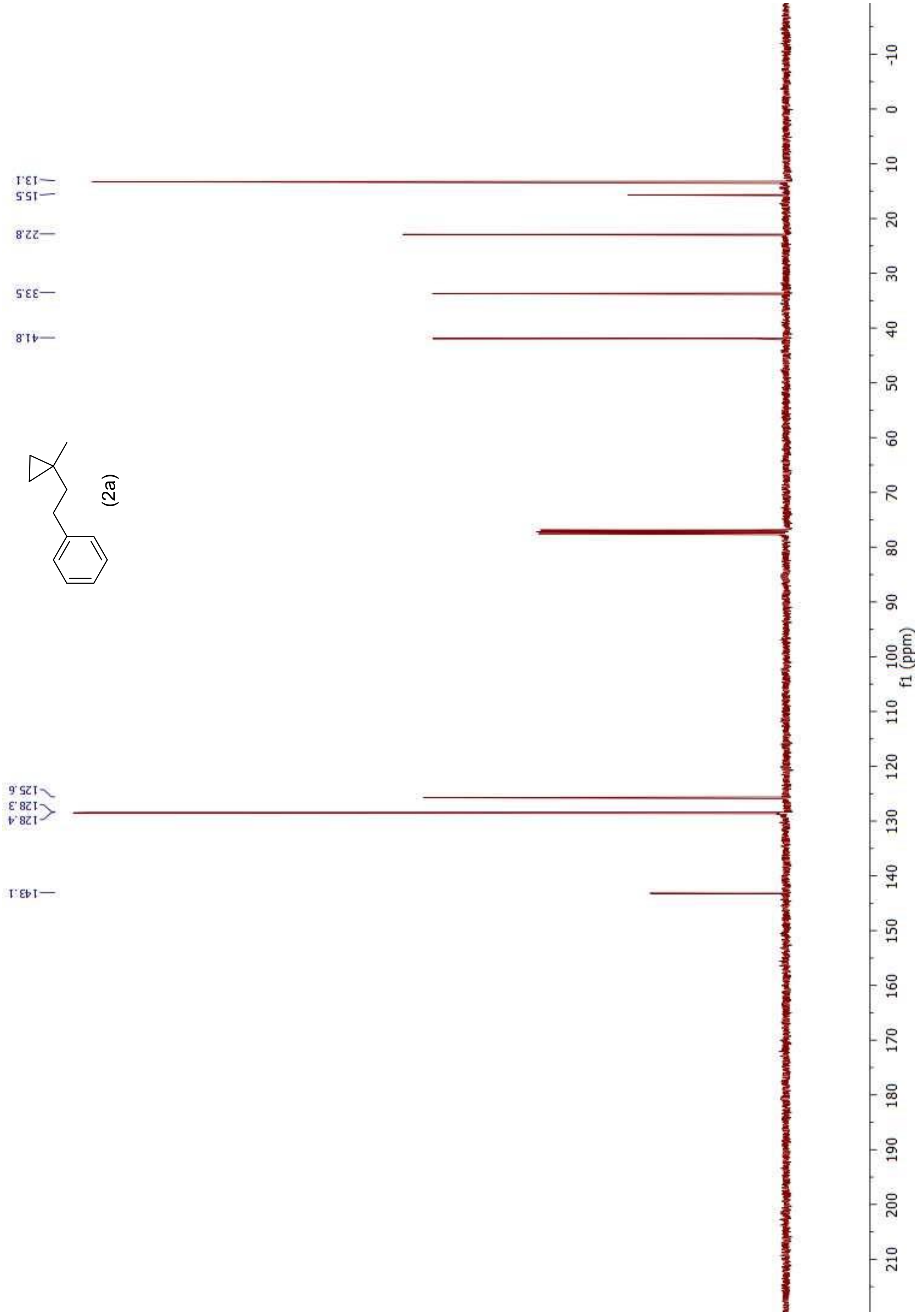
13.1
15.5
22.8
33.5
41.8
125.6
128.3
128.4
143.1
(2a)
f1 (ppm)

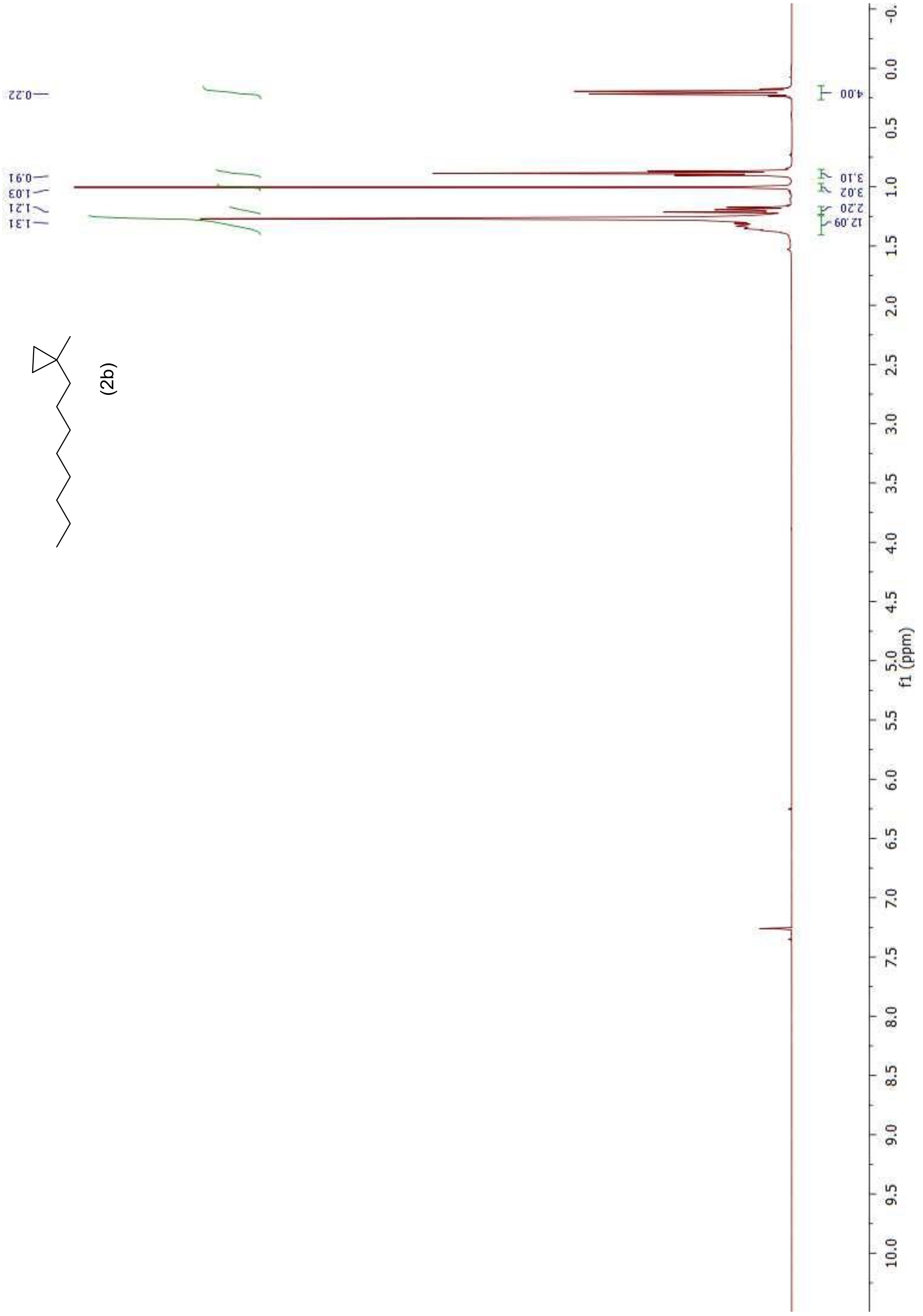
(2b)
0.22
0.91
1.03
1.21
1.31
4.00
3.10
3.02
2.20
12.09
f1 (ppm)

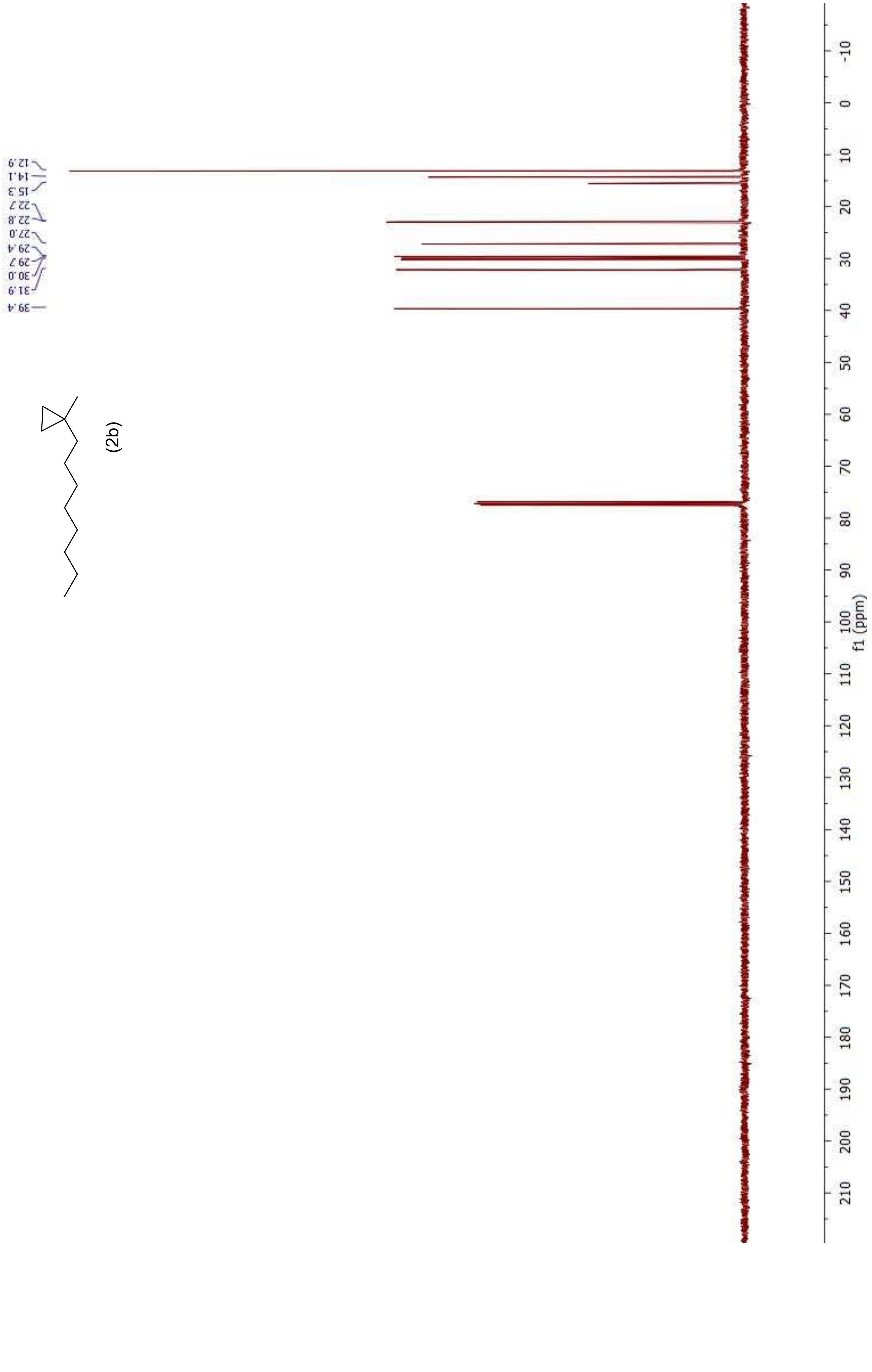

(2b)

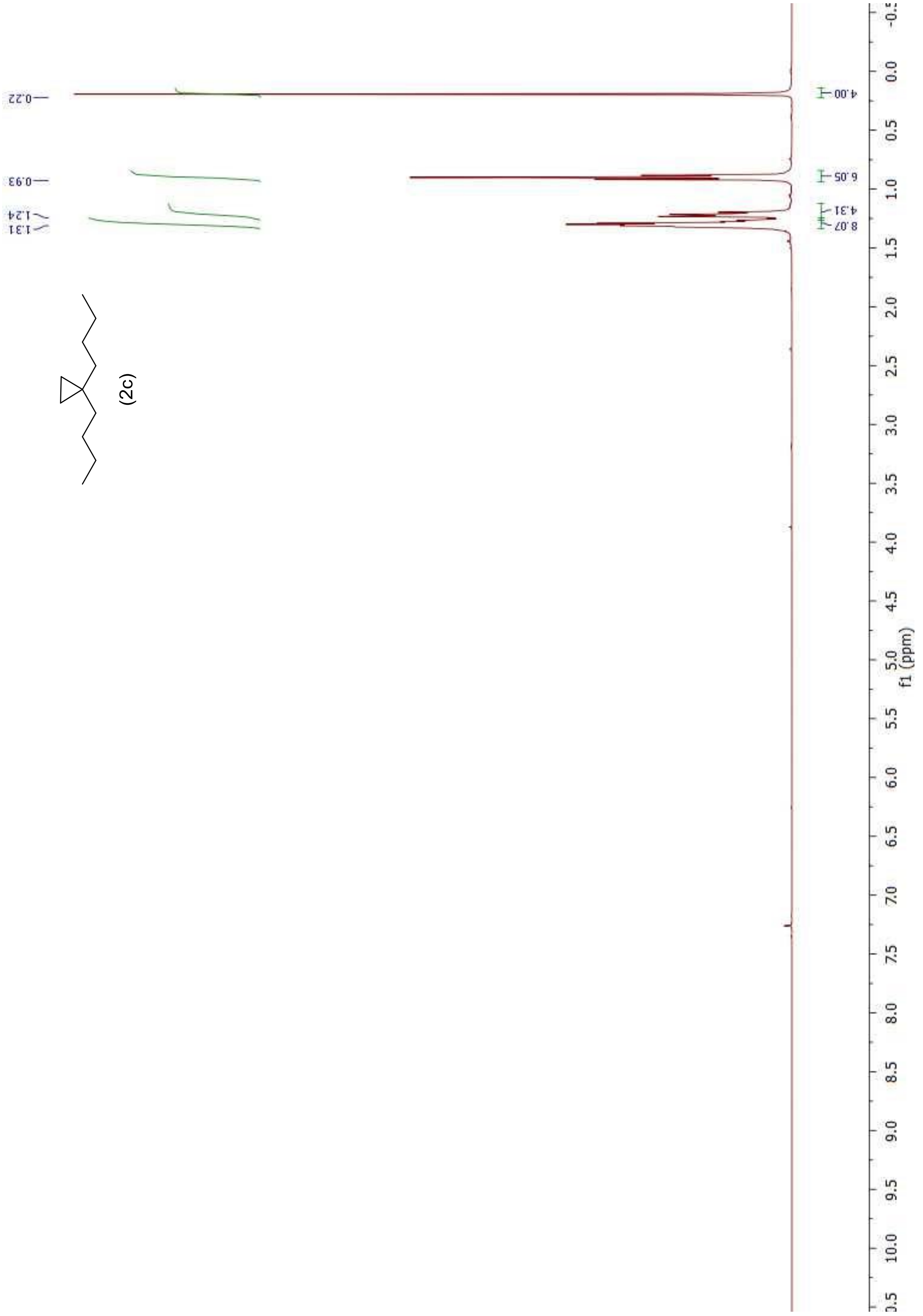
0.22
0.93
1.24
1.31
4.00
6.05
4.31
8.07
(2c)
f1 (ppm)

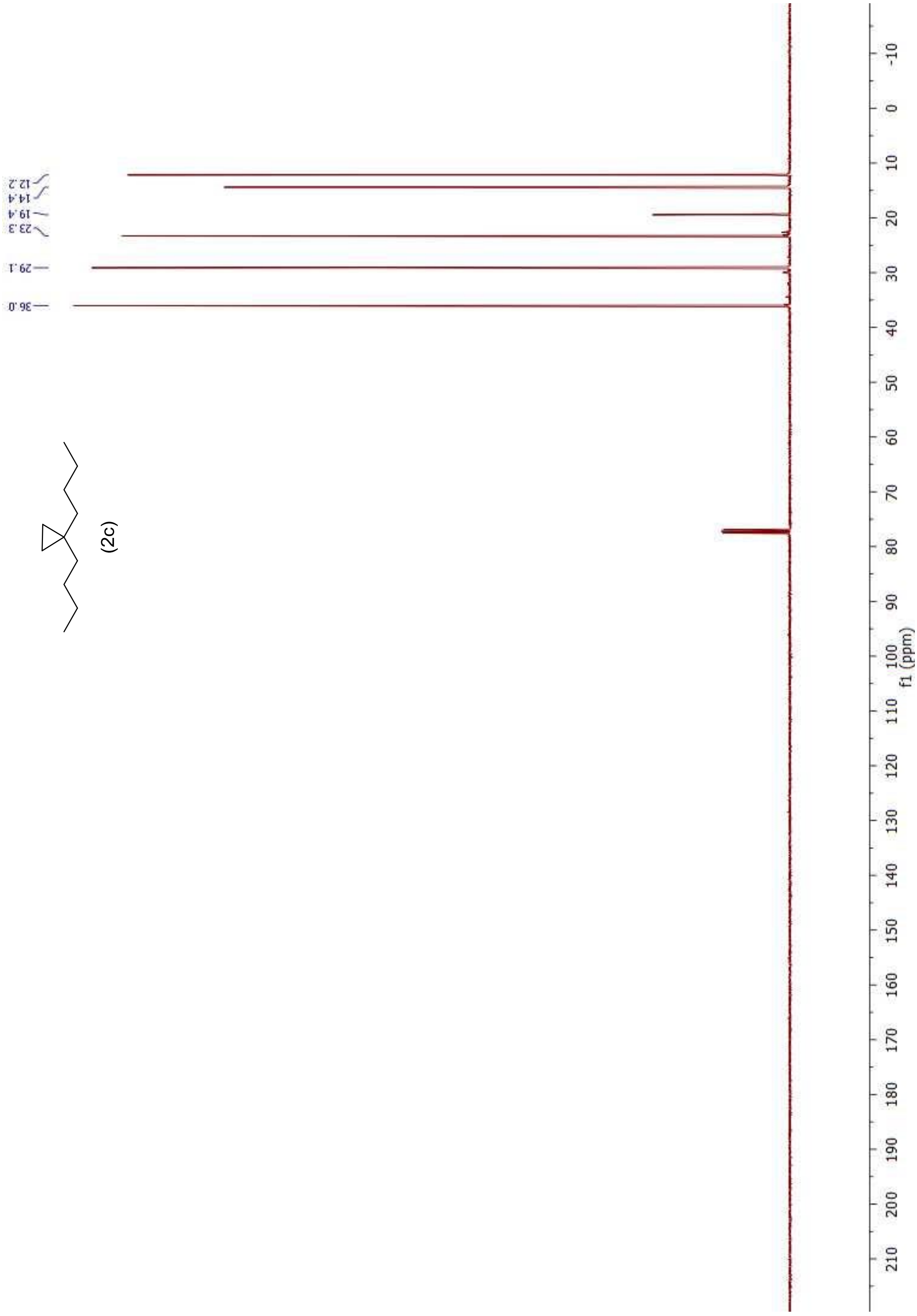
12.2
14.4
19.4
23.3
29.1
36.0
(2c)
f1 (ppm)

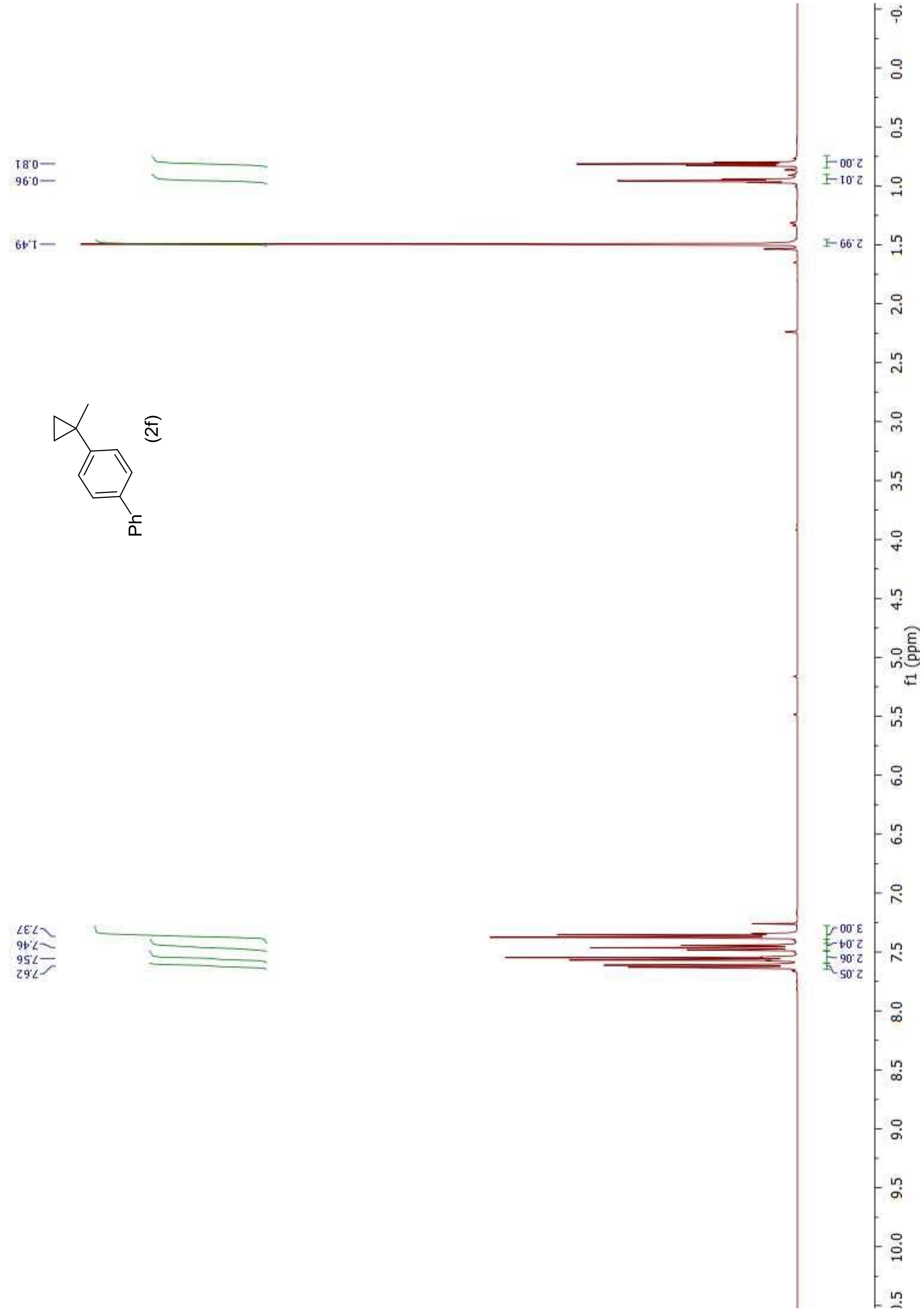
0.81
0.96
1.49
7.37
7.46
7.56
7.62
2.00
2.01
2.99
3.00
2.04
2.06
2.05
Ph
(2f)
f1 (ppm)

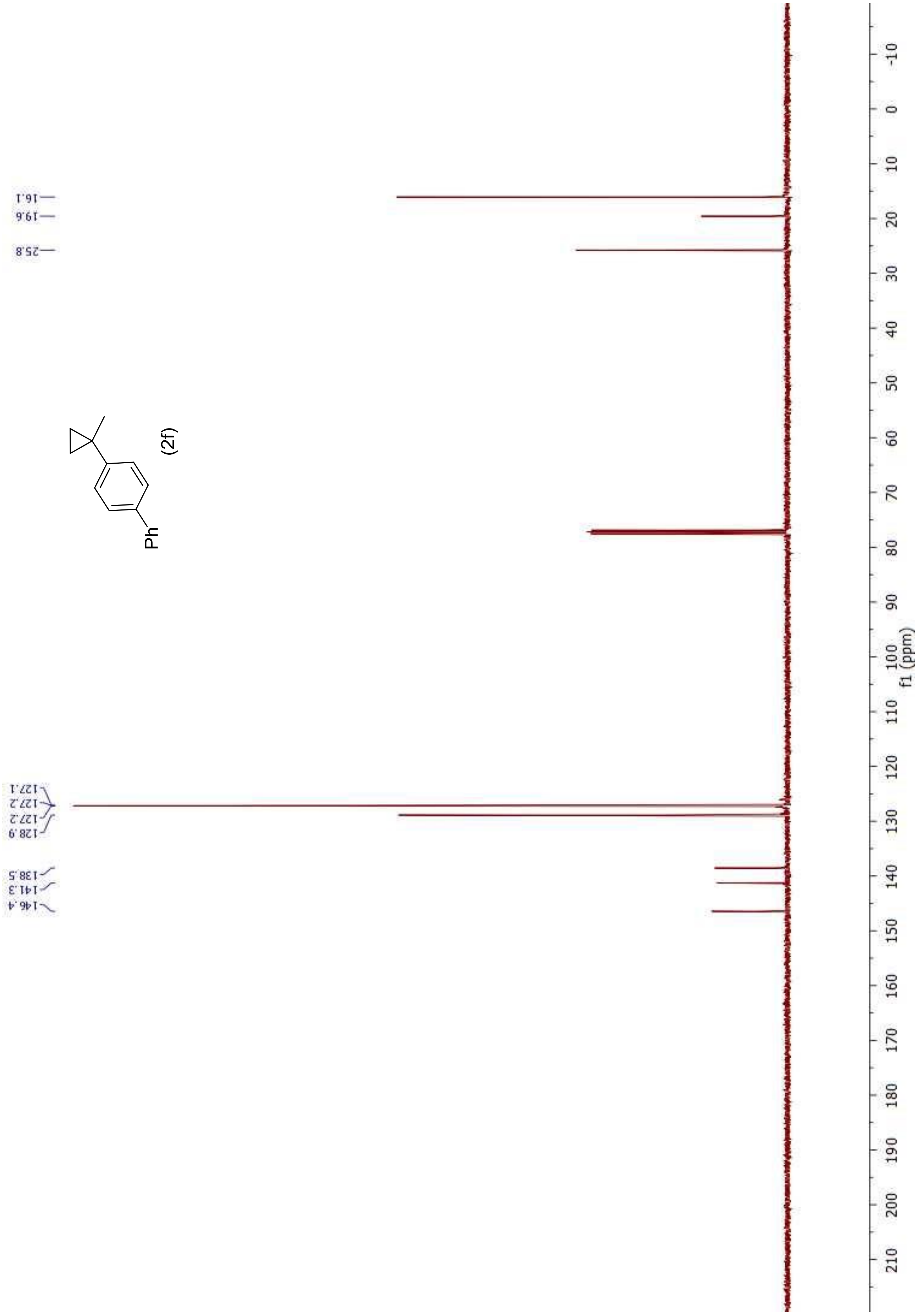
16.1
19.6
25.8
Ph
(2f)
127.1
127.2
127.2
128.9
138.5
141.3
146.4
f1 (ppm)

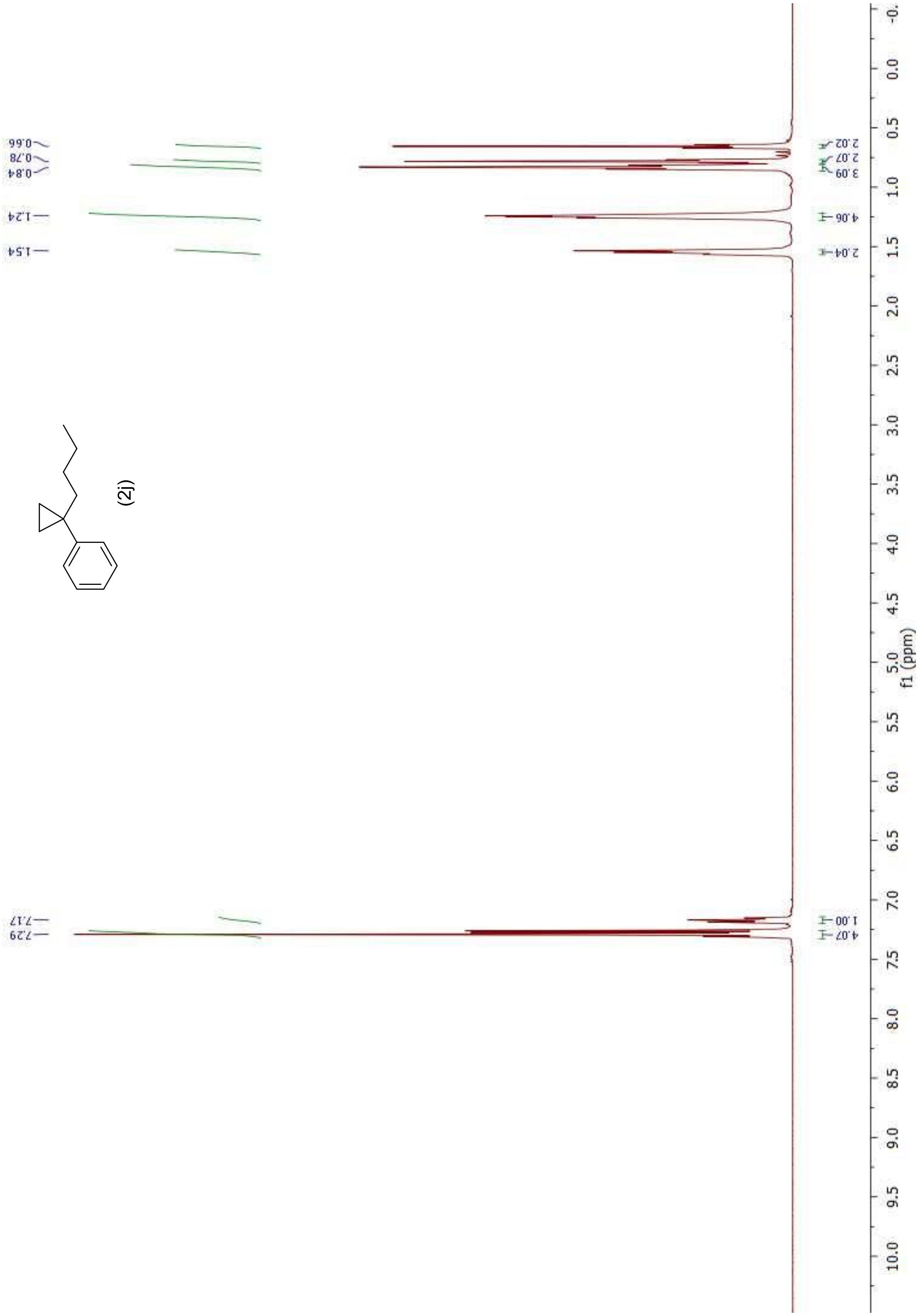

(2j)

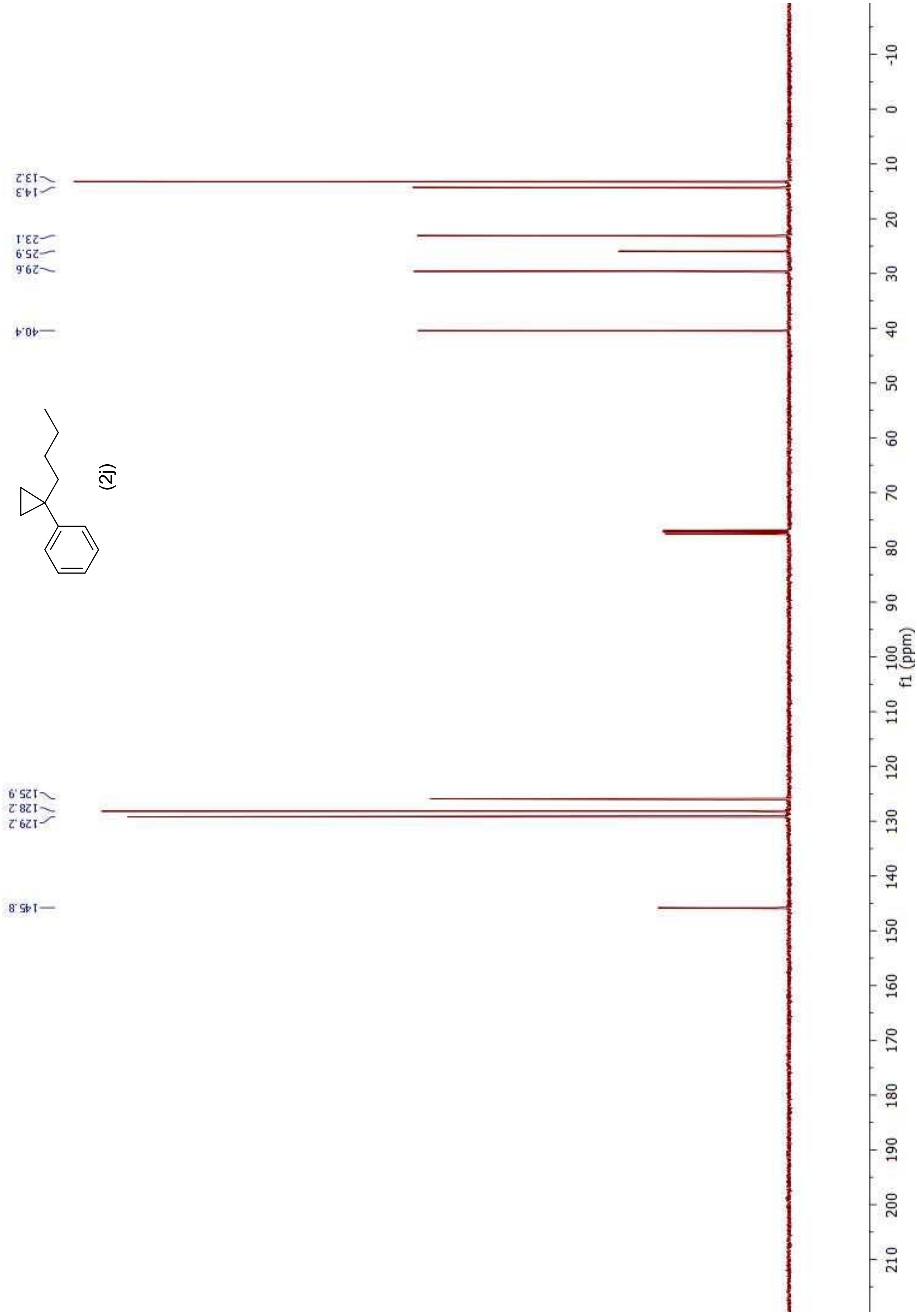
13.2
14.3
23.1
25.9
29.6
40.4
125.9
128.2
129.2
145.8
(2j)
f1 (ppm)

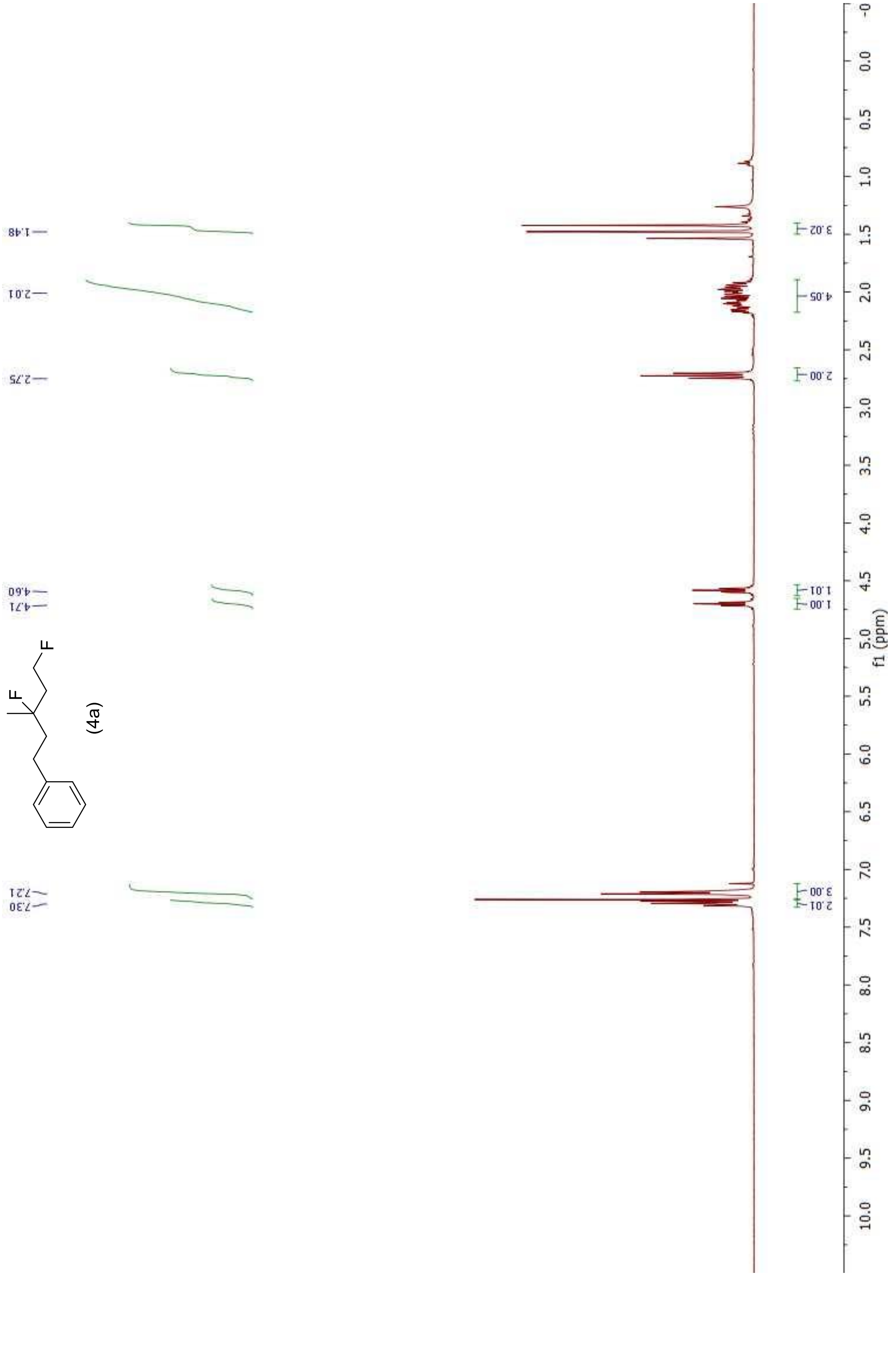
(4a)
F
F
f1 (ppm)

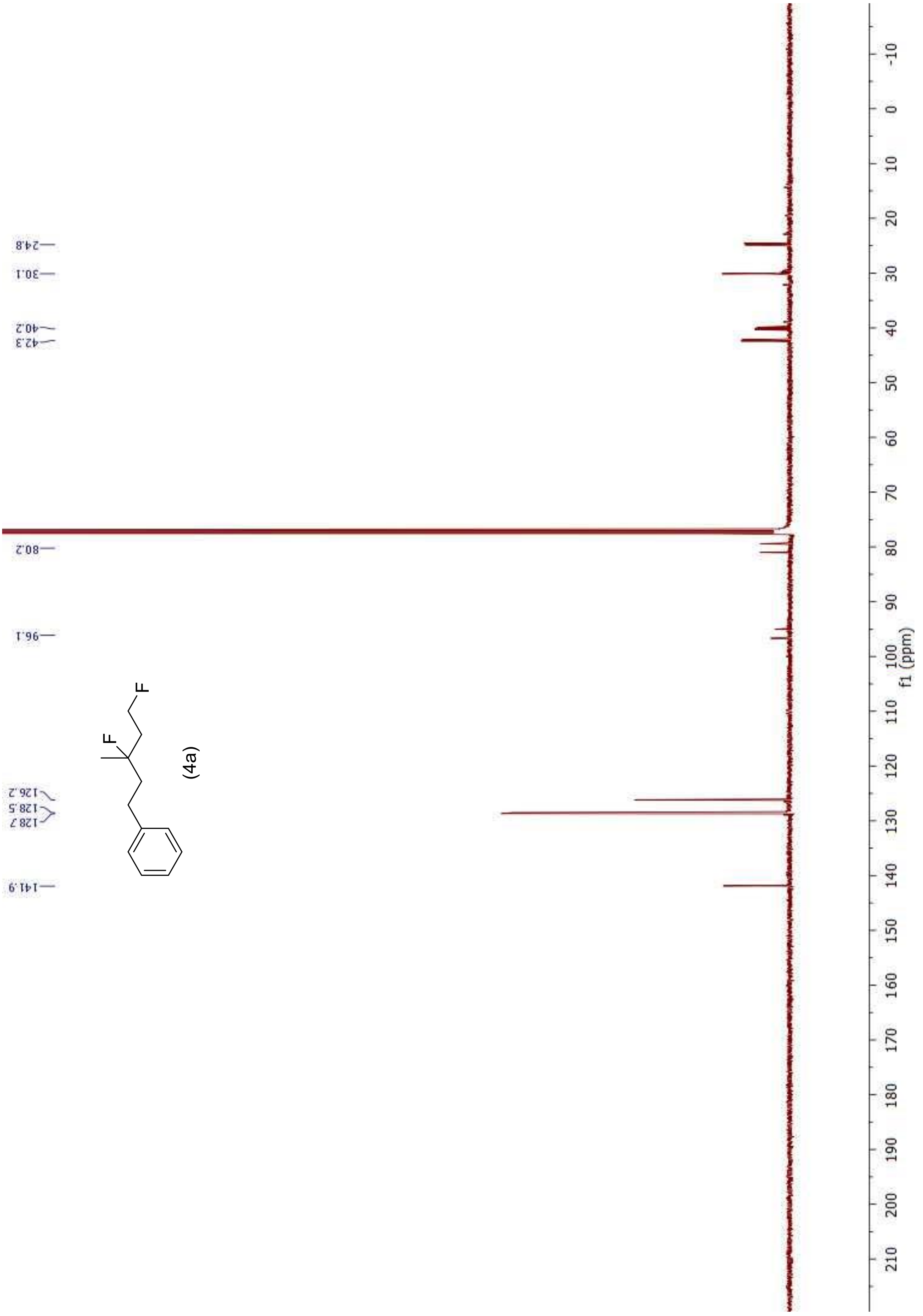
24.8
30.1
40.2
42.3
80.2
96.1
126.2
128.5
128.7
141.9
F
F
(4a)
-10
0
10
20
30
40
50
60
70
80
90
100
110
120
130
140
150
160
170
180
190
200
210
f1 (ppm)

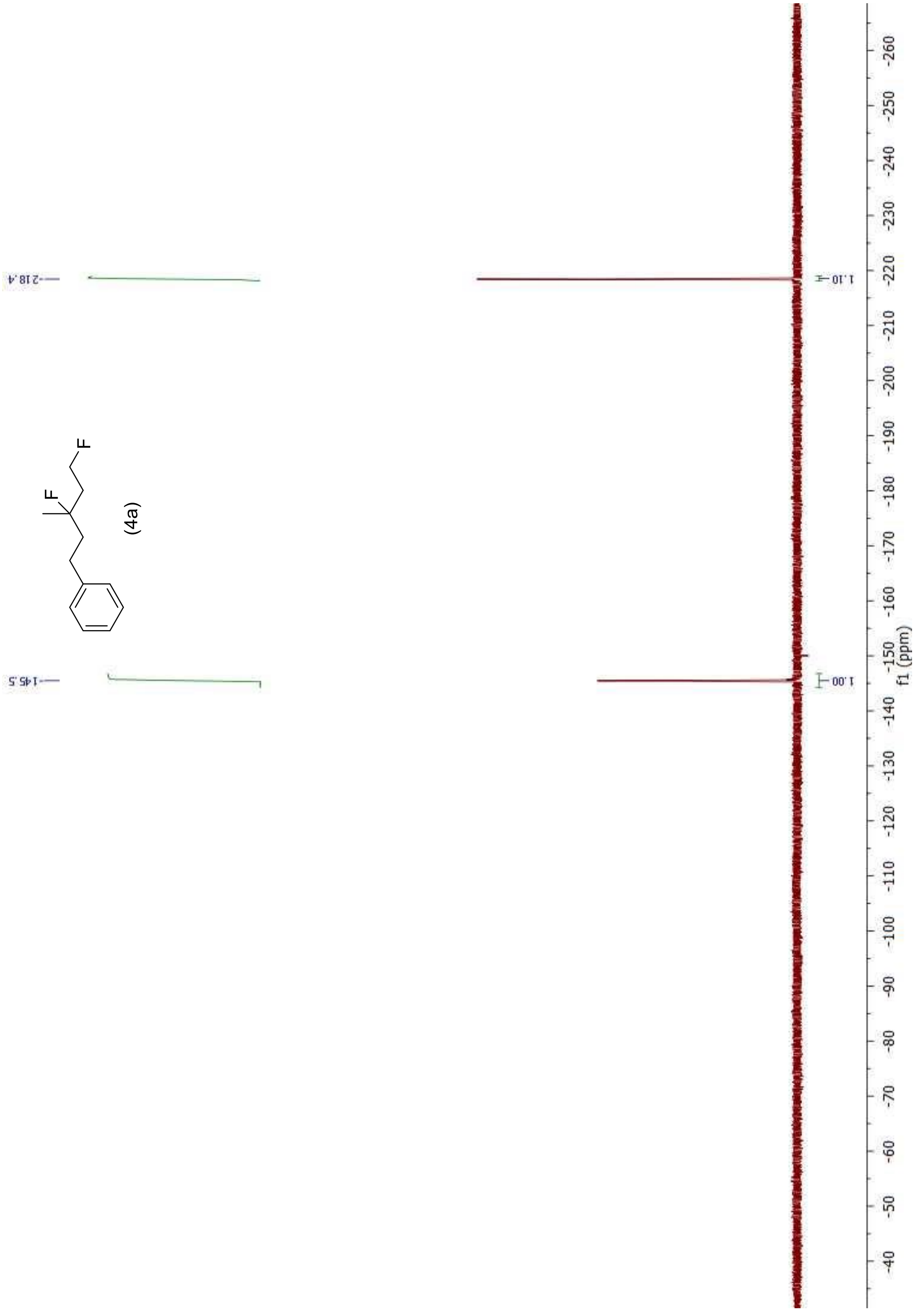

-218.4
-145.5
F
F
(4a)
1.10
1.00
-260
-250
-240
-230
-220
-210
-200
-190
-180
-170
-160
-150
-140
-130
-120
-110
-100
-90
-80
-70
-60
-50
-40
f1 (ppm)

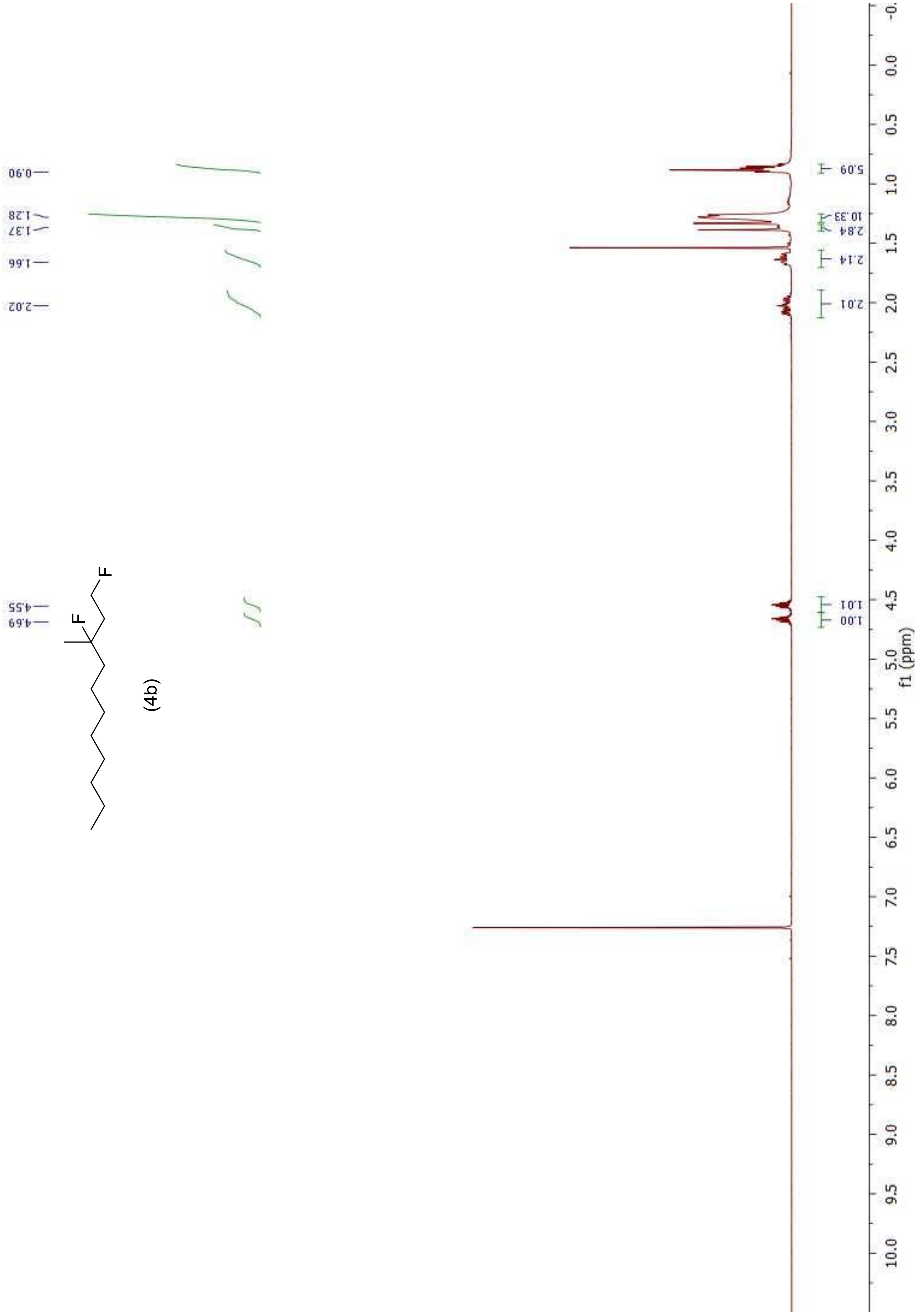
0.90
1.28
1.37
1.66
2.02
4.55
4.69
F
F
(4b)
5.09
10.33
2.84
2.14
2.01
1.01
1.00
f1 (ppm)

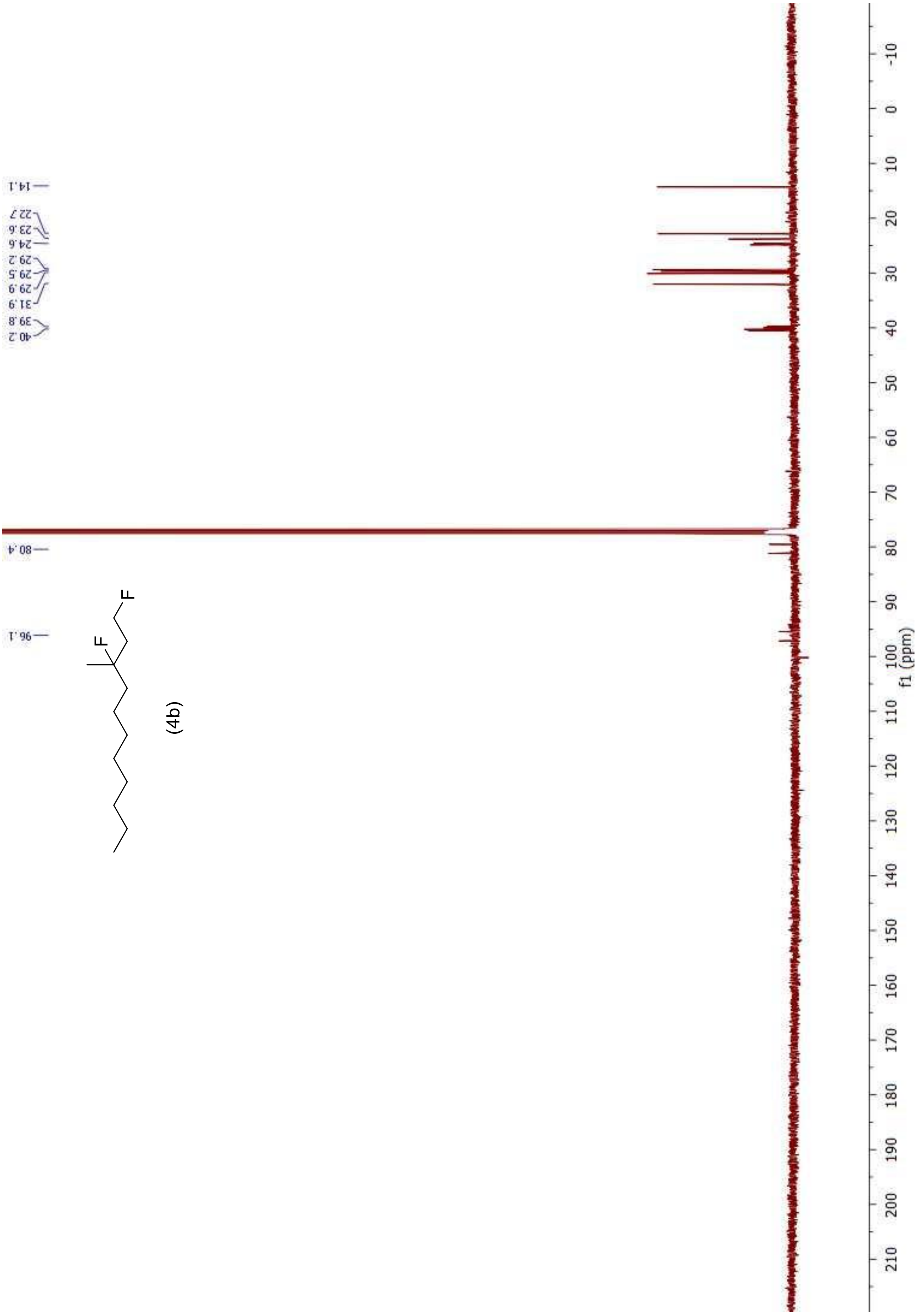
14.1
22.7
23.6
24.6
29.2
29.5
29.9
31.9
39.8
40.2
80.4
96.1
F
F
(4b)
f1 (ppm)

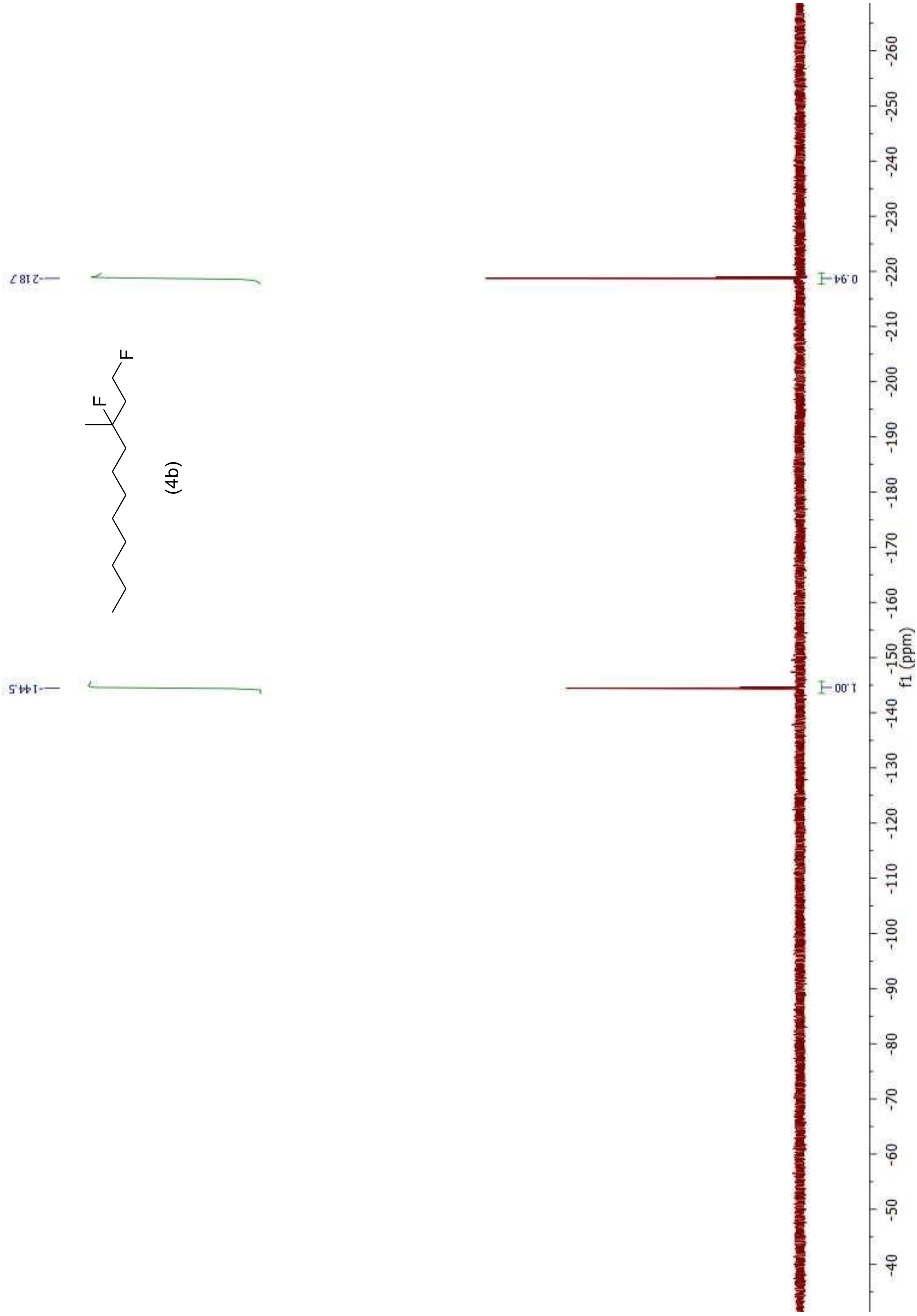
-218.7
-144.5
0.94
1.00
F
F
(4b)
f1 (ppm)
-260 -250 -240 -230 -220 -210 -200 -190 -180 -170 -160 -150 -140 -130 -120 -110 -100 -90 -80 -70 -60 -50 -40

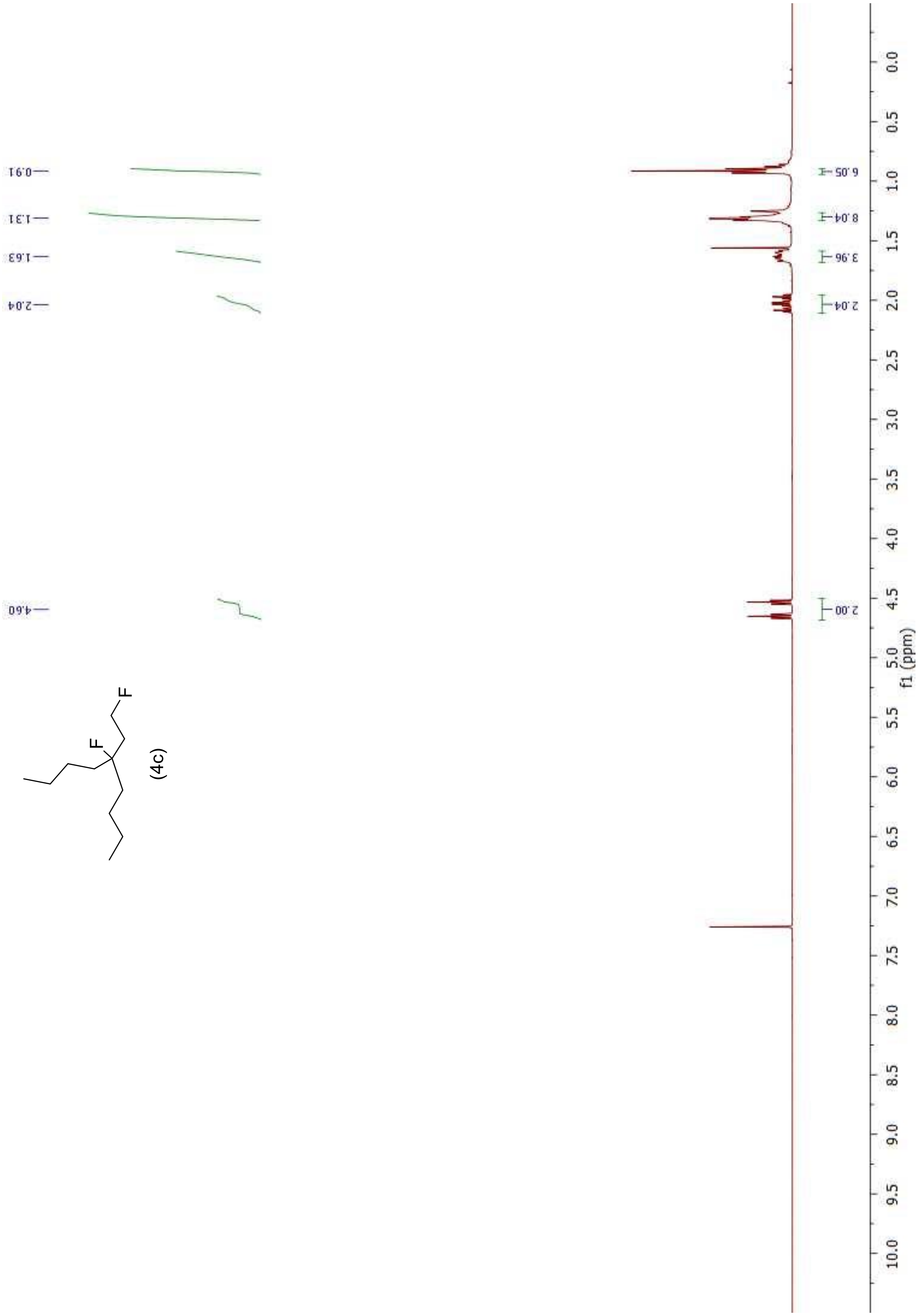

0.91
1.31
1.63
2.04
4.60
6.05
8.04
3.96
2.04
2.00
f1 (ppm)
F
F
(4c)

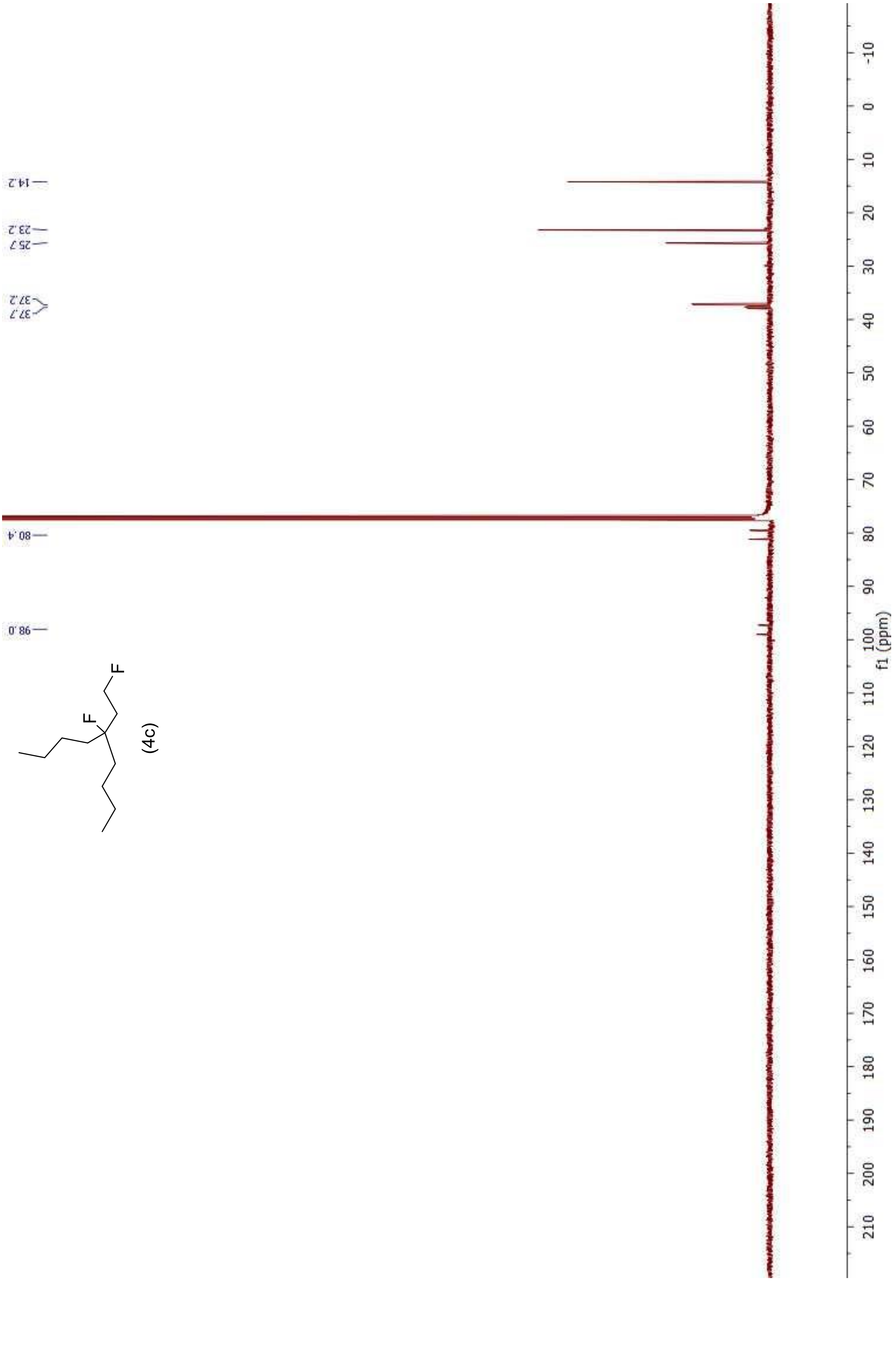
14.2
23.2
25.7
37.2
37.7
80.4
98.0
F
F
(4c)
f1 (ppm)

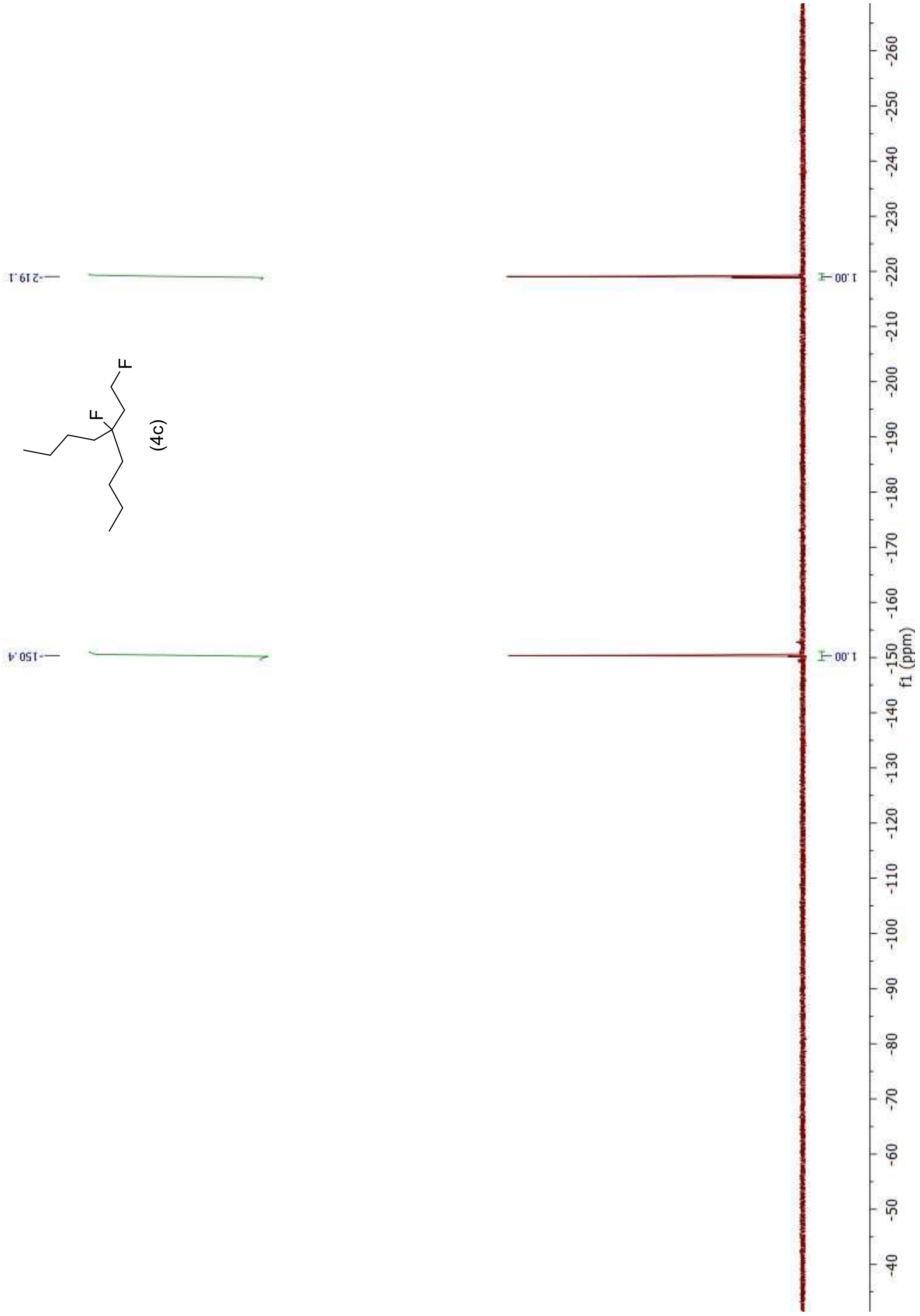

-219.1
-150.4
F
F
(4c)
1.00
1.00
-260 -250 -240 -230 -220 -210 -200 -190 -180 -170 -160 -150 -140 -130 -120 -110 -100 -90 -80 -70 -60 -50 -40
f1 (ppm)

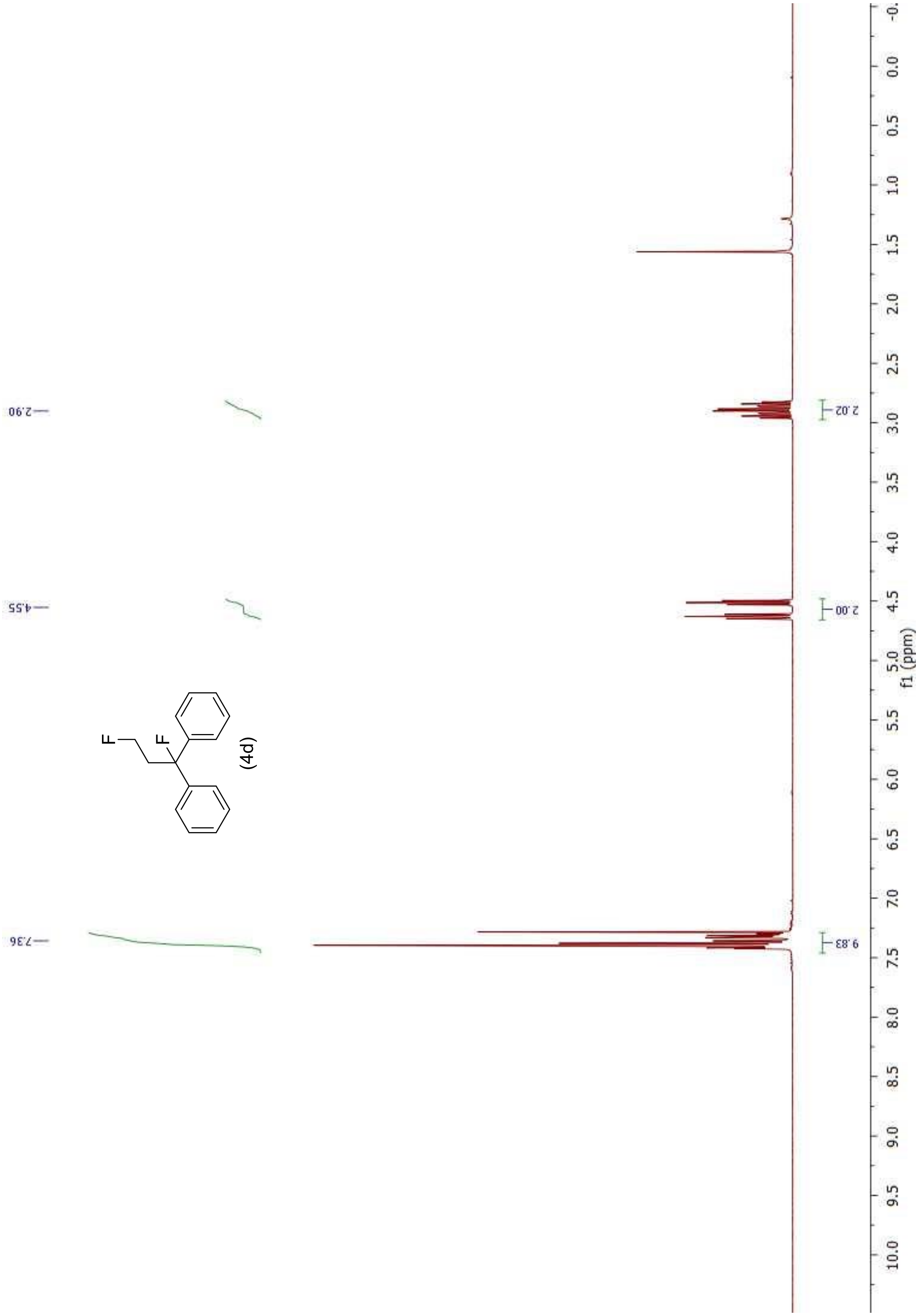
2.90
4.55
7.36
(4d)
2.02
2.00
9.83
f1 (ppm)

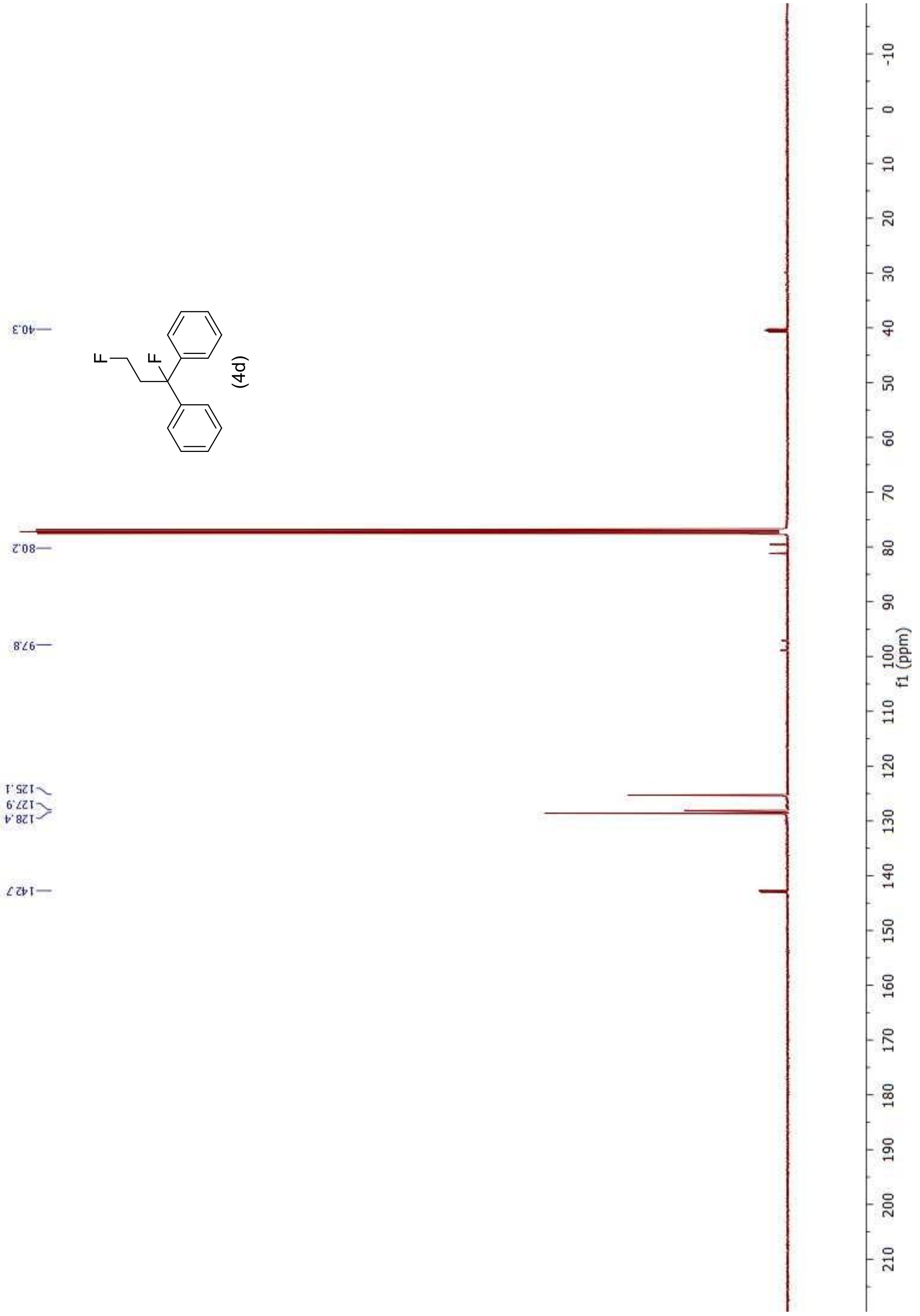
40.3
80.2
97.8
125.1
127.9
128.4
142.7
F
F
(4d)
f1 (ppm)

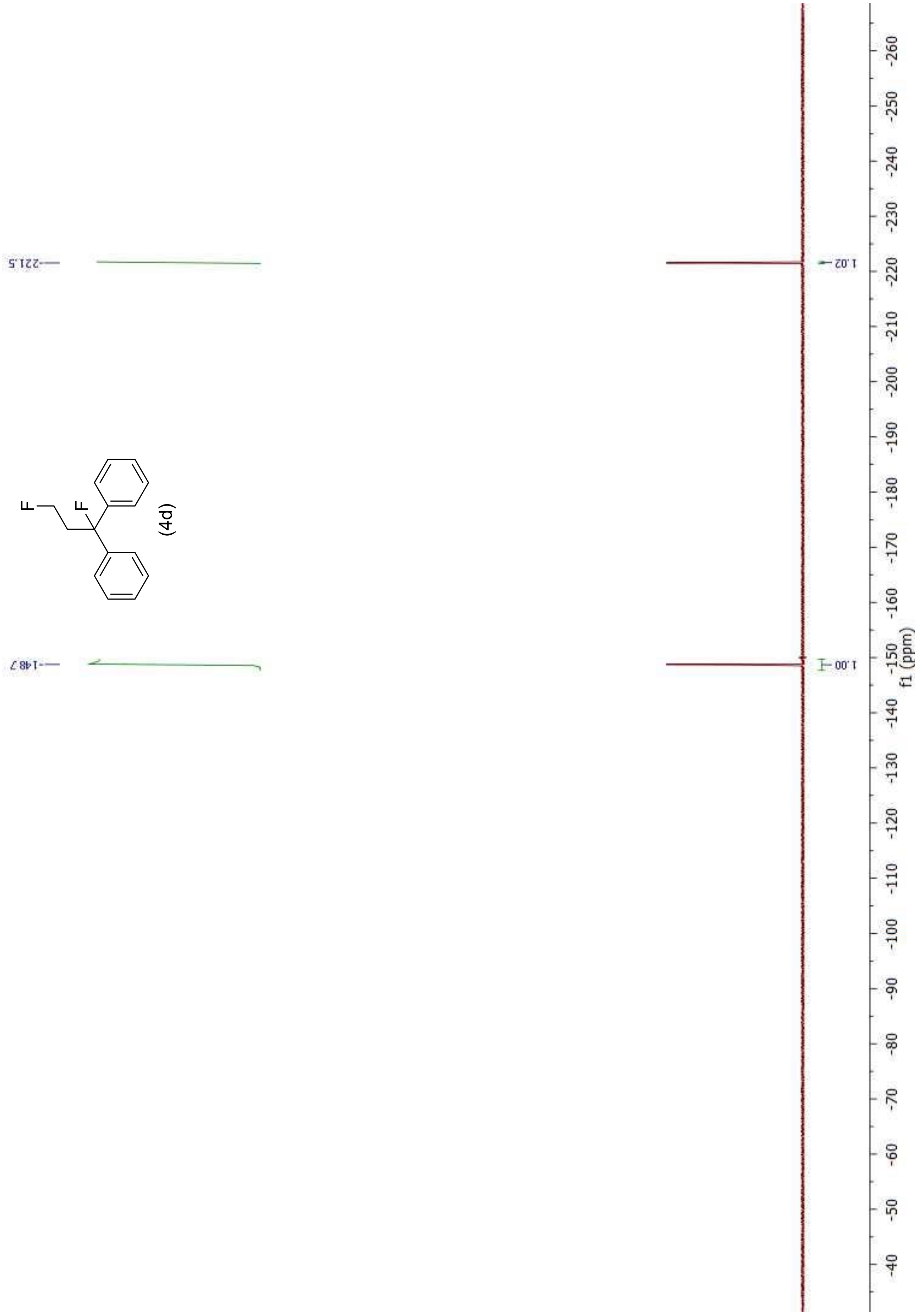

-221.5
-148.7
F
F
(4d)
1.02
1.00
-260 -250 -240 -230 -220 -210 -200 -190 -180 -170 -160 -150 -140 -130 -120 -110 -100 -90 -80 -70 -60 -50 -40
f1 (ppm)

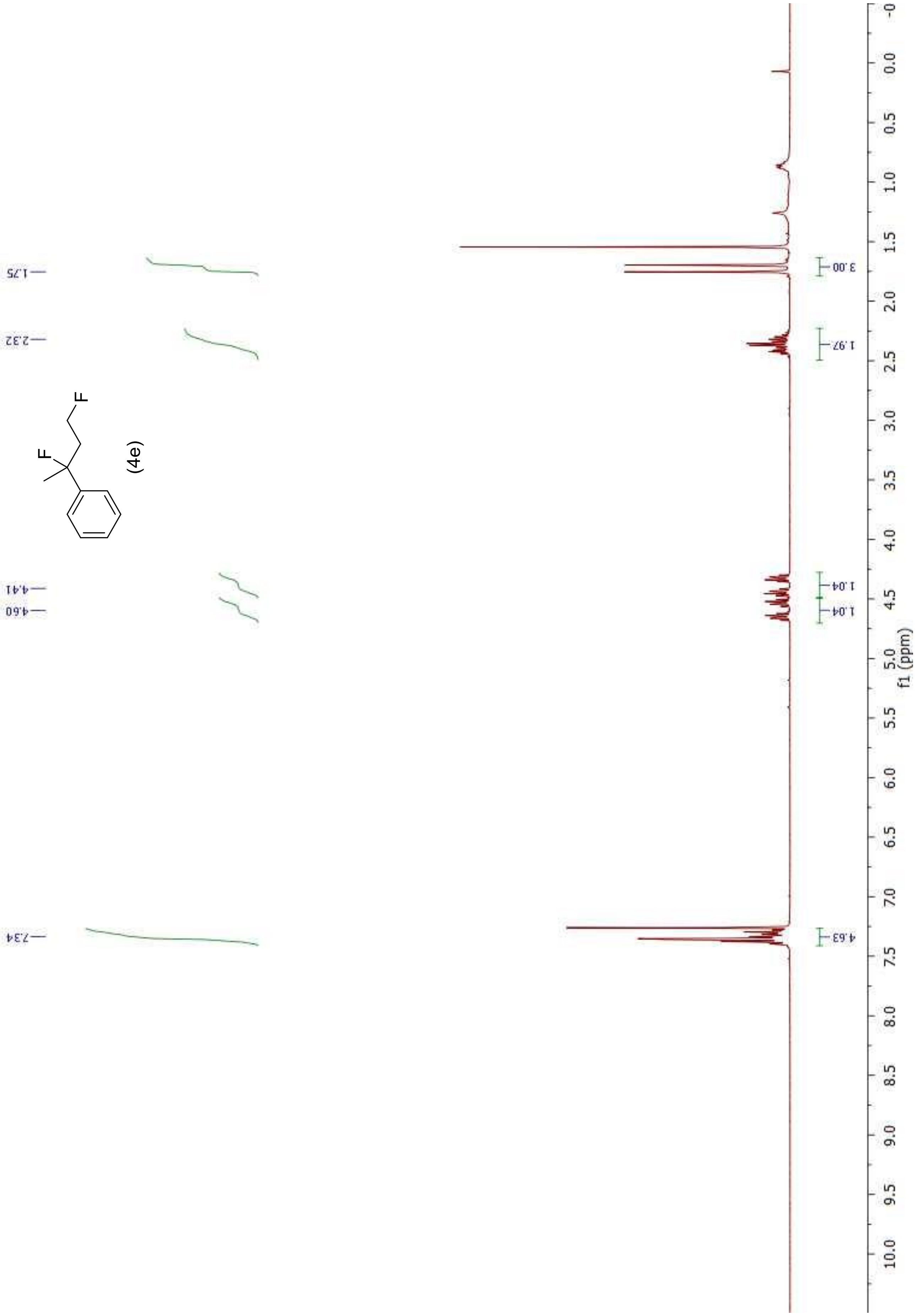

1.75
2.32
4.41
4.60
7.34
F
F
(4e)
3.00
1.97
1.04
1.04
4.63
f1 (ppm)

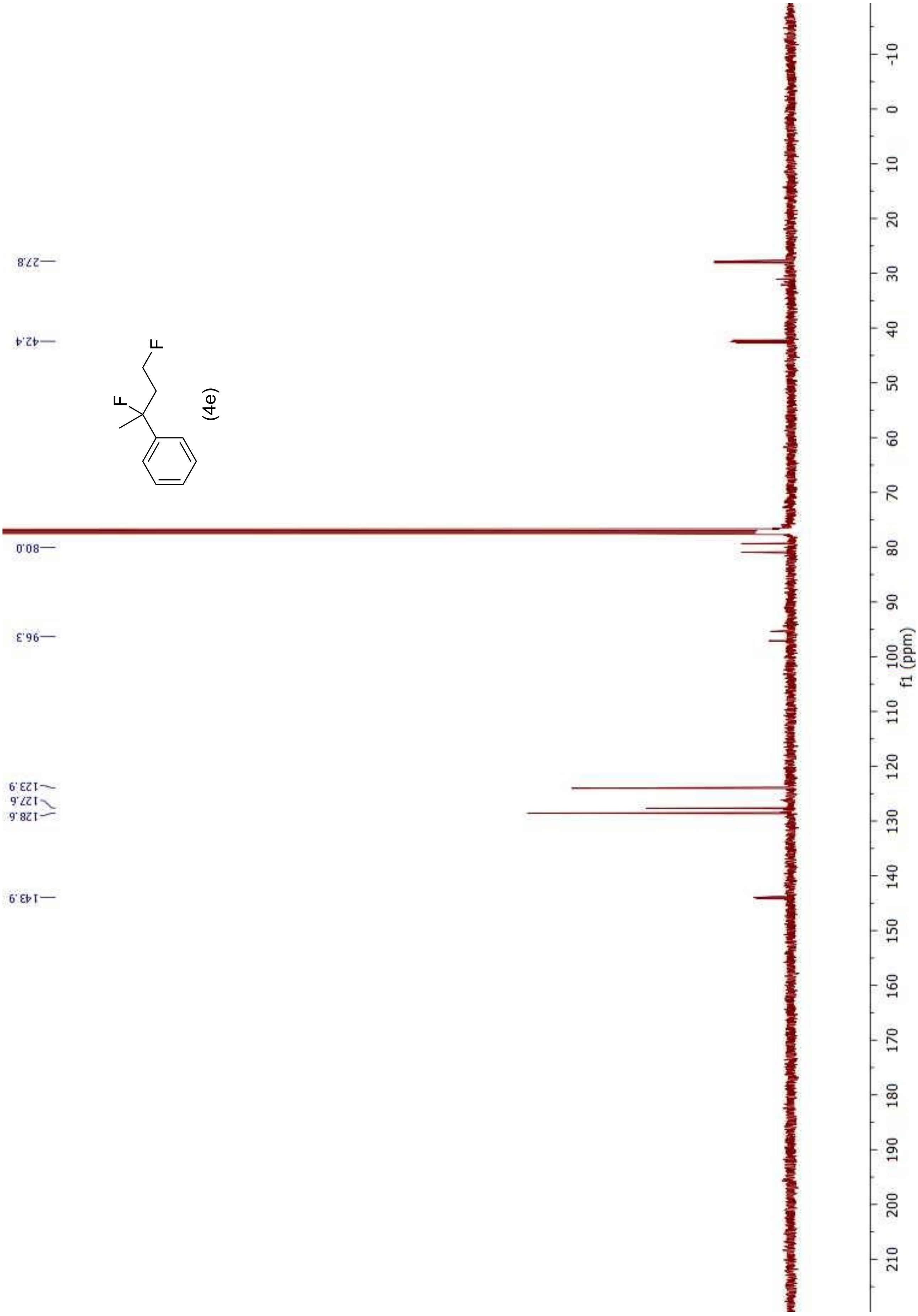
27.8
42.4
80.0
96.3
123.9
127.6
128.6
143.9
F
F
(4e)
f1 (ppm)

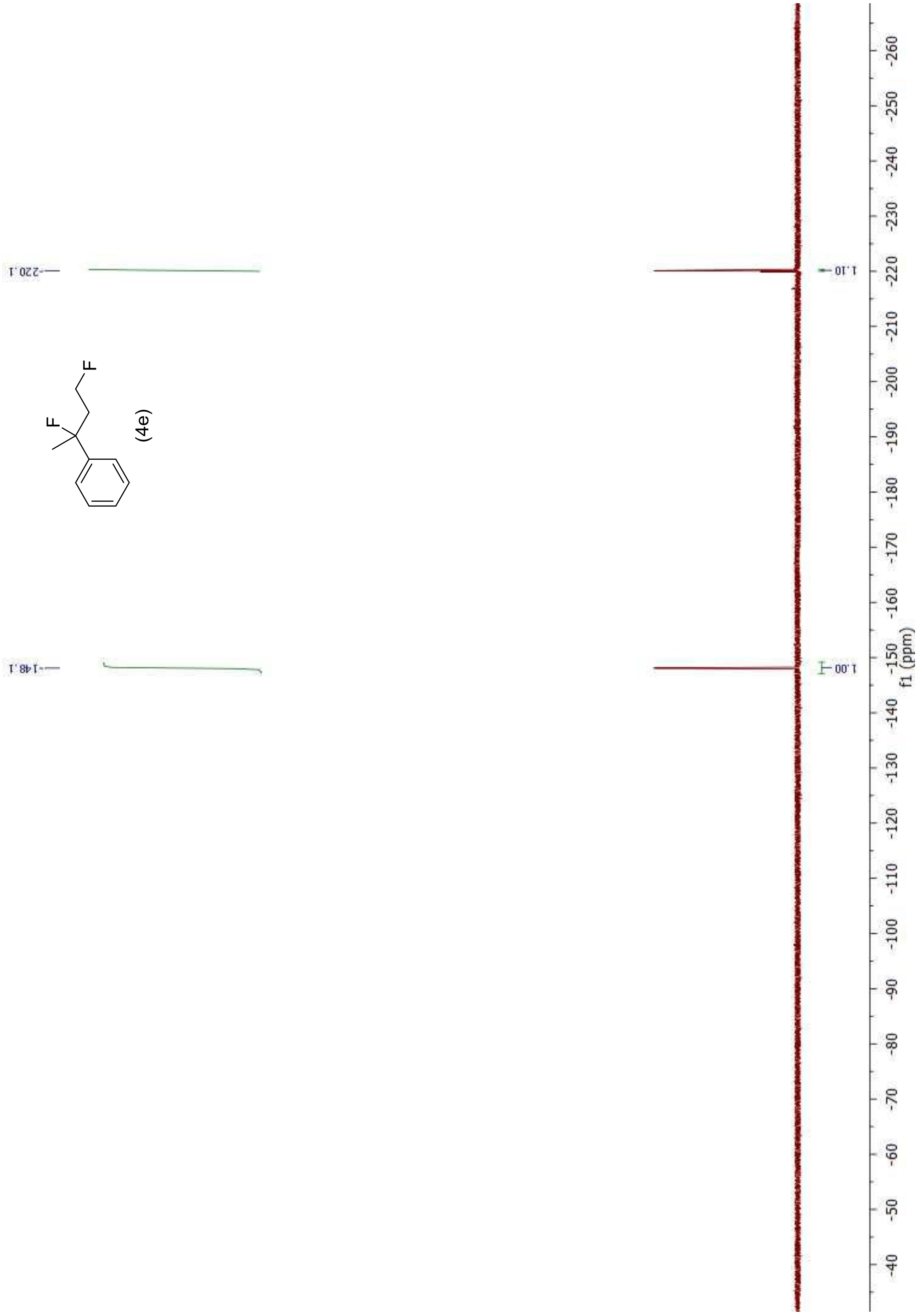
-220.1
-148.1
(4e)
1.10
1.00
-260
-250
-240
-230
-220
-210
-200
-190
-180
-170
-160
-150
-140
-130
-120
-110
-100
-90
-80
-70
-60
-50
-40
f1 (ppm)

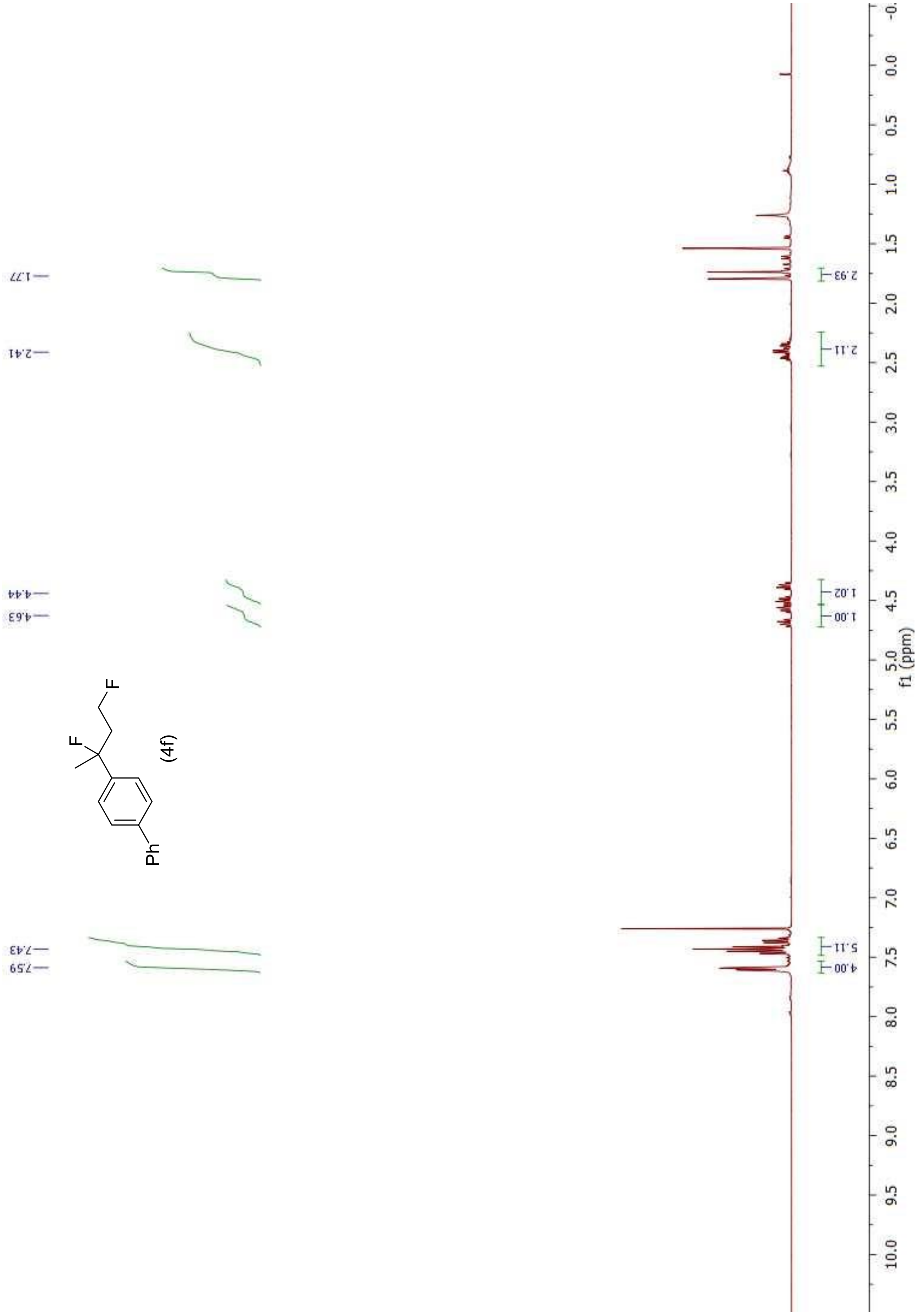
F
F
Ph
(4f)
1.77
2.41
4.44
4.63
7.43
7.59
2.93
2.11
1.02
1.00
5.11
4.00
f1 (ppm)

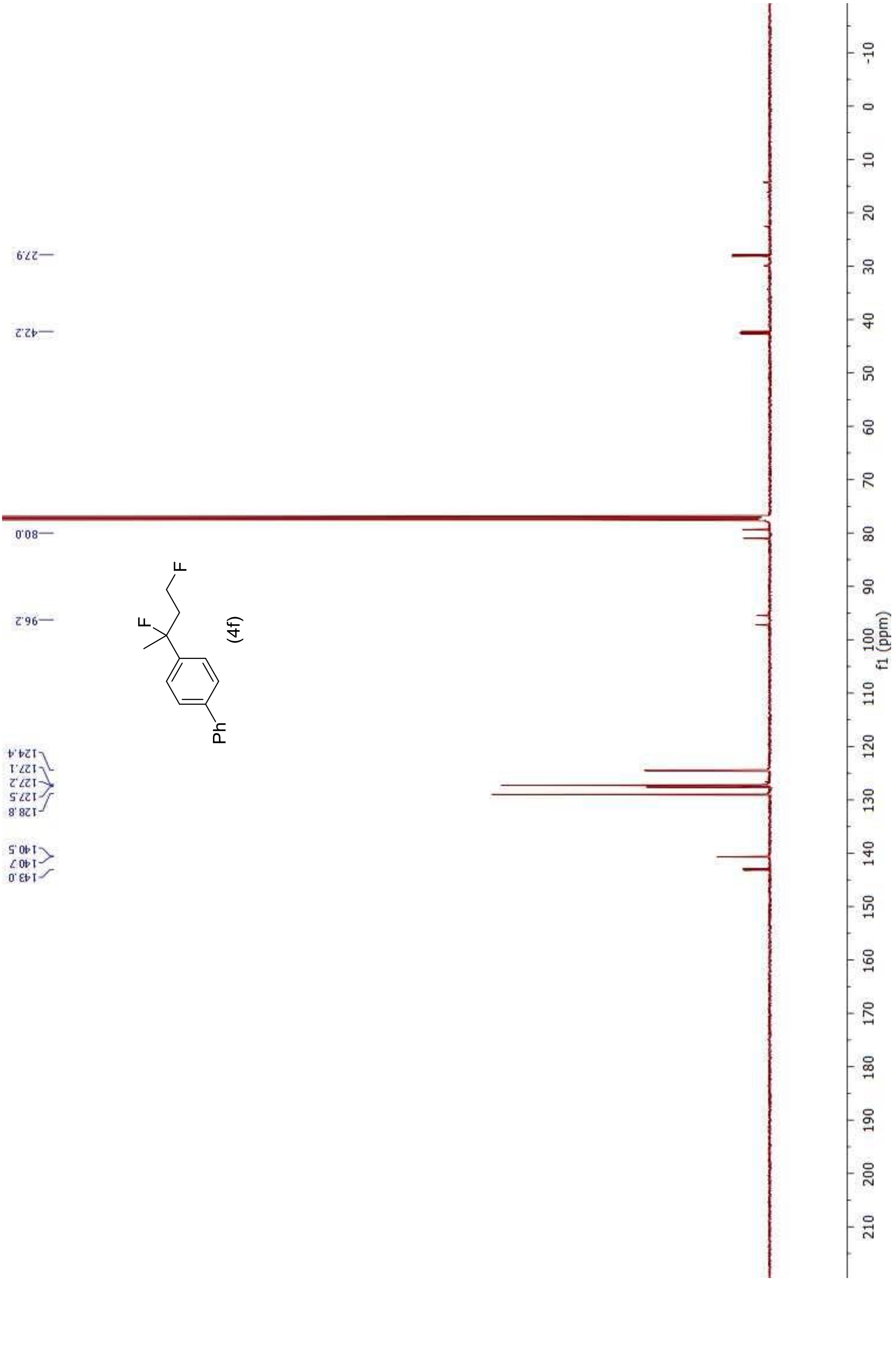
27.9
42.2
80.0
96.2
124.4
127.1
127.2
127.5
128.8
140.5
140.7
143.0
F
F
Ph
(4f)
f1 (ppm)

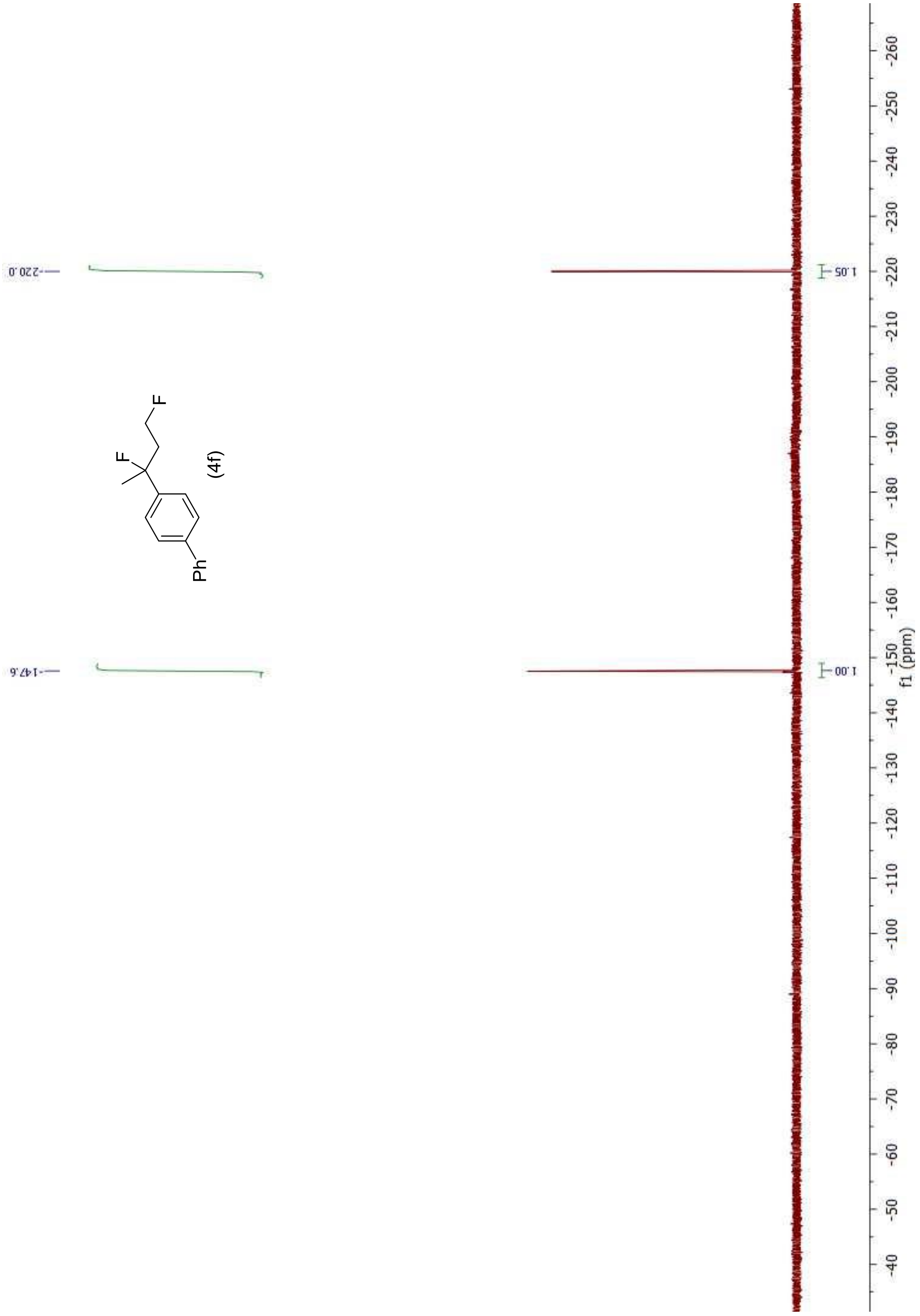
Ph
F
F
(4f)
-220.0
-147.6
1.05
1.00
f1 (ppm)

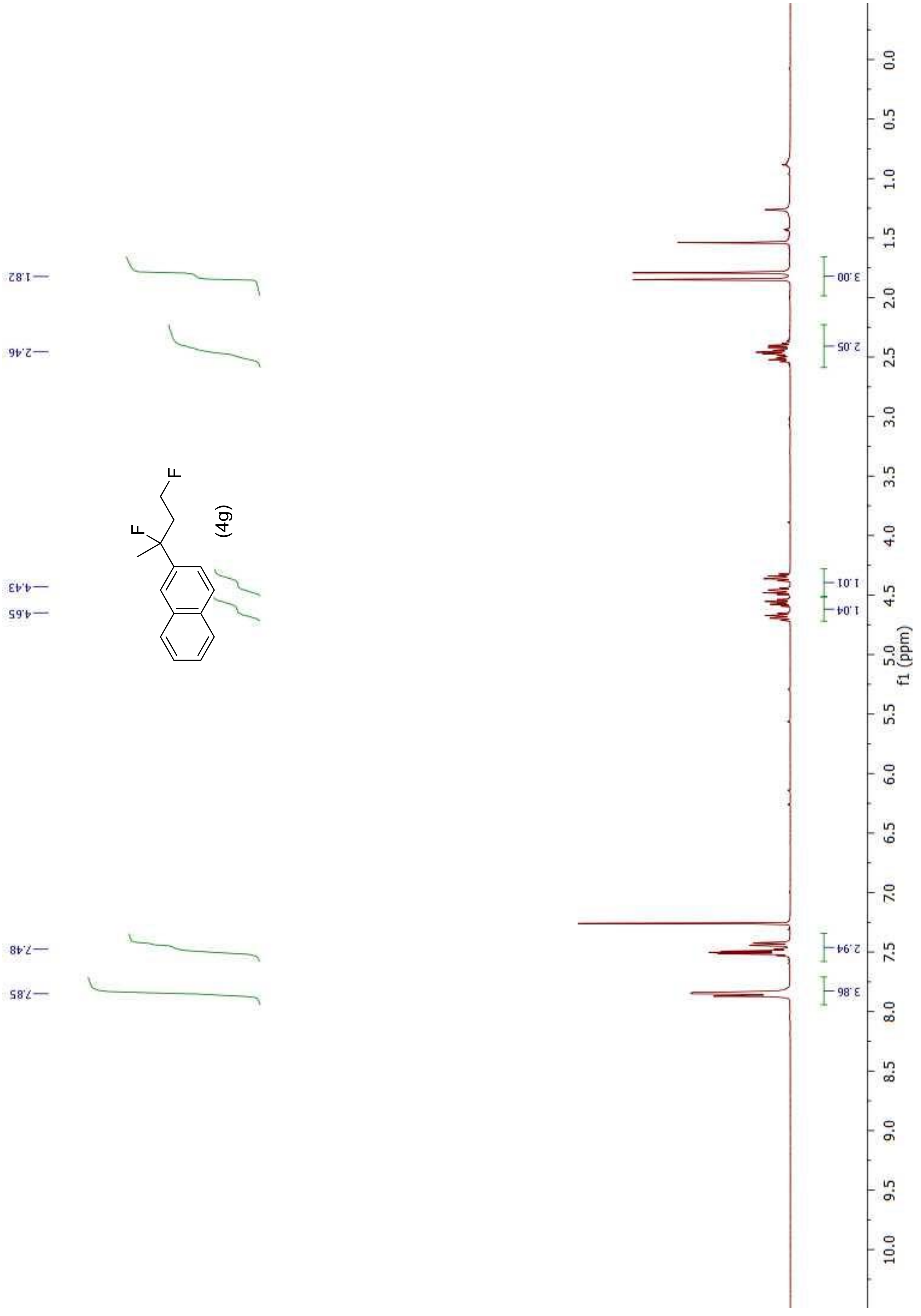
1.82
2.46
4.43
4.65
7.48
7.85
(4g)
3.00
2.05
1.01
1.04
2.94
3.86
f1 (ppm)

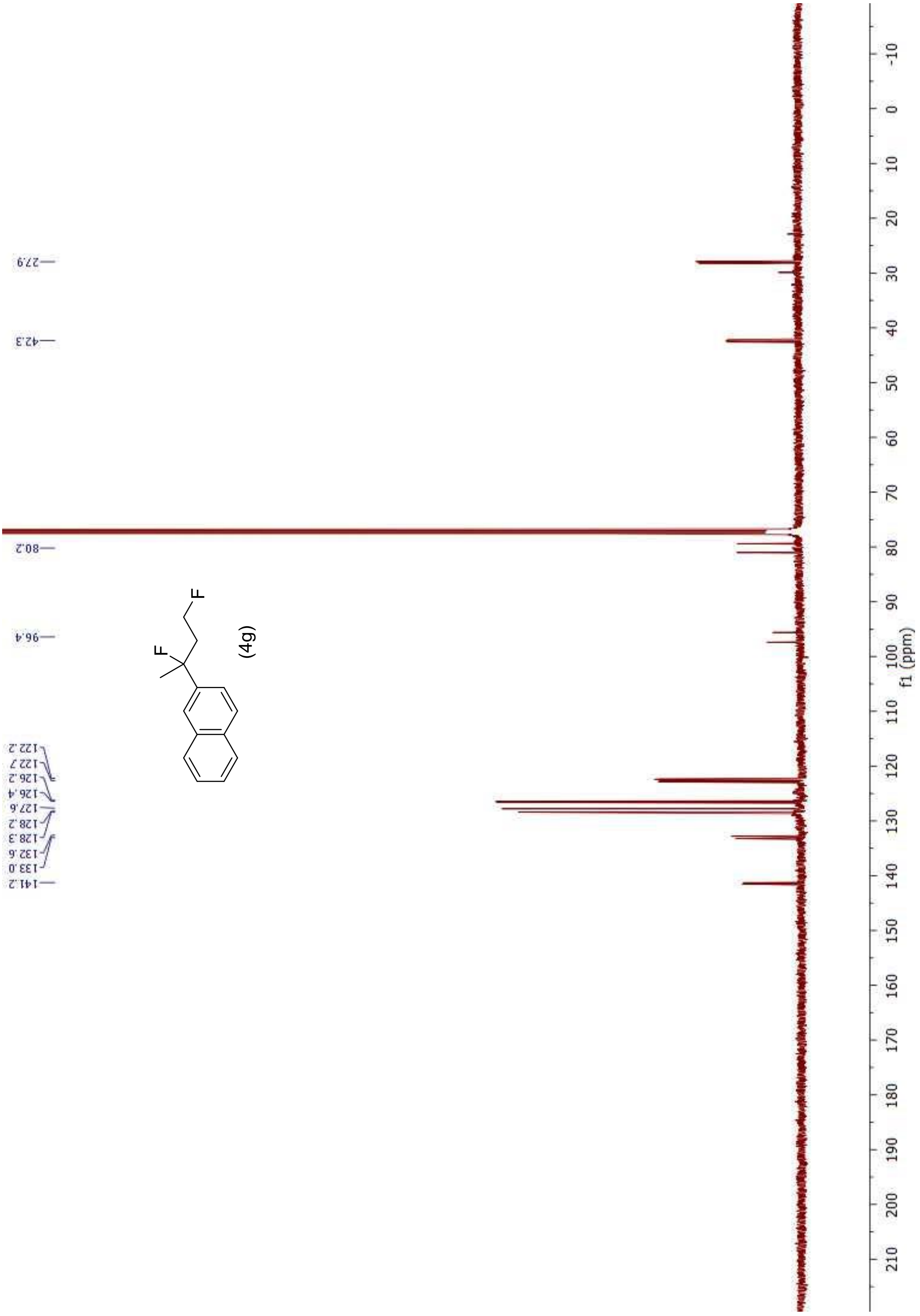
27.9
42.3
80.2
96.4
122.2
122.7
126.2
126.4
127.6
128.2
128.3
132.6
133.0
141.2
F
F
(4g)
f1 (ppm)

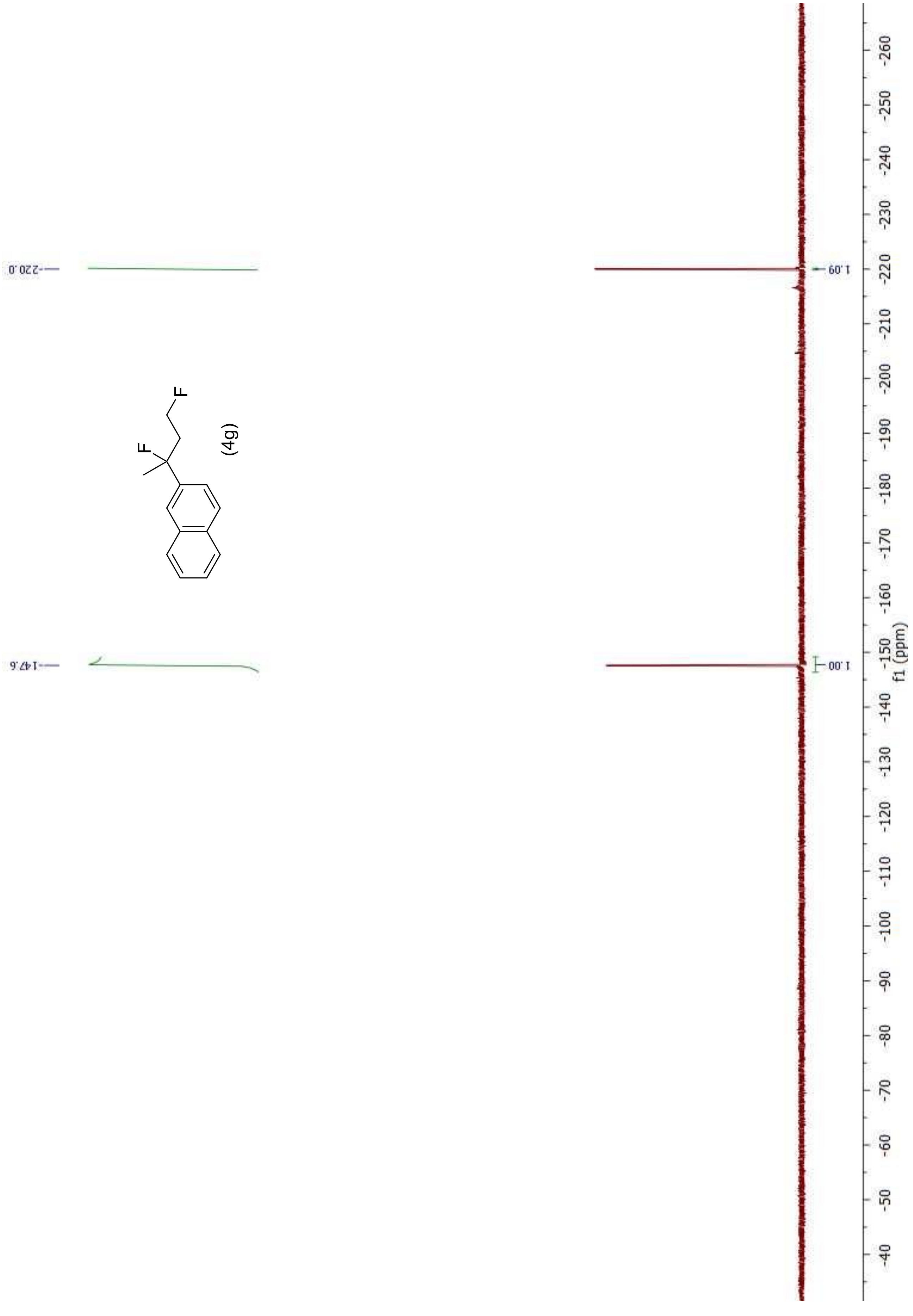
-220.0
-147.6
1.09
1.00
(4g)
F
F
f1 (ppm)
-260 -250 -240 -230 -220 -210 -200 -190 -180 -170 -160 -150 -140 -130 -120 -110 -100 -90 -80 -70 -60 -50 -40

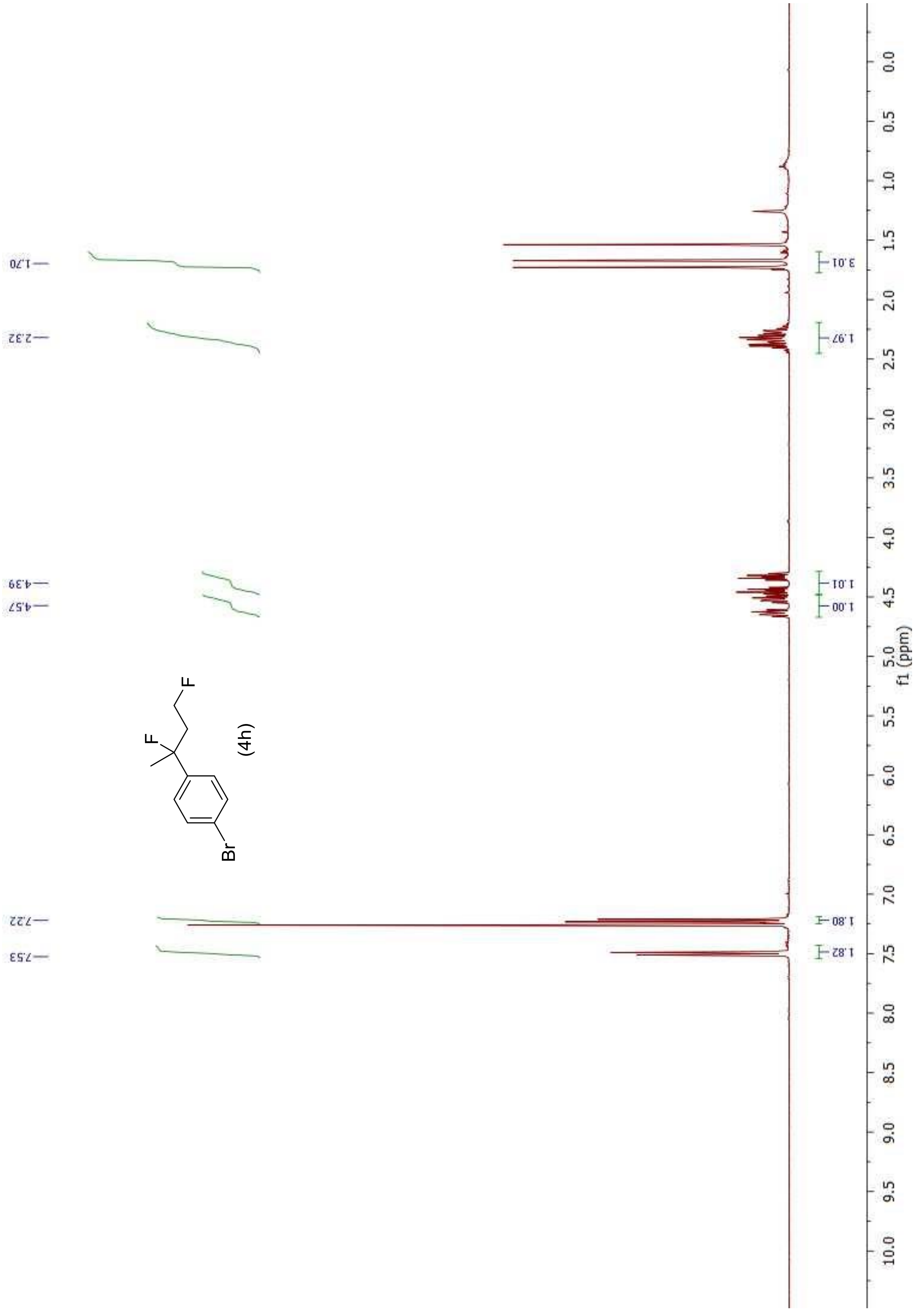
1.70
2.32
4.39
4.57
7.22
7.53
3.01
1.97
1.01
1.00
1.80
1.82
f1 (ppm)
F
F
Br
(4h)

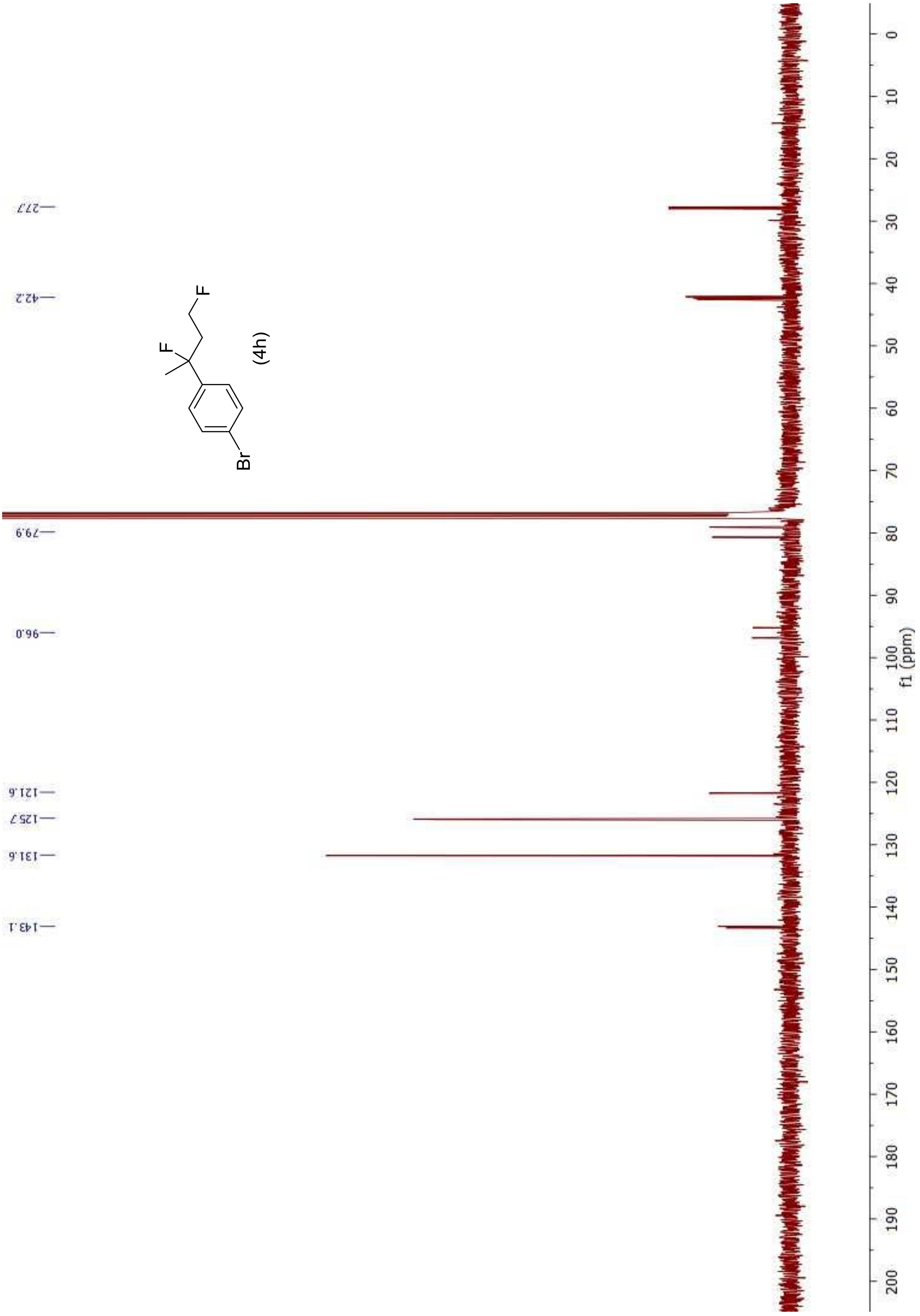

27.7
42.2
79.9
96.0
121.6
125.7
131.6
143.1
F
F
Br
(4h)
f1 (ppm)

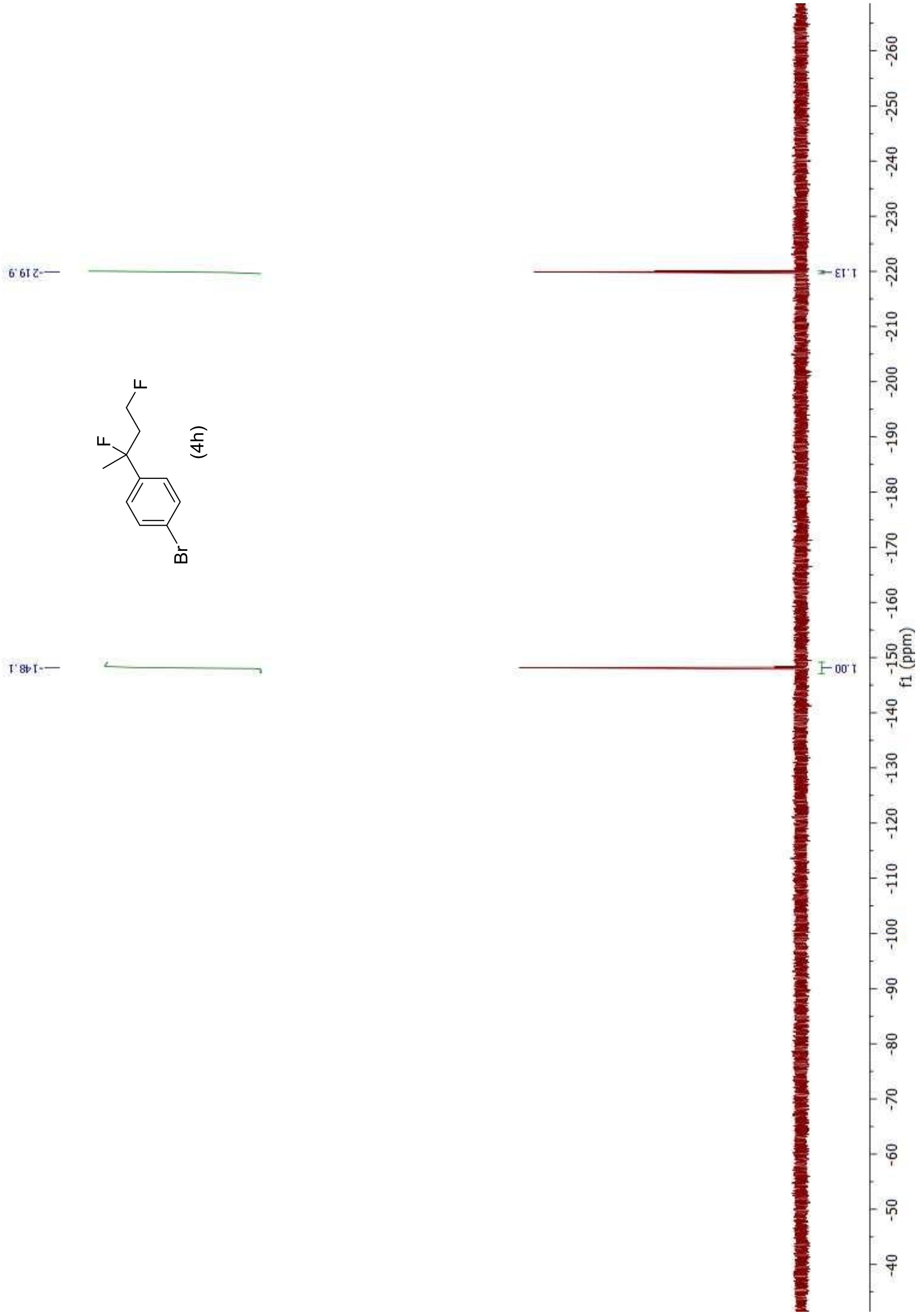

-219.9
-148.1
F
F
Br
(4h)
1.13
1.00
f1 (ppm)

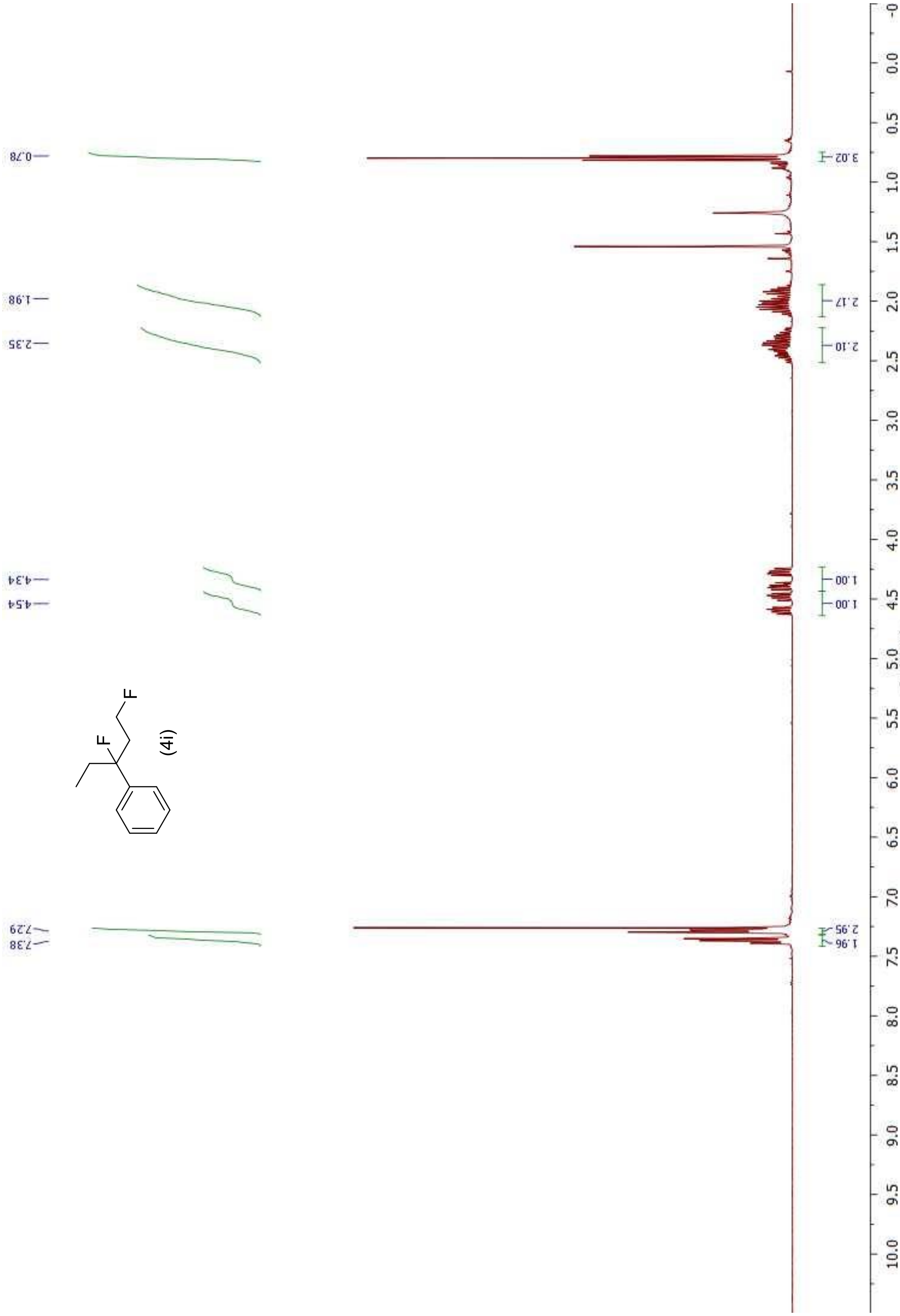

0.78
1.98
2.35
4.34
4.54
7.29
7.38
F
F
(4i)
3.02
2.17
2.10
1.00
1.00
2.95
1.96
f1 (ppm)

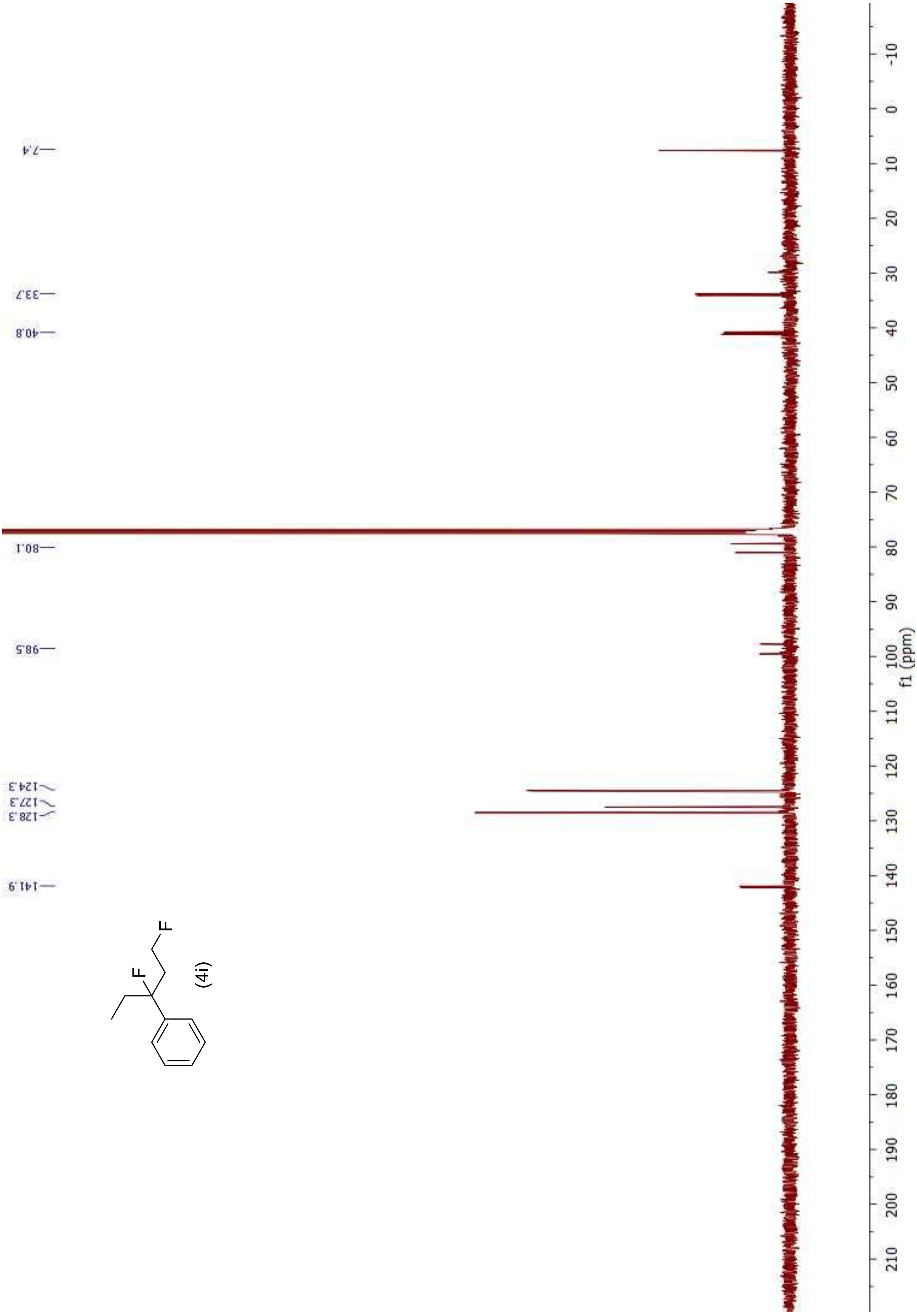
7.4
33.7
40.8
80.1
98.5
124.3
127.3
128.3
141.9
f1 (ppm)
F
F
(4i)

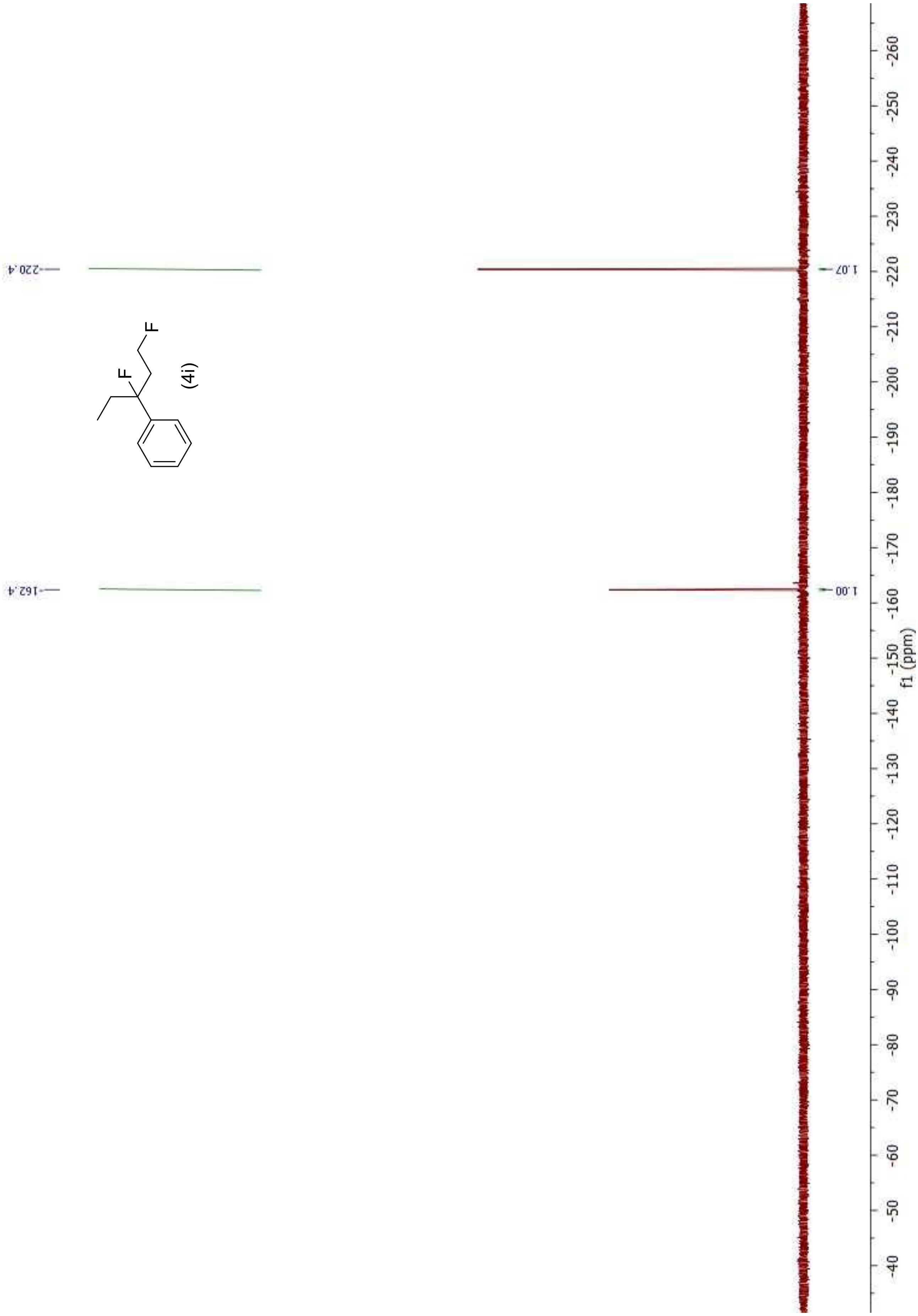
-220.4
-162.4
F
F
(4i)
1.07
1.00
-260 -250 -240 -230 -220 -210 -200 -190 -180 -170 -160 -150 -140 -130 -120 -110 -100 -90 -80 -70 -60 -50 -40
f1 (ppm)

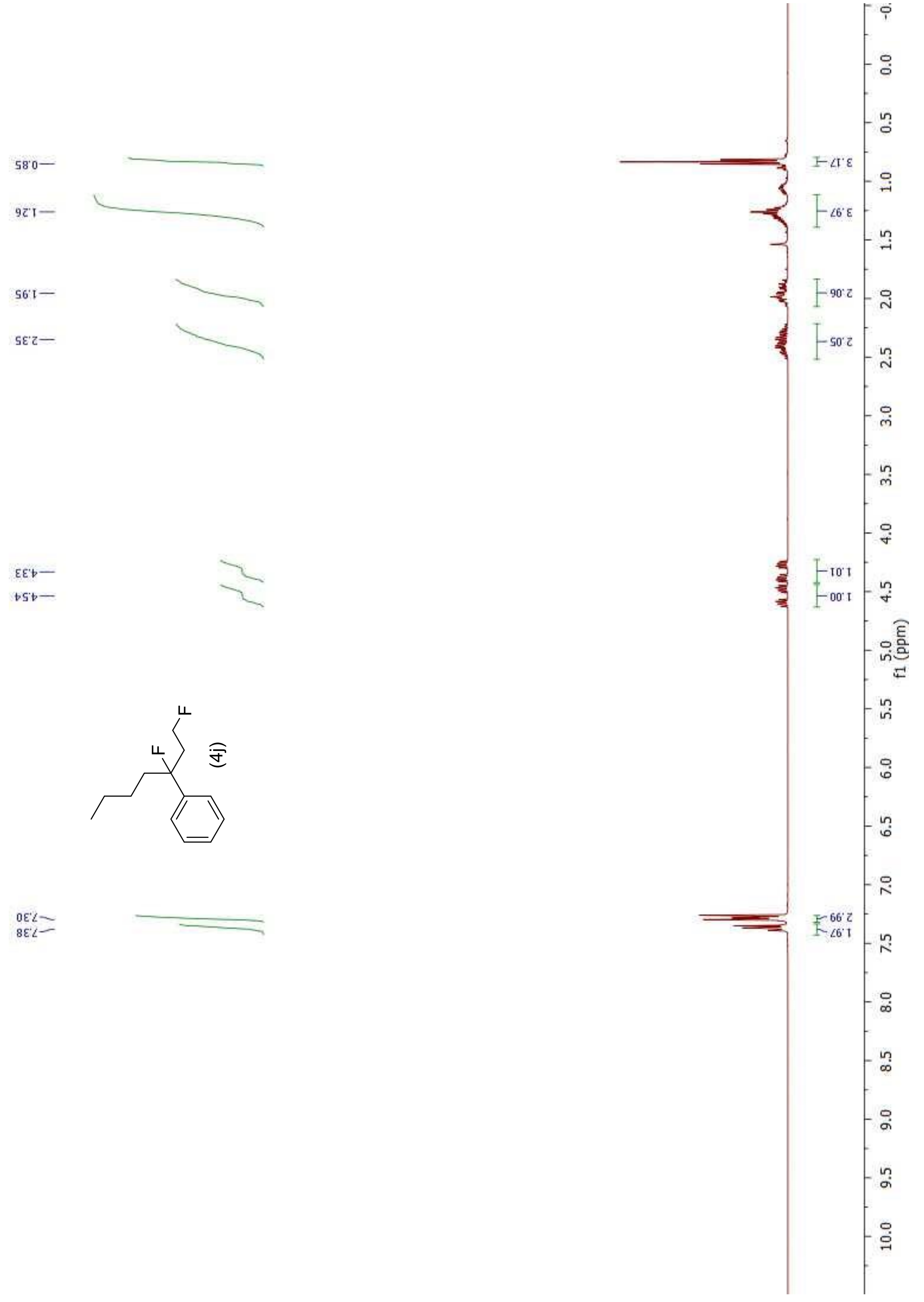
0.85
1.26
1.95
2.35
4.33
4.54
7.30
7.38
3.17
3.97
2.06
2.05
1.01
1.00
2.99
1.97
F
F
(4j)
-0.
0.0
0.5
1.0
1.5
2.0
2.5
3.0
3.5
4.0
4.5
5.0
5.5
6.0
6.5
7.0
7.5
8.0
8.5
9.0
9.5
10.0
f1 (ppm)

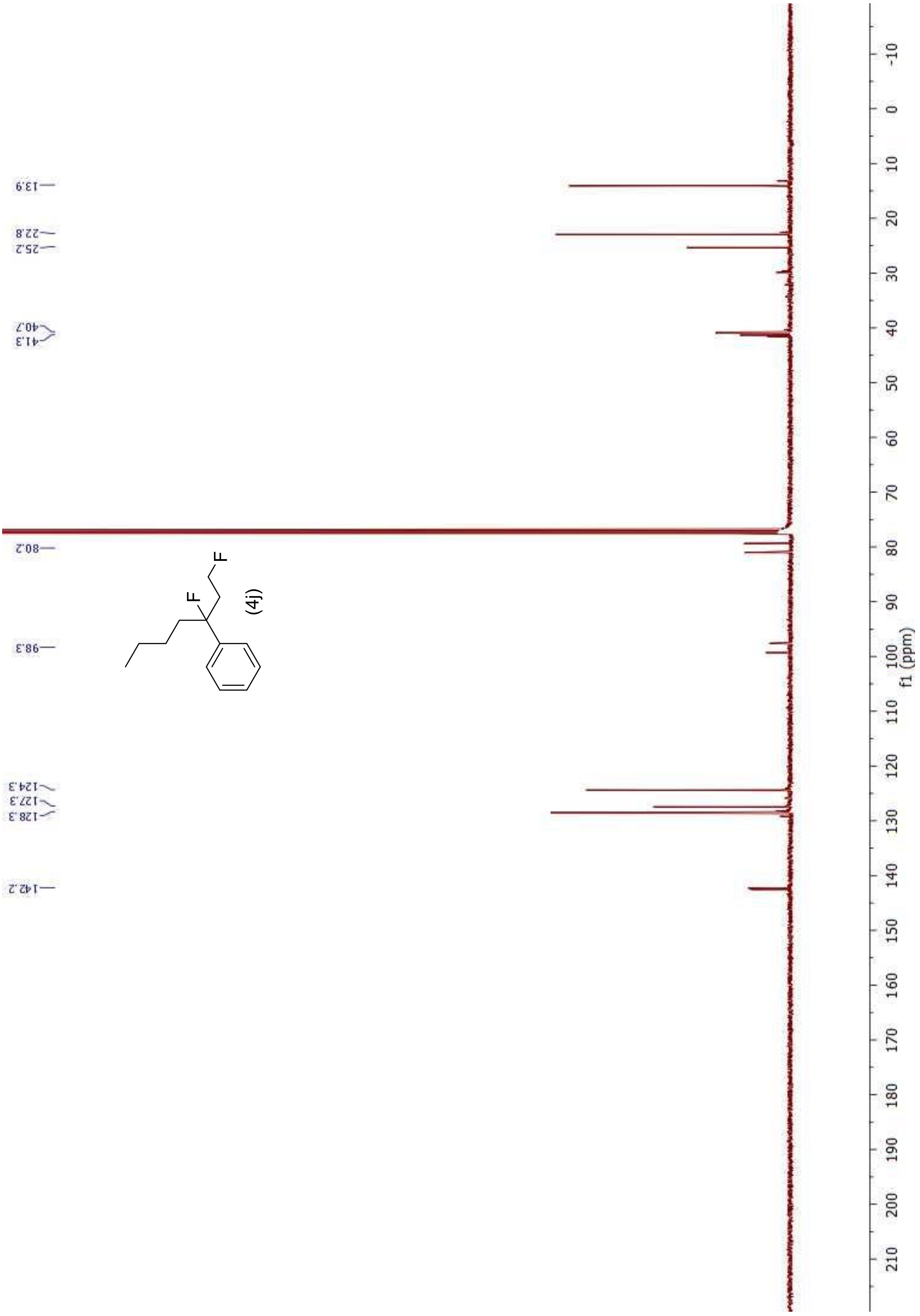
13.9
22.8
25.2
40.7
41.3
80.2
98.3
124.3
127.3
128.3
142.2
F
F
(4j)
f1 (ppm)

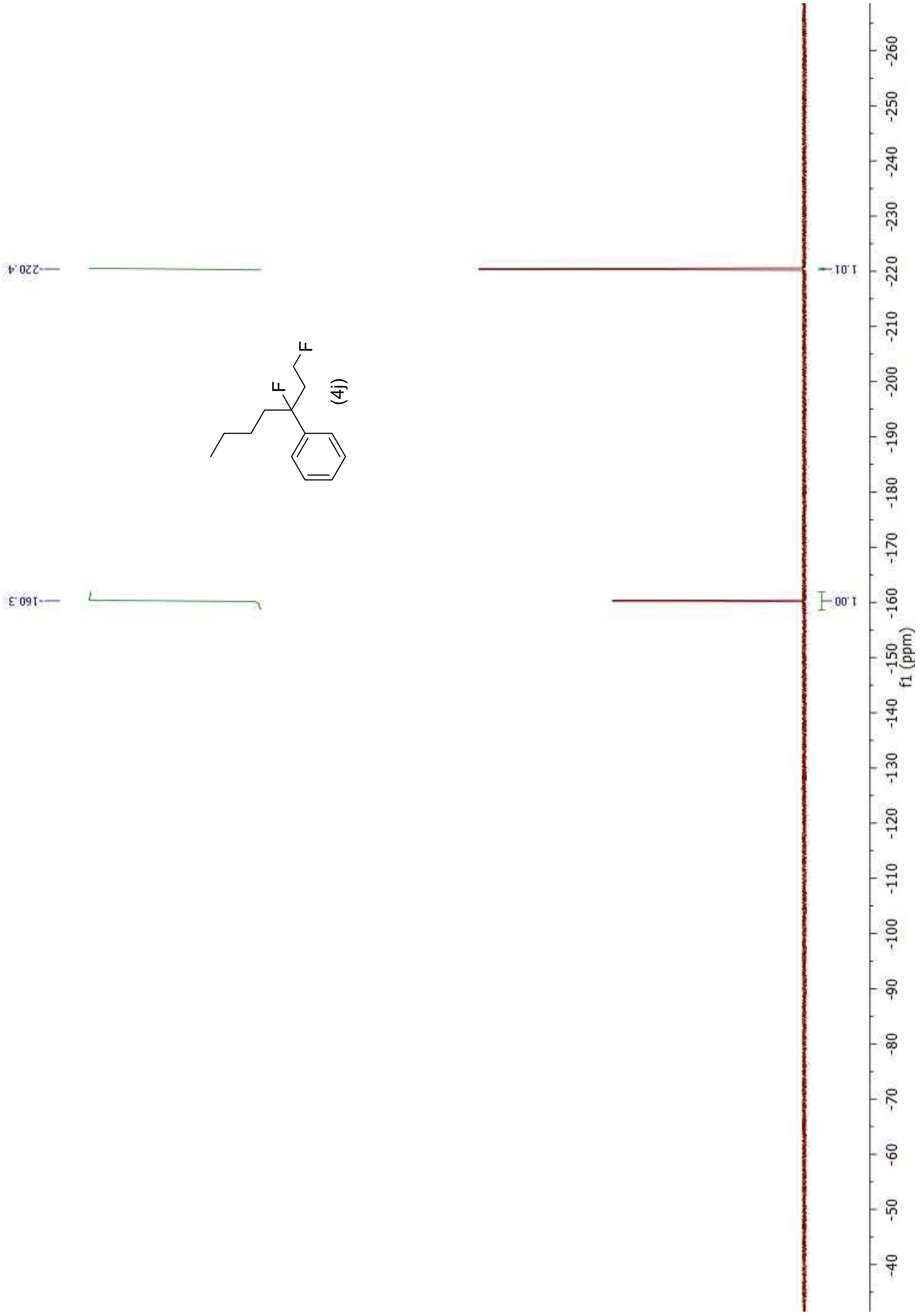
-220.4
-160.3
1.01
1.00
F
F
(4j)
f1 (ppm)

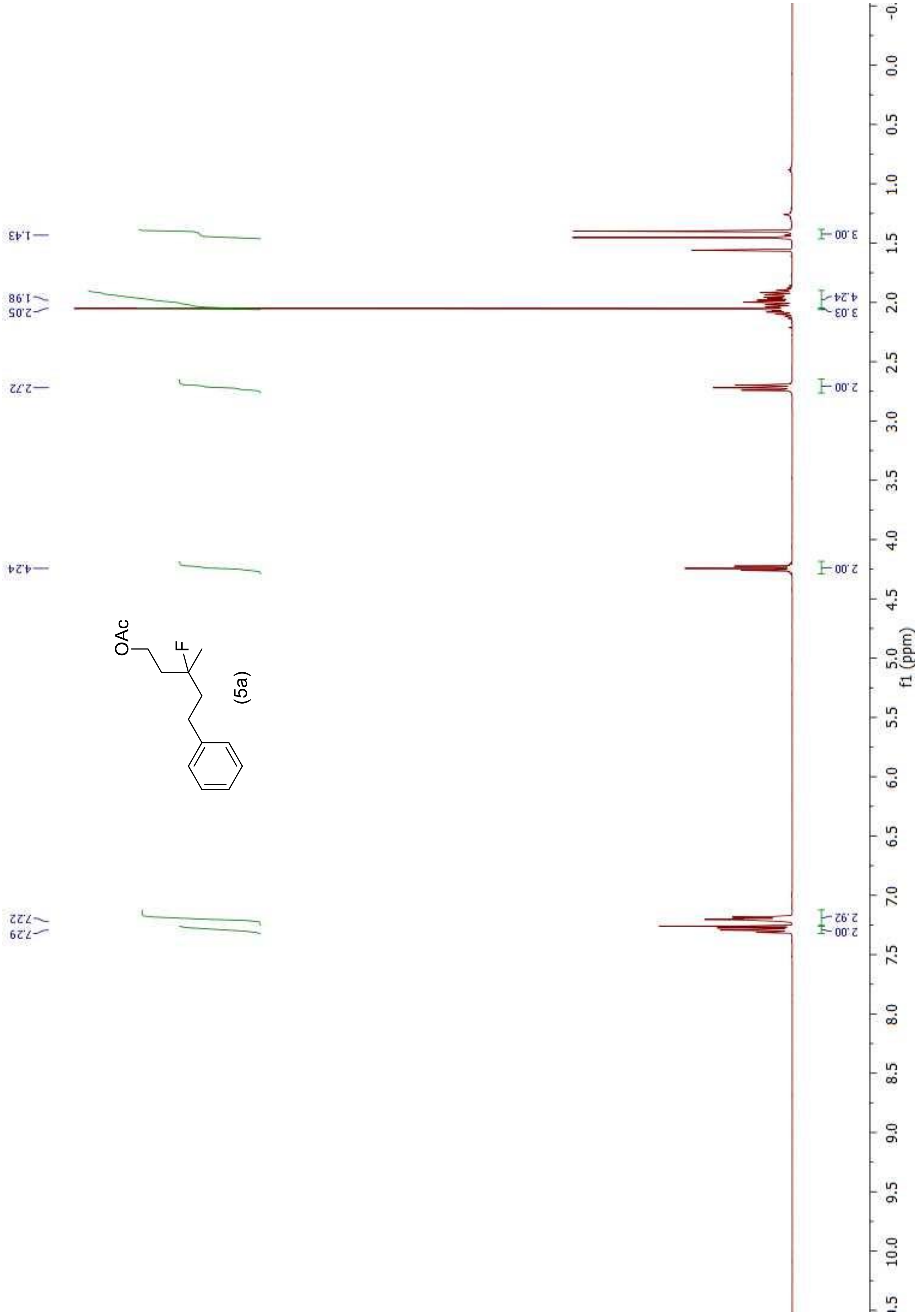
OAc
F
(5a)
f1 (ppm)

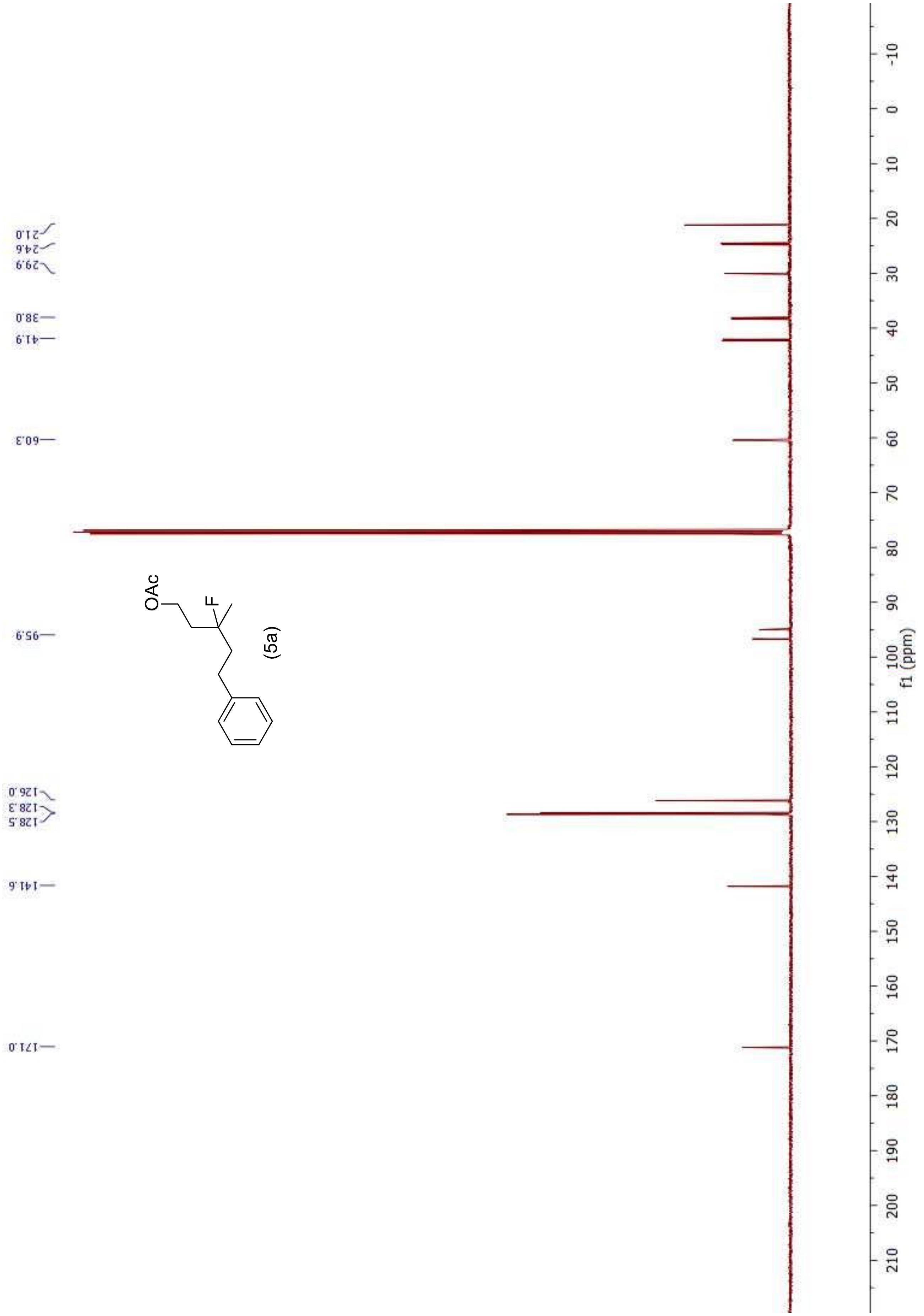
21.0
24.6
29.9
38.0
41.9
60.3
95.9
126.0
128.3
128.5
141.6
171.0
OAc
F
(5a)
f1 (ppm)

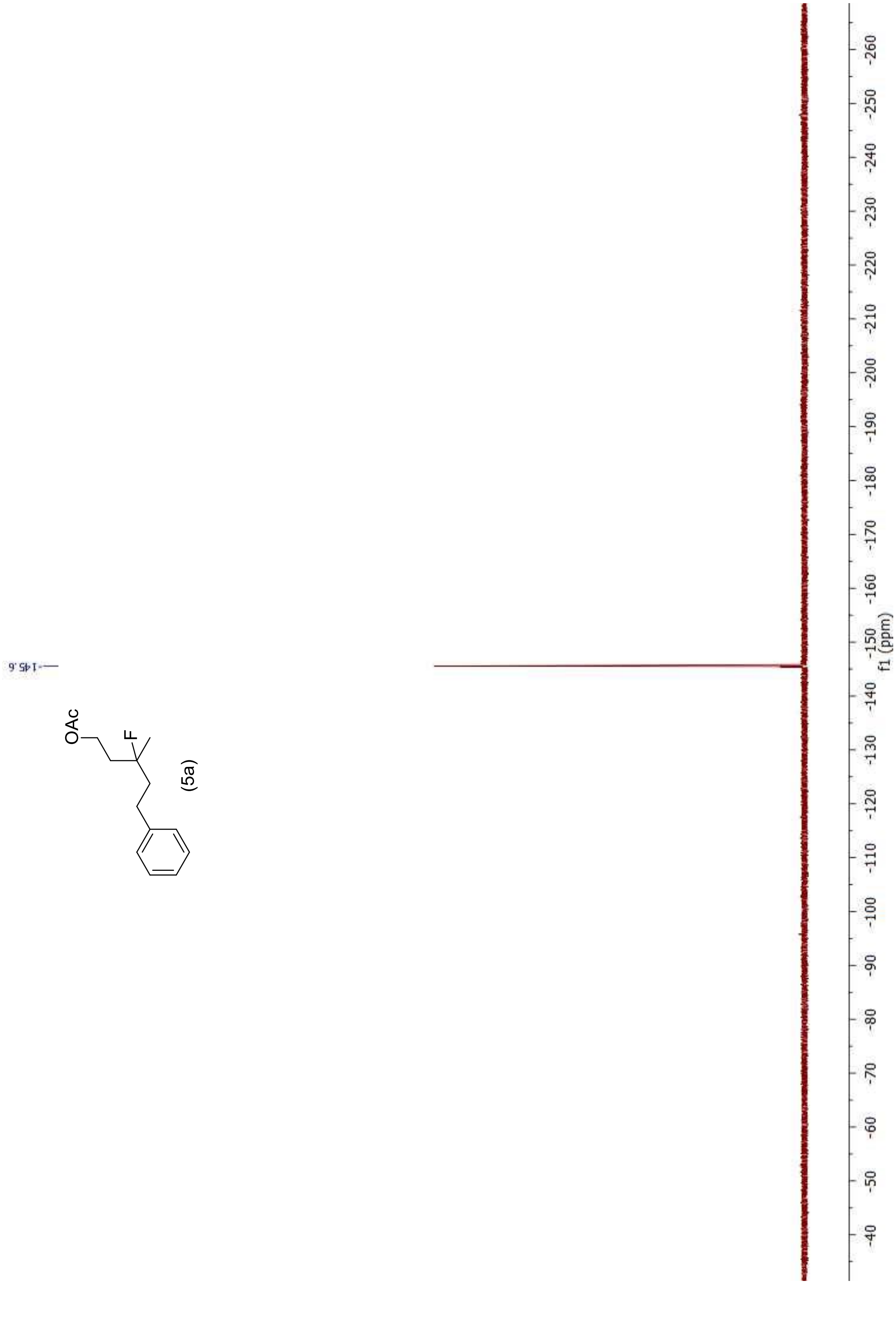
-145.6
OAc
F
(5a)
f1 (ppm)
-260
-250
-240
-230
-220
-210
-200
-190
-180
-170
-160
-150
-140
-130
-120
-110
-100
-90
-80
-70
-60
-50
-40

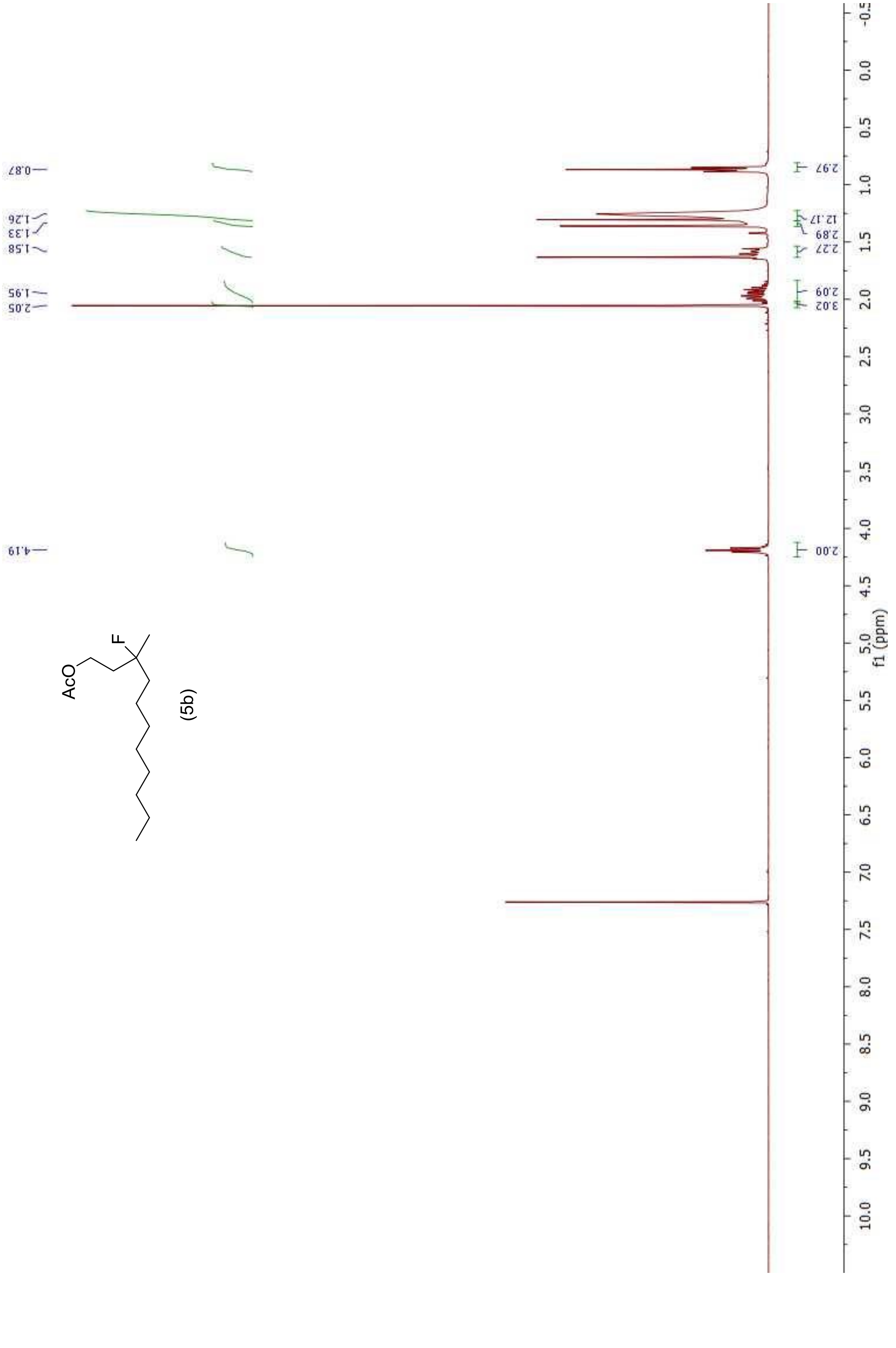

(5b)

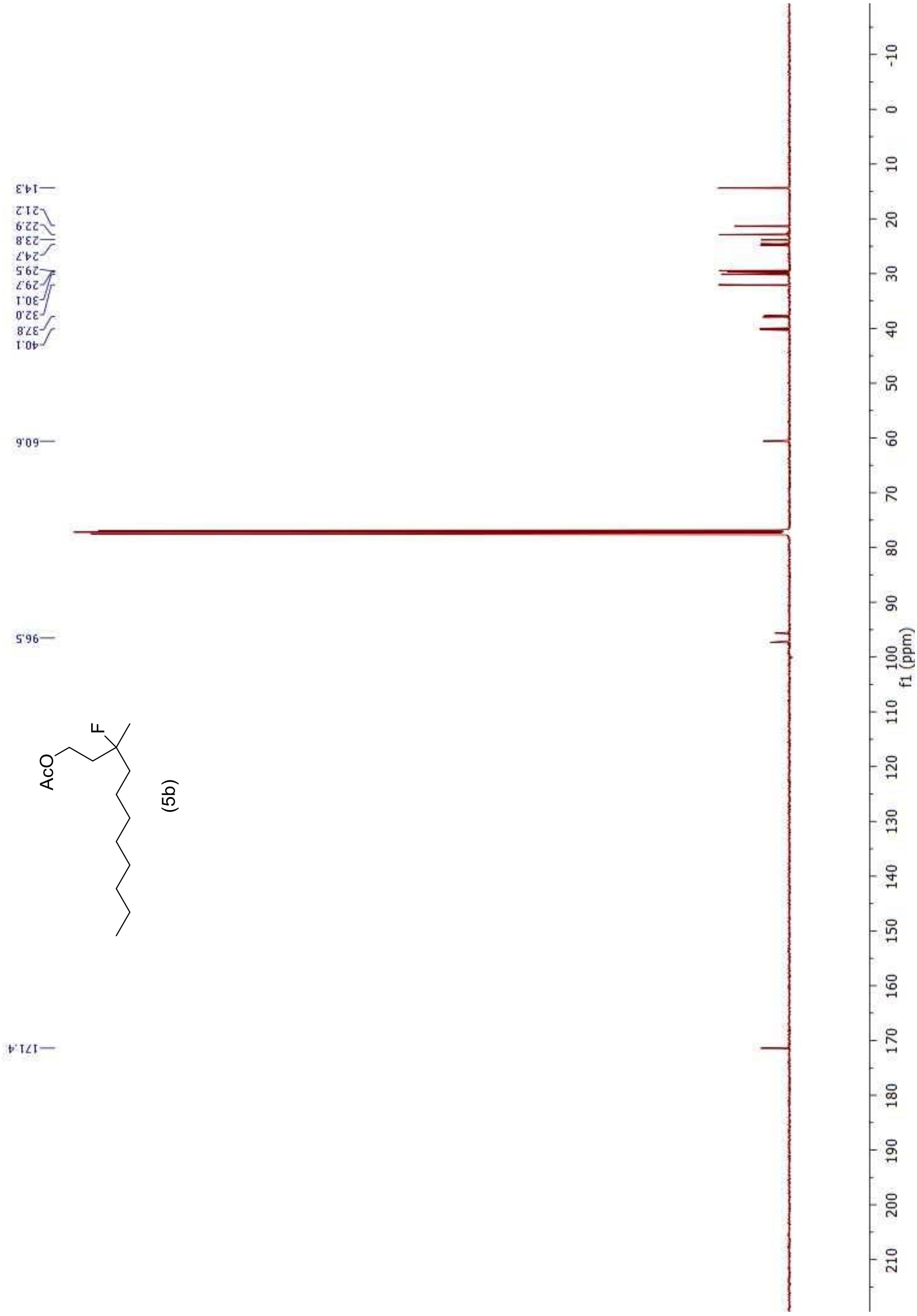
14.3
21.2
22.9
23.8
24.7
29.5
29.7
30.1
32.0
37.8
40.1
60.6
96.5
171.4
AcO
F
(5b)
f1 (ppm)

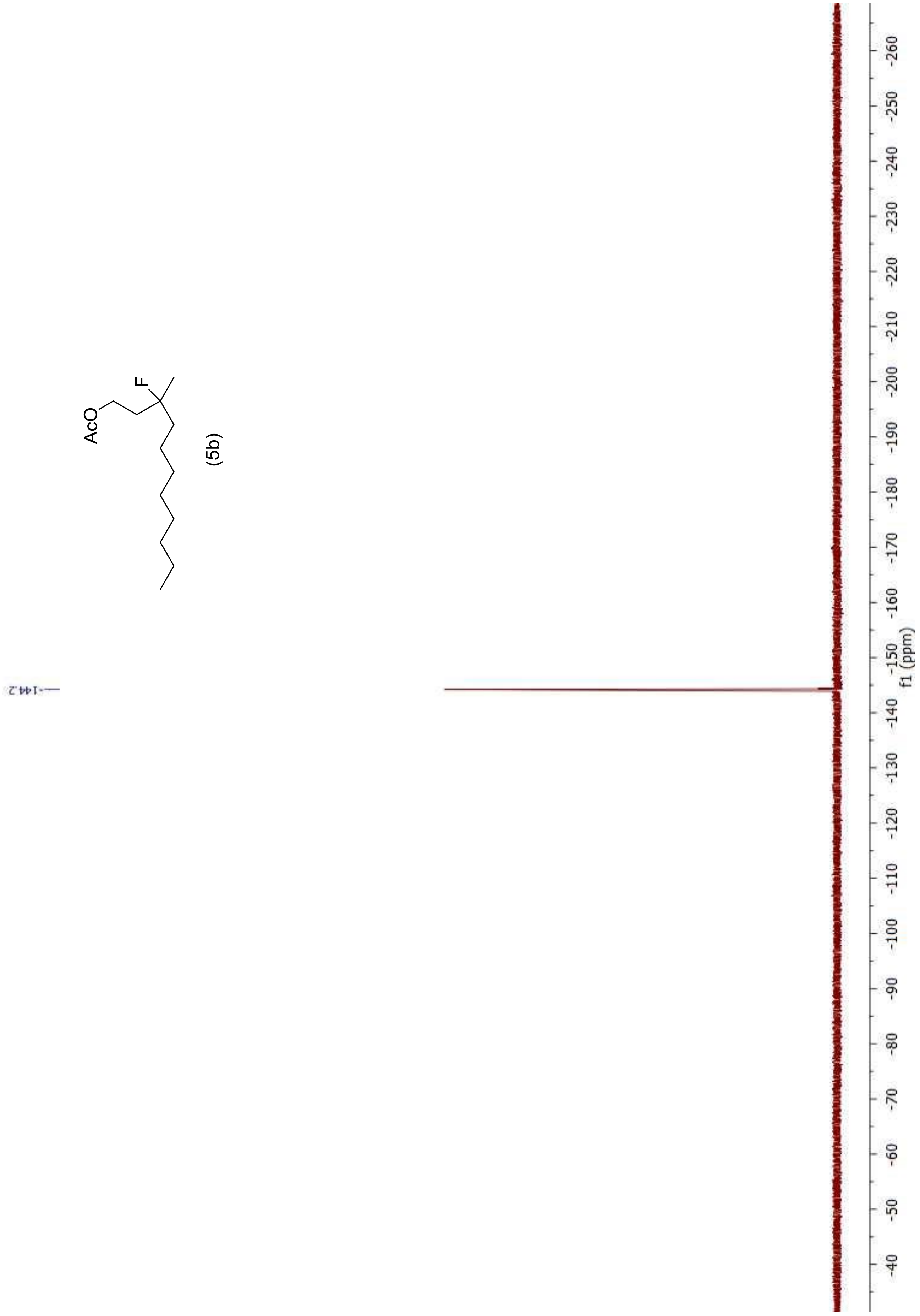
AcO
F
-144.2
f1 (ppm)
-260
-250
-240
-230
-220
-210
-200
-190
-180
-170
-160
-150
-140
-130
-120
-110
-100
-90
-80
-70
-60
-50
-40

(5b)

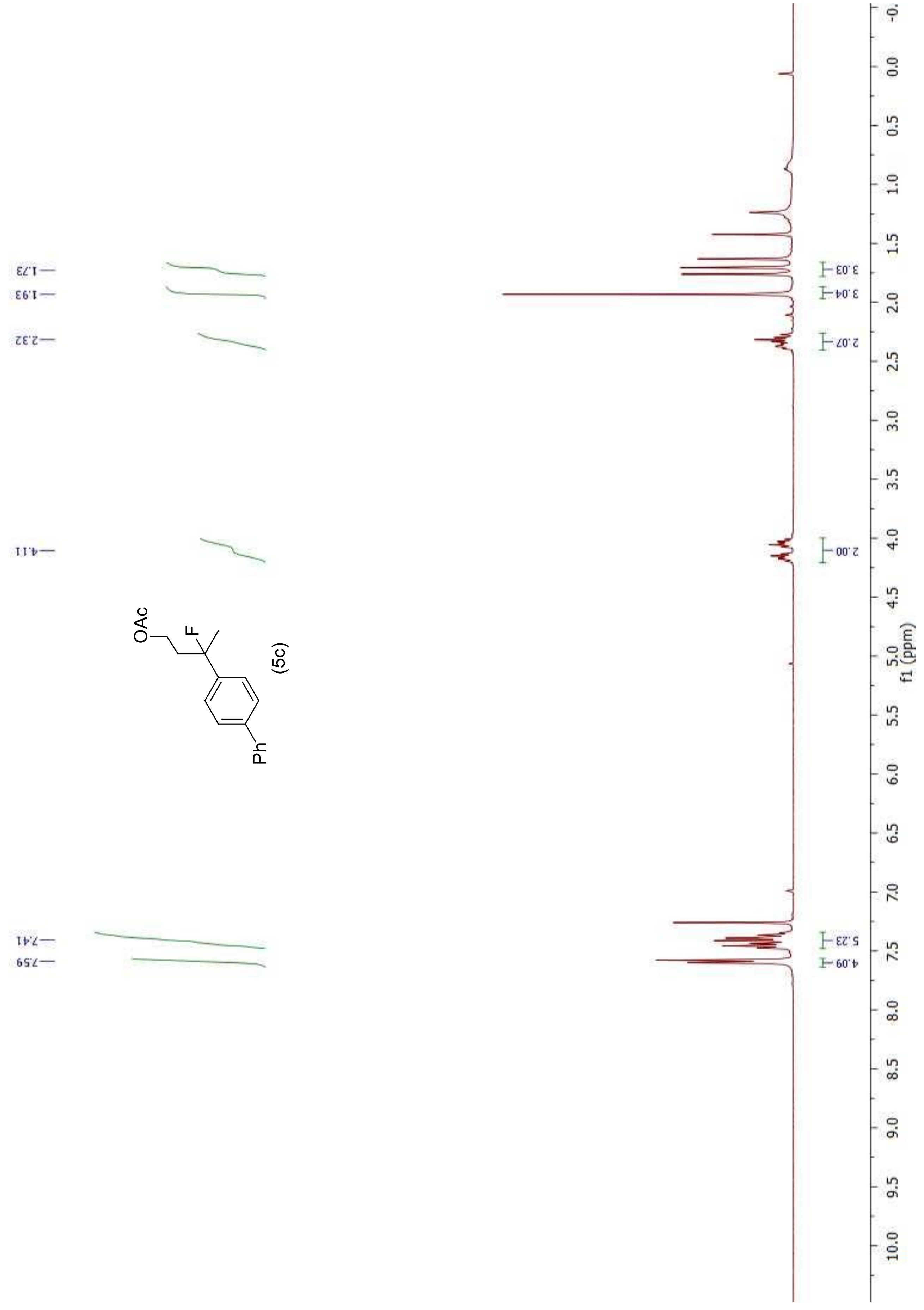

1.73
1.93
2.32
4.11
7.41
7.59
OAc
F
Ph
(5c)
3.03
3.04
2.07
2.00
5.23
4.09
f1 (ppm)

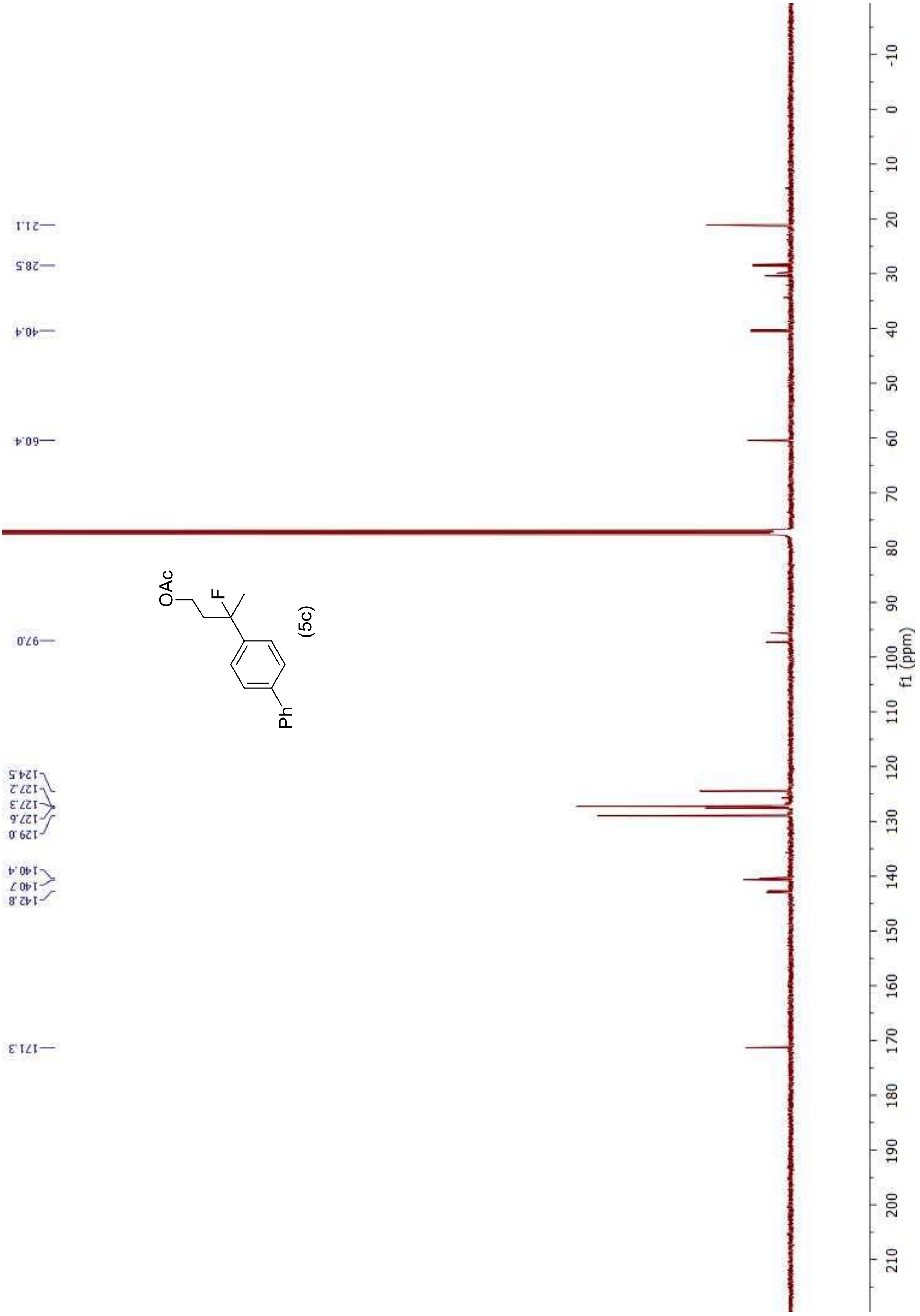
21.1
28.5
40.4
60.4
97.0
124.5
127.2
127.3
127.6
129.0
140.4
140.7
142.8
171.3
OAc
F
Ph
(5c)
f1 (ppm)

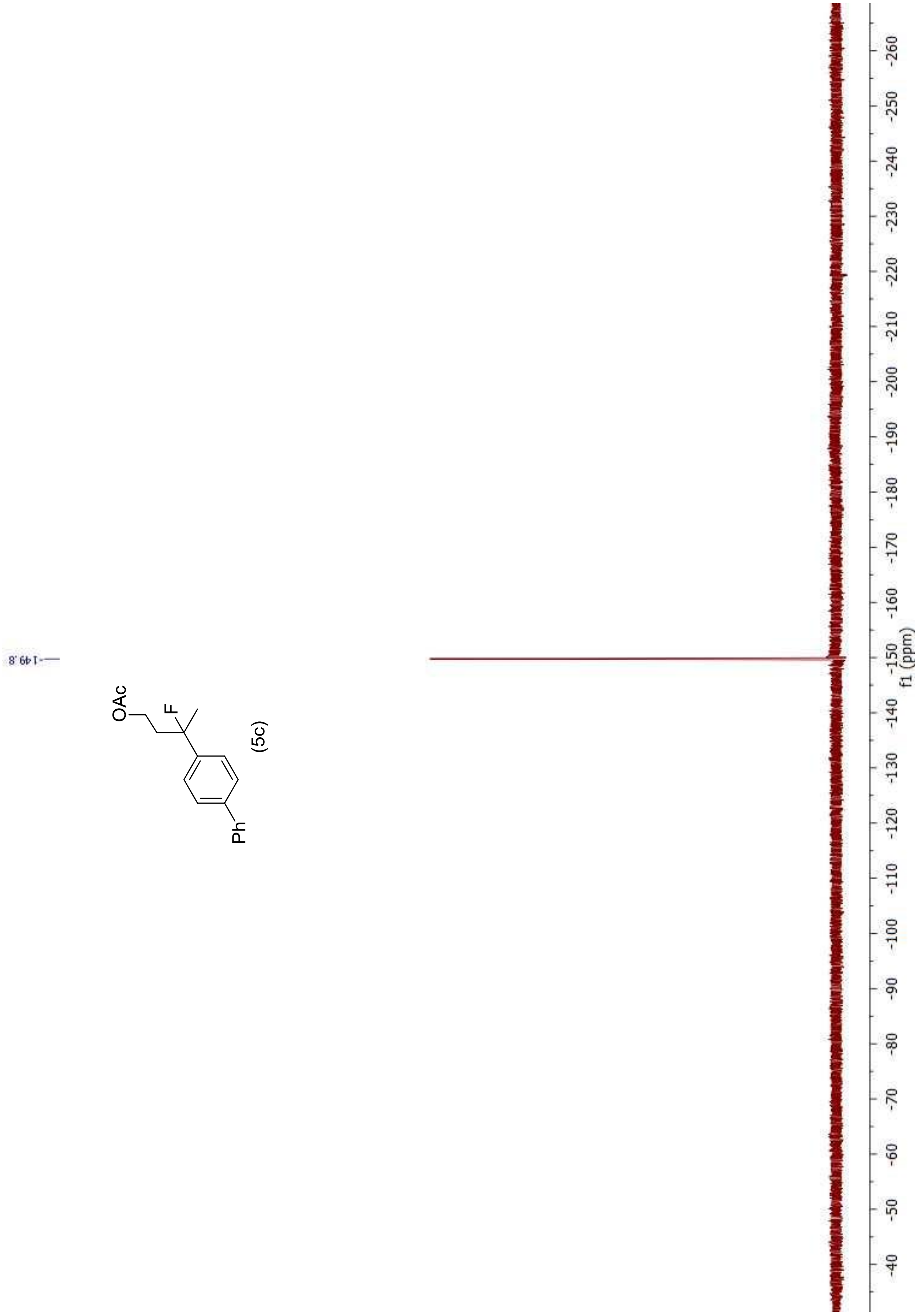

-149.8
OAc
F
Ph
(5c)
f1 (ppm)
-40
-50
-60
-70
-80
-90
-100
-110
-120
-130
-140
-150
-160
-170
-180
-190
-200
-210
-220
-230
-240
-250
-260

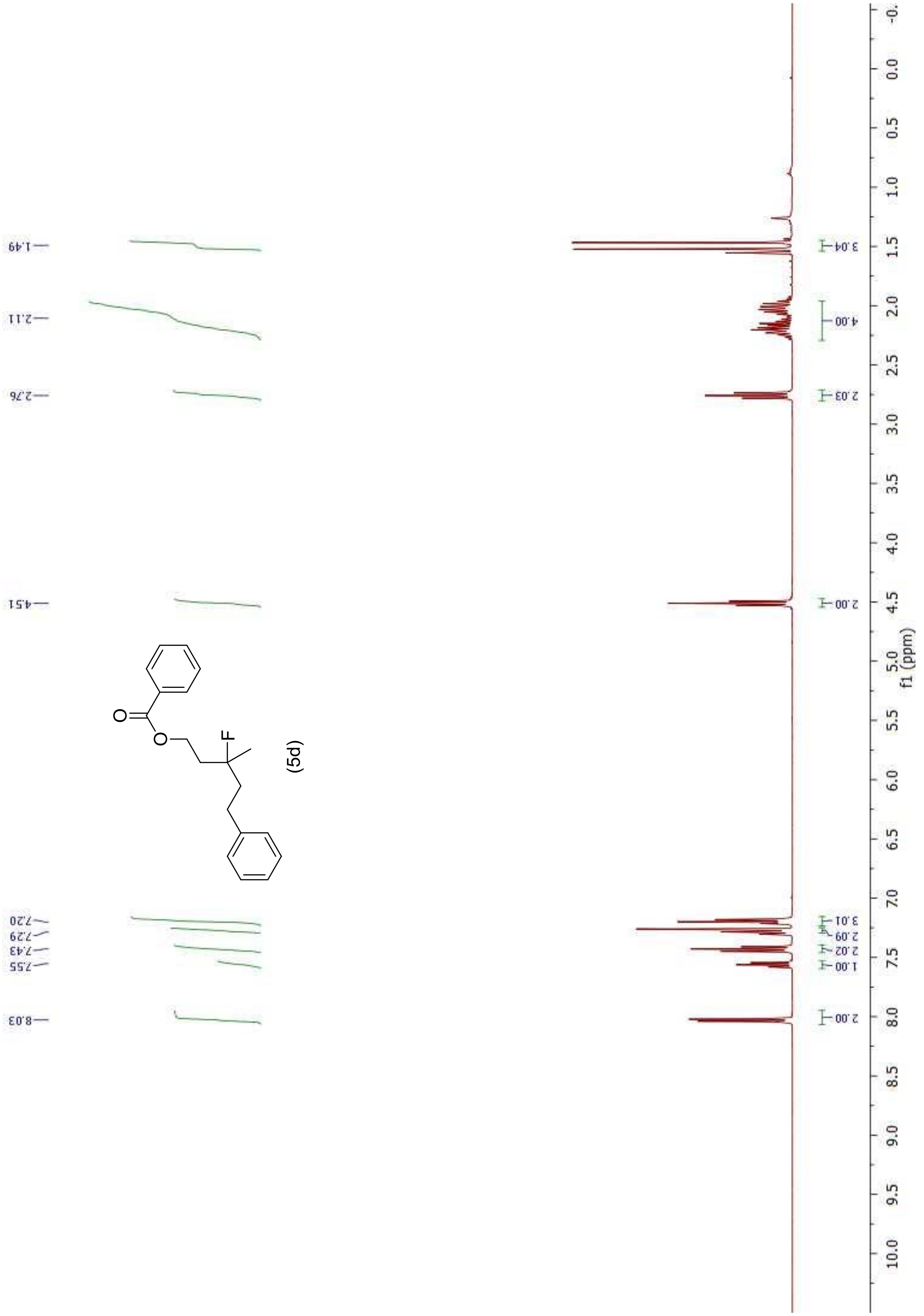
1.49
2.11
2.76
4.51
7.20
7.29
7.43
7.55
8.03
O
O
F
(5d)
3.04
4.00
2.03
2.00
3.01
2.09
2.02
1.00
2.00
f1 (ppm)

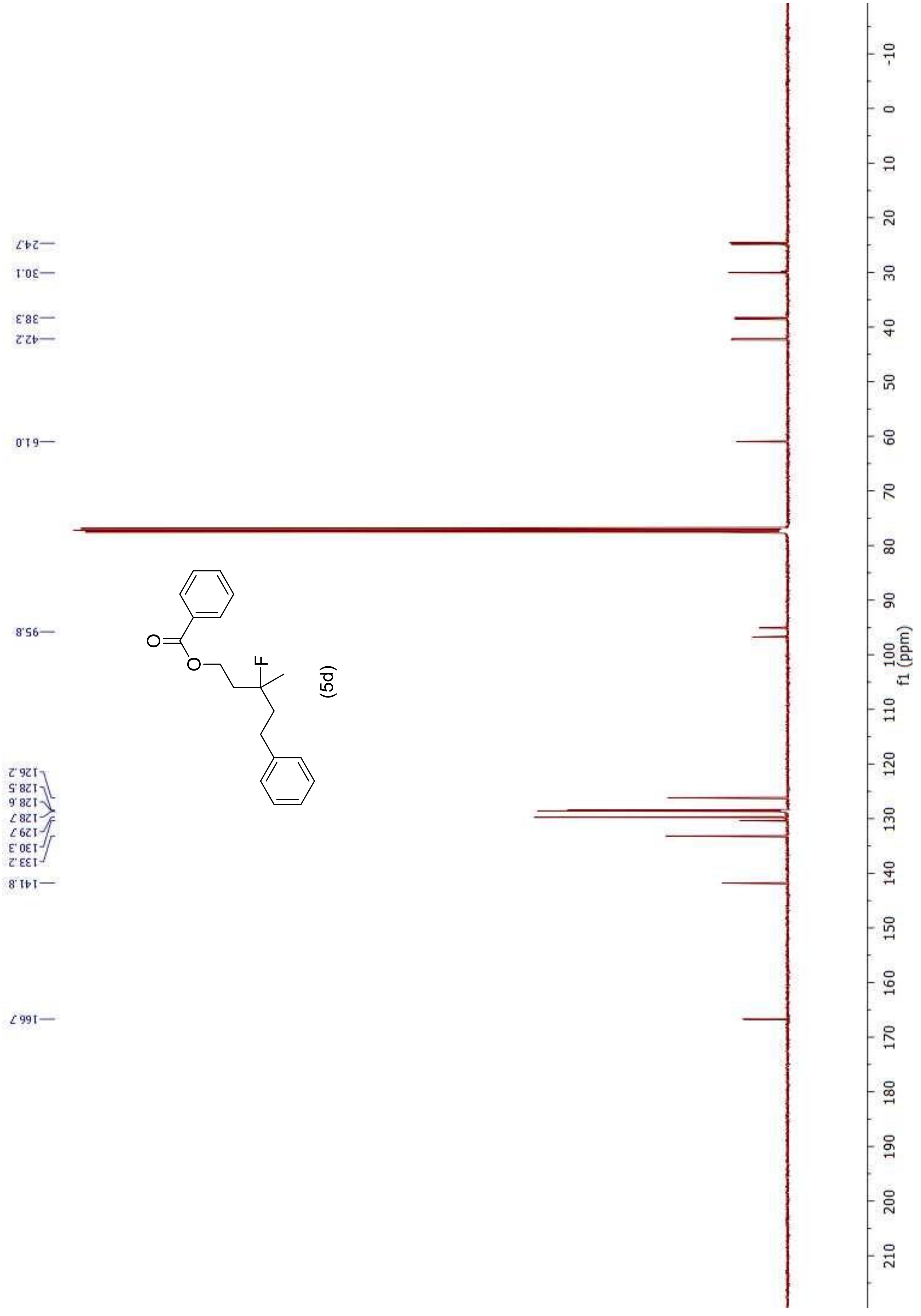
24.7
30.1
38.3
42.2
61.0
95.8
126.2
128.5
128.6
128.7
129.7
130.3
133.2
141.8
166.7
(5d)
F
O
f1 (ppm)

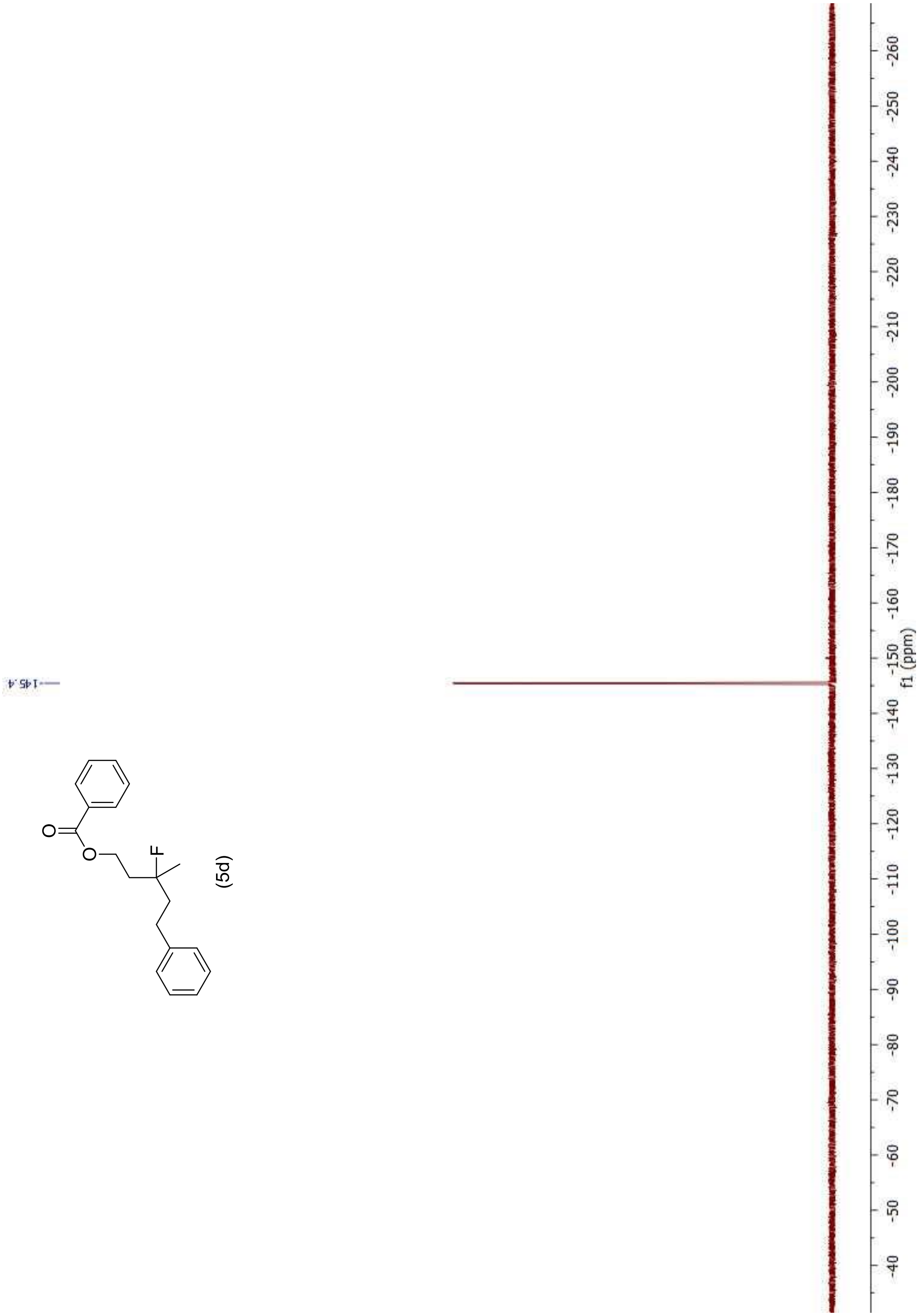

(5d)

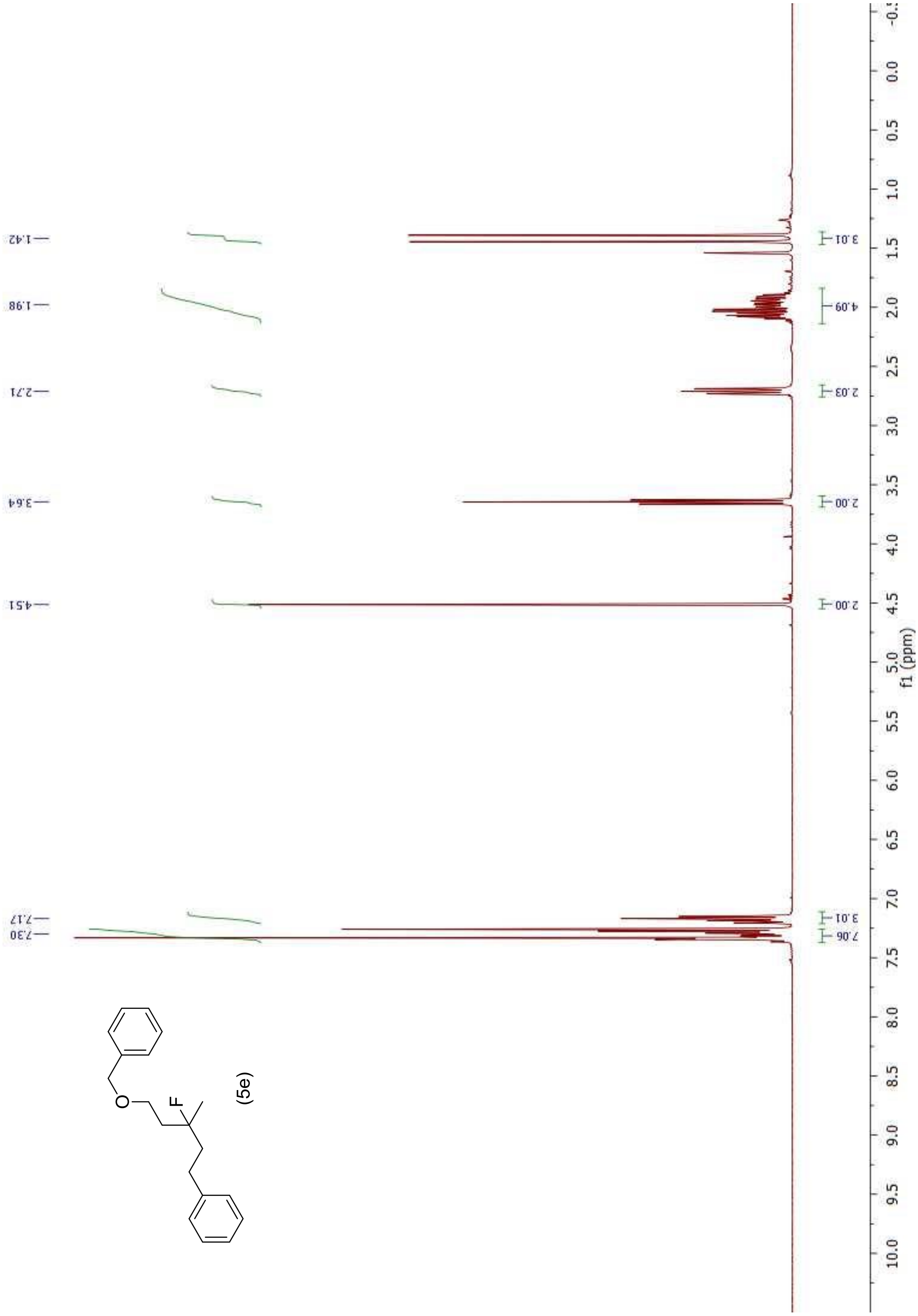
1.42
1.98
2.71
3.64
4.51
7.17
7.30
3.01
4.09
2.03
2.00
2.00
3.01
7.06
f1 (ppm)
(5e)
F
O

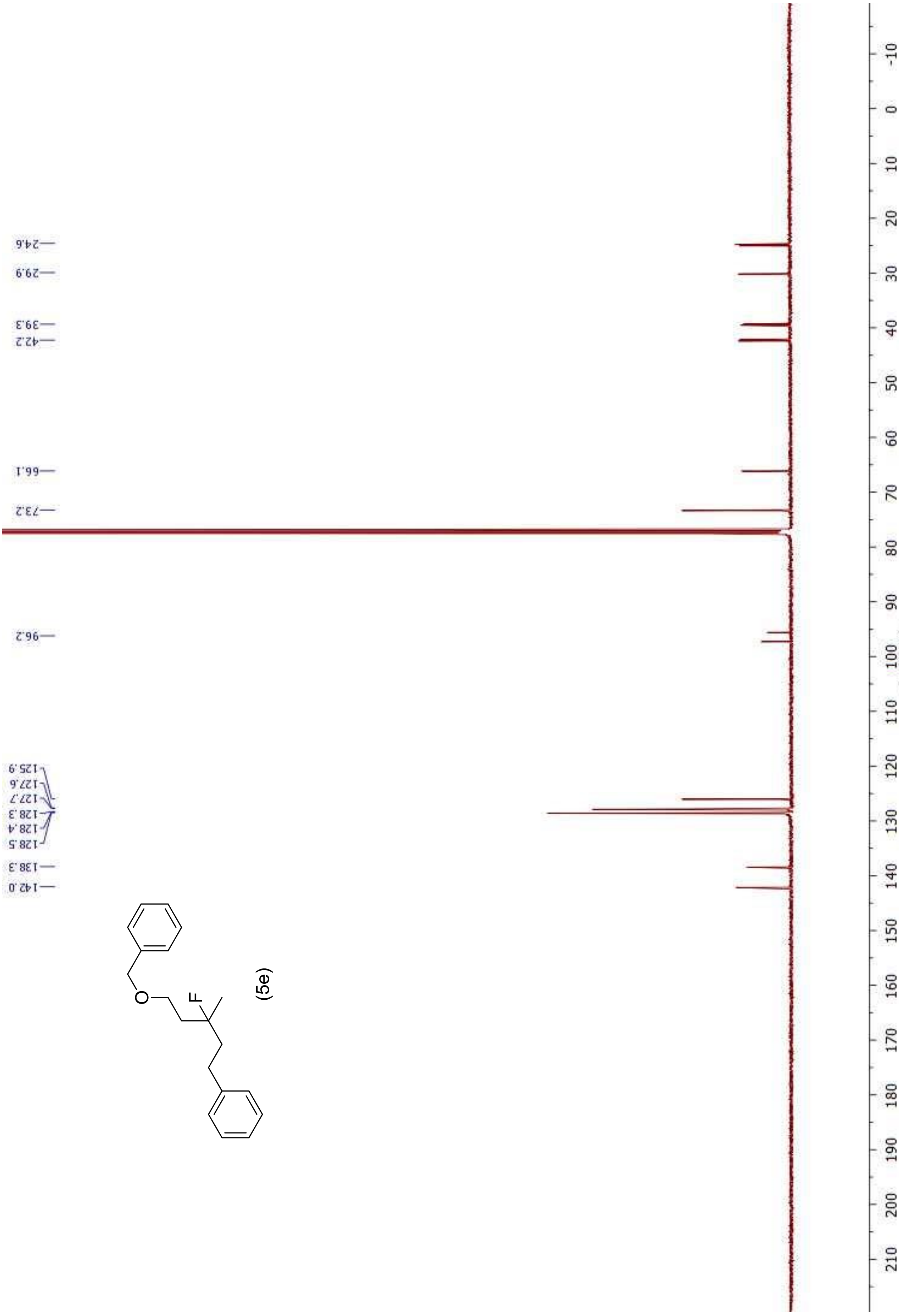
24.6
29.9
39.3
42.2
66.1
73.2
96.2
125.9
127.6
127.7
128.3
128.4
128.5
138.3
142.0
O
F
(5e)
-10
0
10
20
30
40
50
60
70
80
90
100
110
120
130
140
150
160
170
180
190
200
210
f1 (ppm)

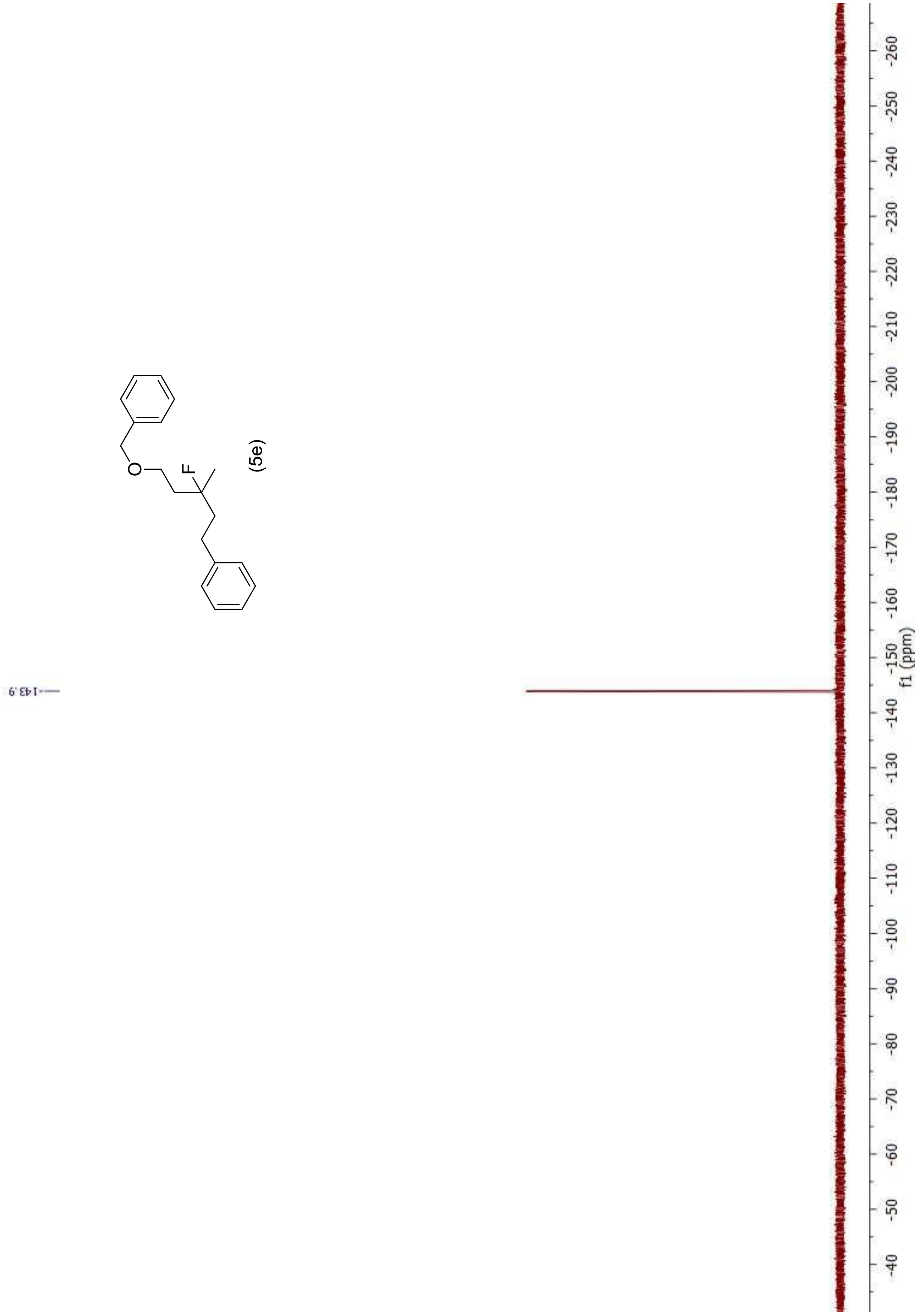
-143.9
f1 (ppm)
-40
-50
-60
-70
-80
-90
-100
-110
-120
-130
-140
-150
-160
-170
-180
-190
-200
-210
-220
-230
-240
-250
-260
O
F

(5e)

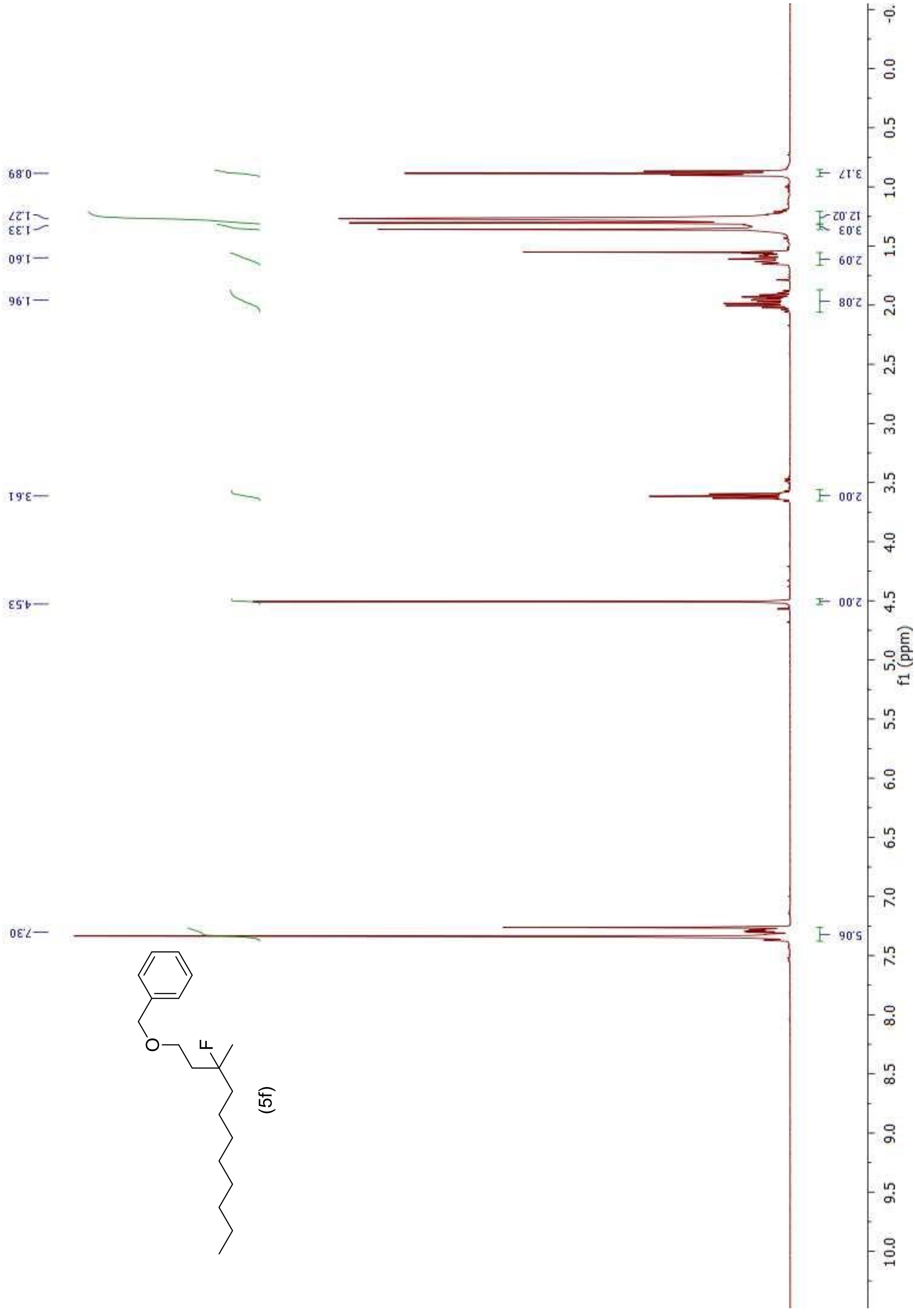
7.30
4.53
3.61
1.96
1.60
1.33
1.27
0.89
5.06
2.00
2.00
2.08
2.09
3.03
12.02
3.17
f1 (ppm)
F
O
(5f)

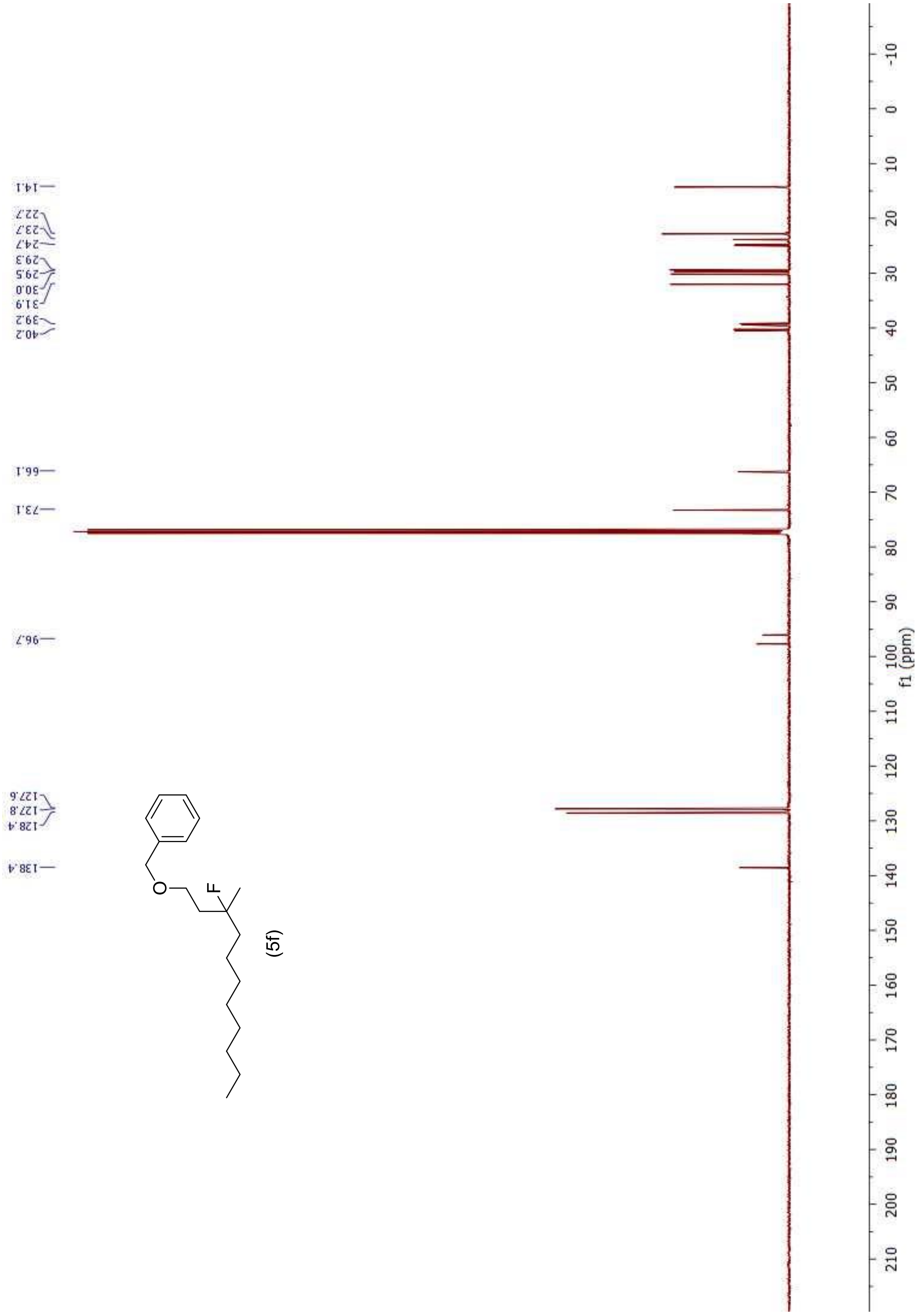
14.1
22.7
23.7
24.7
29.3
29.5
30.0
31.9
39.2
40.2
66.1
73.1
96.7
127.6
127.8
128.4
138.4
O
F
(5f)
f1 (ppm)
-10
0
10
20
30
40
50
60
70
80
90
100
110
120
130
140
150
160
170
180
190
200
210

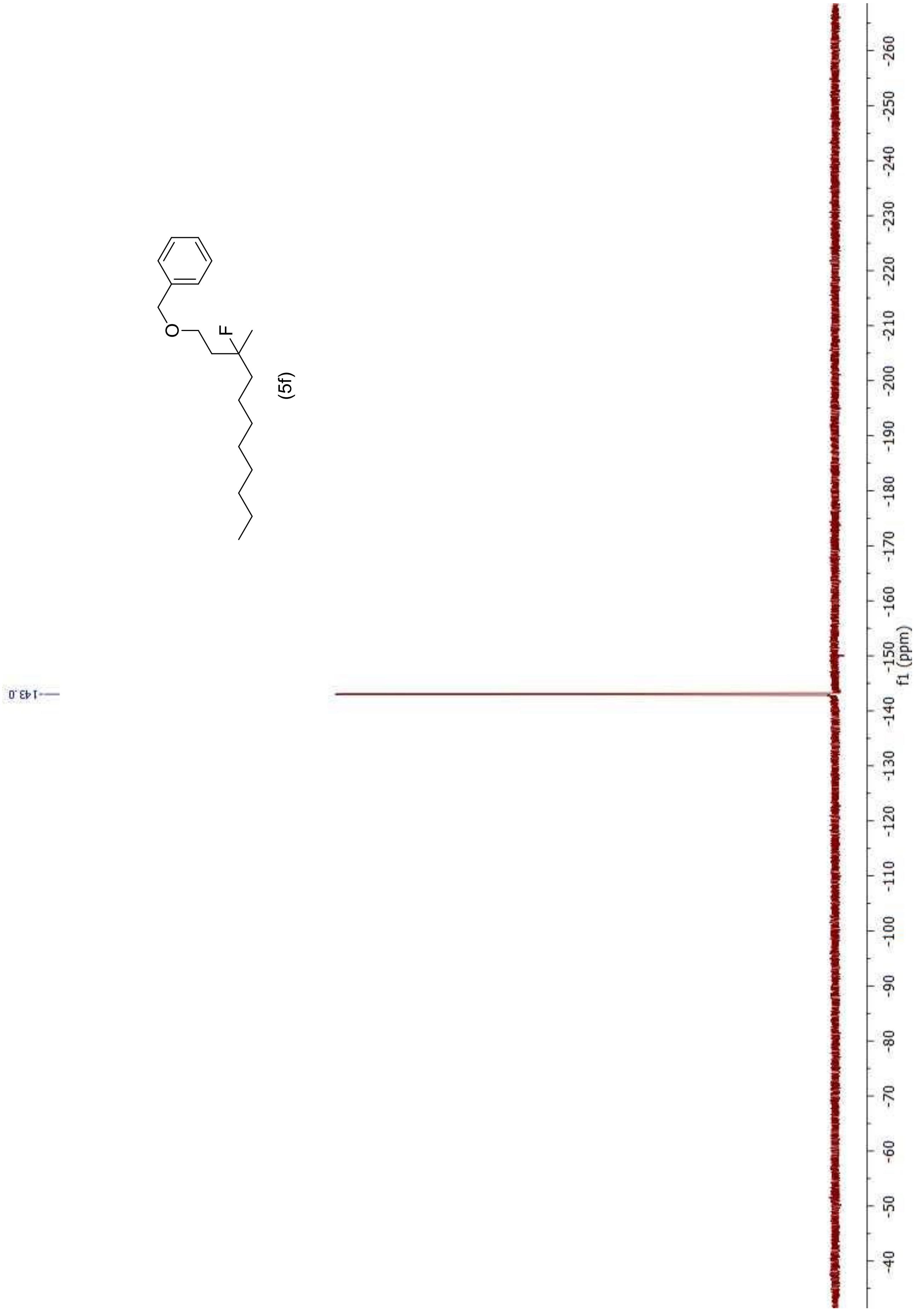

(5f)
F
O
-143.0
f1 (ppm)
-260
-250
-240
-230
-220
-210
-200
-190
-180
-170
-160
-150
-140
-130
-120
-110
-100
-90
-80
-70
-60
-50
-40

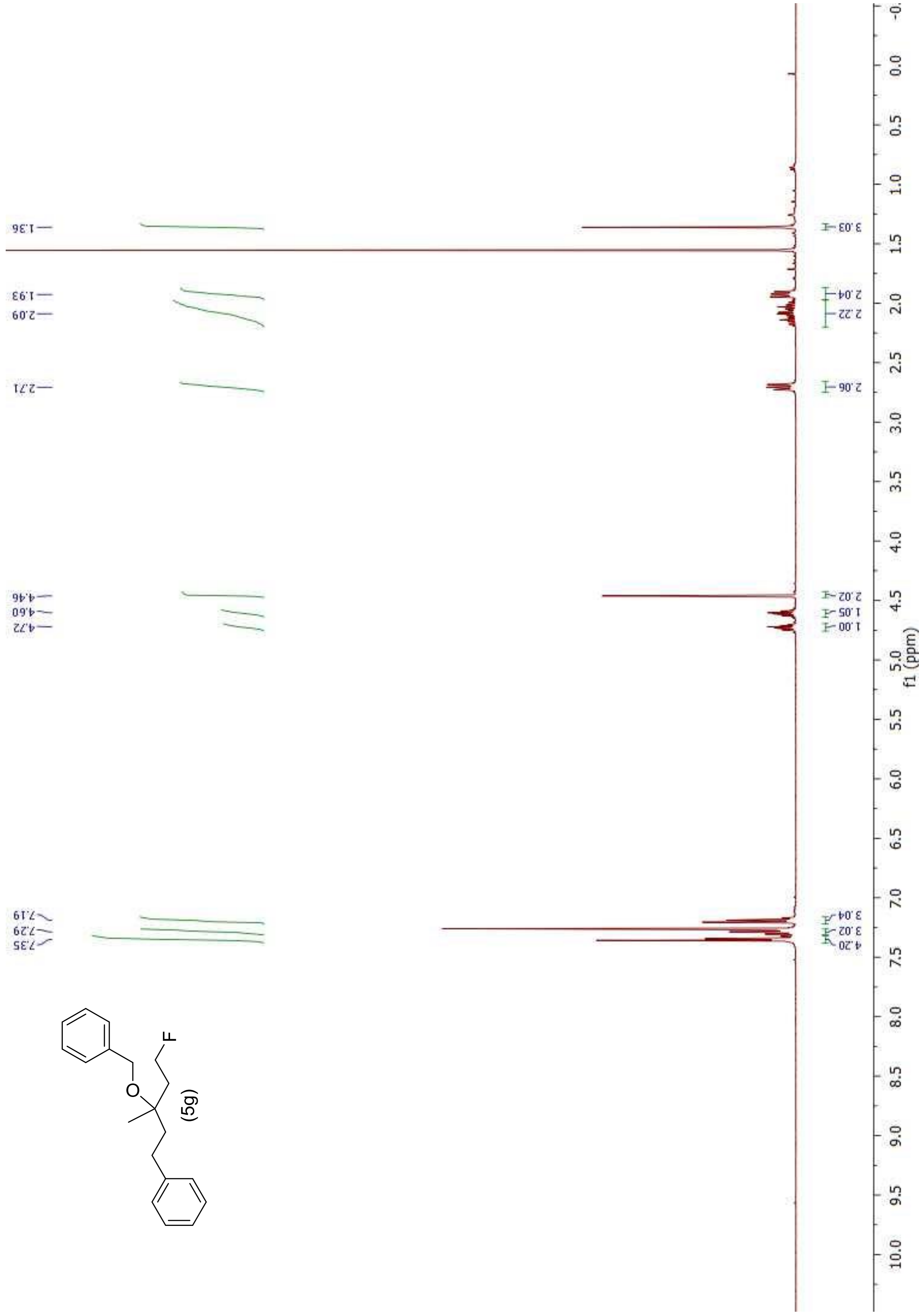
1.36
1.93
2.09
2.71
4.46
4.60
4.72
7.19
7.29
7.35
3.03
2.04
2.22
2.06
2.02
1.05
1.00
3.04
3.02
4.20
f1 (ppm)
O
F
(5g)

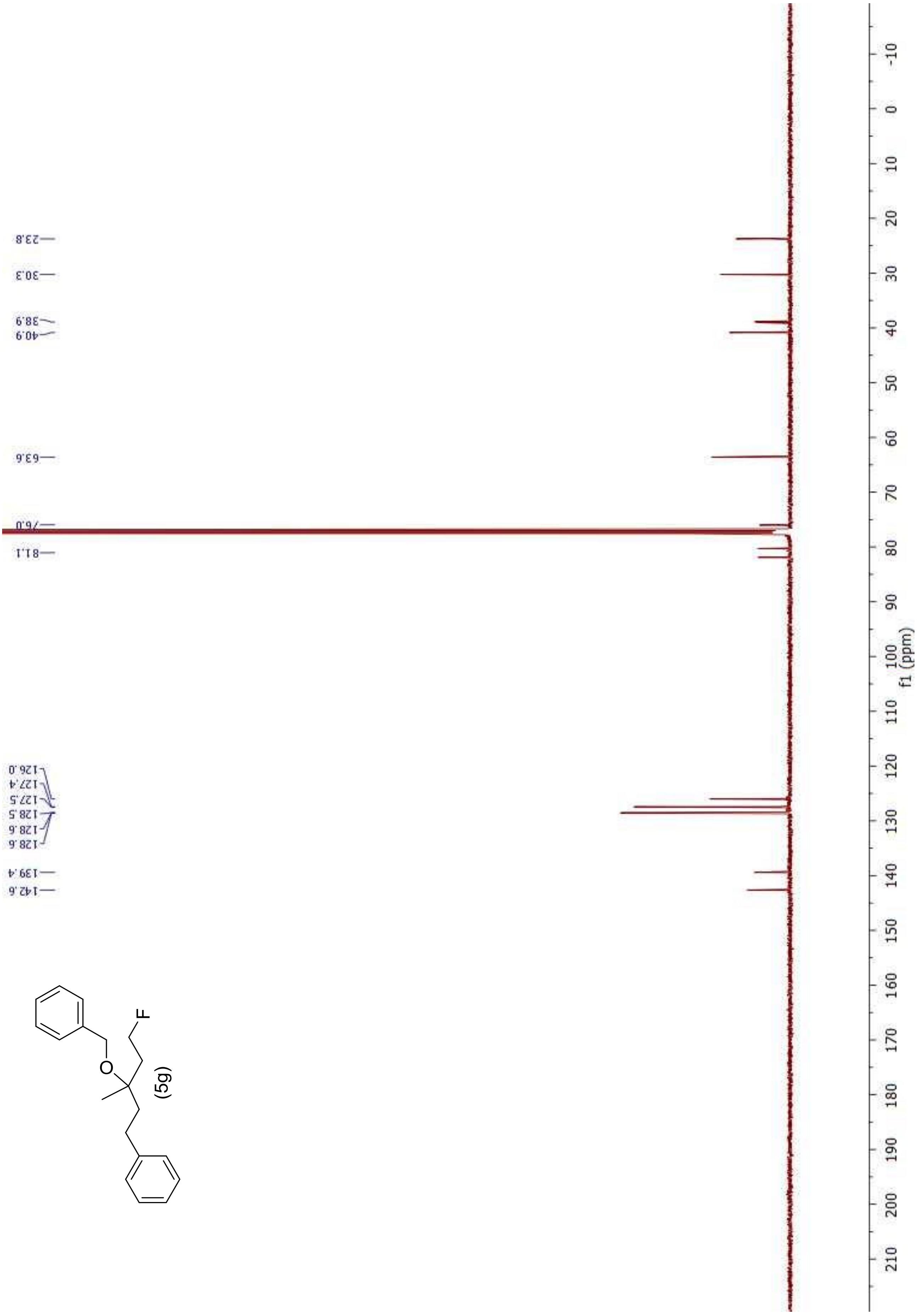
23.8
30.3
38.9
40.9
63.6
76.0
81.1
126.0
127.4
127.5
128.5
128.6
128.6
139.4
142.6
F
O
(5g)
f1 (ppm)

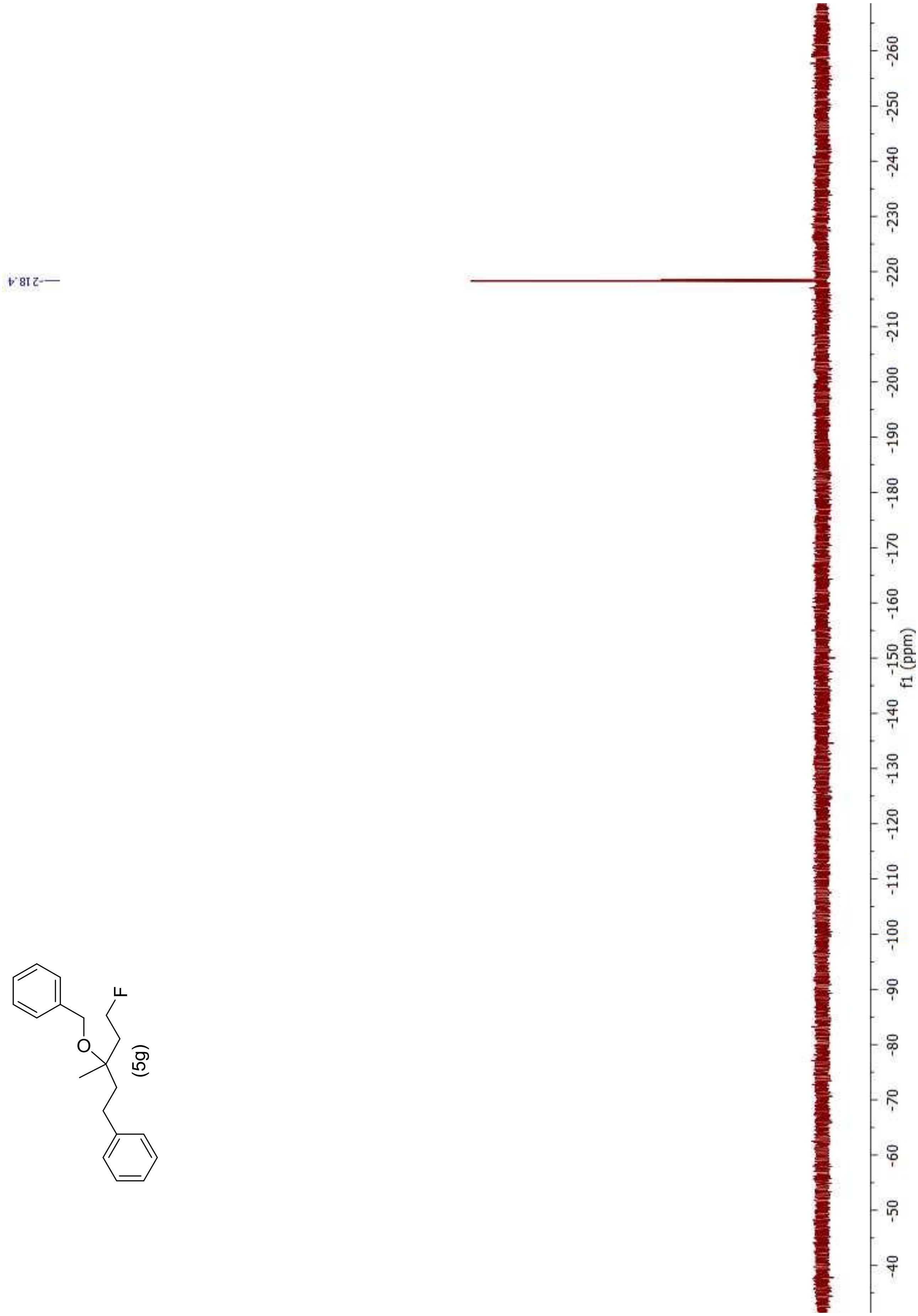
-218.4
f1 (ppm)
(5g)
O
F
